# The genomic organization and expression pattern of the low-affinity Fc gamma receptors (FcγR) in the Göttingen minipig

**DOI:** 10.1007/s00251-018-01099-1

**Published:** 2018-12-18

**Authors:** Jerome Egli, Roland Schmucki, Benjamin Loos, Stephan Reichl, Nils Grabole, Andreas Roller, Martin Ebeling, Alex Odermatt, Antonio Iglesias

**Affiliations:** 1 Pharma Research and Early Development (pRED), Pharmaceutical Sciences, Roche Innovation Center, Basel, Switzerland; 2 Division of Molecular and Systems Toxicology, Department of Pharmaceutical Sciences, University of Basel, Basel, Switzerland

**Keywords:** CD32, FcγRIIa, *FCGR* locus, Flow cytometry, Single-cell RNA sequencing, *Sus scrofa*

## Abstract

Safety and efficacy of therapeutic antibodies are often dependent on their interaction with Fc receptors for IgG (FcγRs). The Göttingen minipig represents a valuable species for biomedical research but its use in preclinical studies with therapeutic antibodies is hampered by the lack of knowledge about the porcine FcγRs. Genome analysis and sequencing now enabled the localization of the previously described FcγRIIIa in the orthologous location to human *FCGR3A*. In addition, we identified nearby the gene coding for the hitherto undescribed putative porcine FcγRIIa. The 1′241 bp long *FCGR2A* cDNA translates to a 274aa transmembrane protein containing an extracellular region with high similarity to human and cattle FcγRIIa. Like in cattle, the intracellular part does not contain an immunoreceptor tyrosine-based activation motif (ITAM) as in human FcγRIIa. Flow cytometry of the whole blood and single-cell RNA sequencing of peripheral blood mononuclear cells (PBMCs) of Göttingen minipigs revealed the expression profile of all porcine FcγRs which is compared to human and mouse. The new FcγRIIa is mainly expressed on platelets making the minipig a good model to study IgG-mediated platelet activation and aggregation. In contrast to humans, minipig blood monocytes were found to express inhibitory FcγRIIb that could lead to the underestimation of FcγR-mediated effects of monocytes observed in minipig studies with therapeutic antibodies.

**Electronic supplementary material:**

The online version of this article (10.1007/s00251-018-01099-1) contains supplementary material, which is available to authorized users.

## Introduction

Therapeutic antibodies of the IgG (immunoglobulin G) isotype represent an important group of new medical entities and interactions of Fc gamma receptors (FcγRs) with the Fc part of IgG antibodies are crucial in the antibody-based immunotherapy. Most mammals were shown to have three functionally distinct classes of FcγRs with different affinities and properties. FcγRIa (CD64) is capable of binding to free IgG antibodies and is hence considered as a high-affinity receptor. Its expression and function are conserved in most mammalian species, including pigs (Akula et al. 2014; van der Poel et al. 2011). Low-affinity receptors efficiently bind immune complexes and are divided into inhibitory and activating FcγRs. The structure and function of FcγRIIb (CD32b), the inhibitory low-affinity receptor, is also highly conserved in humans, pigs, mice, and other mammalian species (Akula et al. 2014). FcγRIIIa (CD16a) is an activating low-affinity FcγR that requires the association with FcR γ-chain (Fc receptor common gamma chain) for signaling (Kim et al. 2003). Different affinities to IgG were observed for the human FcγRIIIa V158F polymorphism within the extracellular domain (ECD). It was shown to be associated with differential response to therapeutic antibodies and disease progression (Mellor et al. 2013). Although FcγRIIIa is the most widely analyzed Fc receptor in pigs (Halloran et al. 1994), its gene structure and genetic localization has not yet been determined. In mouse, the orthologous receptor of FcγRIIIa is known as FcγRIV (Nimmerjahn and Ravetch 2006). FcγRIIa (CD32a) is another activating low-affinity receptor present in humans, non-human primates (NHPs), cattle, and rat and named as FcγRIII in mouse (Lux and Nimmerjahn 2013). In humans, FcγRIIa is expressed on the cell surface of monocytes, neutrophils, macrophages, eosinophils, basophils, dendritic cells, and platelets. It is involved in the process of phagocytosis, antibody-dependent cellular cytotoxicity (ADCC), and cytokine release (Powell and Hogarth 2008). The FcγRIIa R131H polymorphism is associated with severity and progression of idiopathic pulmonary fibrosis and with response to rituximab therapy (Bournazos et al. 2010; Ziakas et al. 2016). Immune complexes binding to FcγRIIa on human platelets can lead to thrombus formation (Zhi et al. 2015) and ultimately to heparin-induced thrombocytopenia (Greinacher 2009). Despite its importance, the minipig FcγRIIa and its gene *FCGR2A* could not be identified yet.

The Göttingen minipig is increasingly used as a valuable animal model for preclinical pharmacology and drug safety studies. The high similarity to humans in terms of genetics, genomics, physiology, and anatomy makes the minipig a desired alternative to NHPs (Ganderup et al. 2012). Additionally, Göttingen minipigs have a controlled health status, are easy to handle, and need less food, space, and pharmacological products compared to domestic pigs and other non-rodent species (McAnulty et al. 2011). Minipigs mainly differ from domestic pigs in their growth range and size at sexual maturity but not in anatomical structures (Swindle et al. 2012). Regarding the immune system, no major differences between pigs and minipig have been reported so far but detailed studies are lacking (Descotes et al. 2018). The use of the minipig as an adequate species for toxicity and efficacy evaluation of therapeutic antibodies requires a detailed knowledge of the FcγR composition and their interaction with human IgGs. However, to date, the knowledge on the binding properties of porcine FcγR to human antibodies is still scarce. In addition, the number of low-affinity FcγRs existing in the minipig and the allocation of the *FCGR* genes in the corresponding locus of the Göttingen minipig genome was not conclusively determined. The latest version of the Göttingen minipig genome was generated by Heckel et al. by mapping of the whole genome-sequencing data on the Duroc pig genome *Sus scrofa* 10.2 (Heckel et al. 2015). There, *FCGR2B* was the only gene annotated in the low-affinity *FCGR* locus. Recently, the new assembly *Sus scrofa* 11.1 was released containing a more accurate view of the pig genome including this particular locus (Li et al. 2017).

In this paper, we describe the complete assembly of the genetic *FCGR* locus of the Göttingen minipig including the exact mapping of *FCGR3*. Additionally, we demonstrate the identification, sequence characterization, and genomic location of *FCGR2A*, and the expression of low- and high-affinity FcγRs in the Göttingen minipig across blood cell types.

## Materials and methods

### *FCGR* locus assembly and *FCGR* mapping

**Fig. 1:**
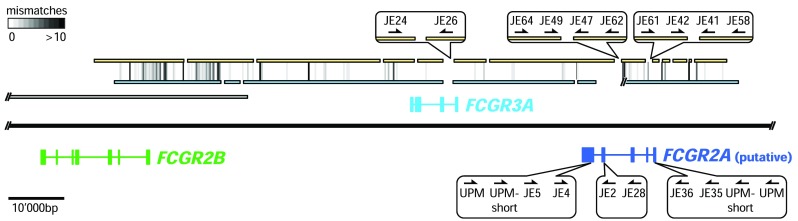
Genomic organization of the minipig *FCGR* locus. The black line represents the genomic sequence scaled as indicated in the lower left corner. *FCGR* genes are shown as colored lines with boxes representing the exon structure. Genes above and below the black line are encoded at the forward strand and the reverse strand, respectively. The sequence from the initial minipig genome draft containing *FCGR2B* (Heckel et al. 2015) is represented by a gray line. Yellow and blue lines represent whole genome shotgun contigs of the Göttingen minipig and the Wuzishan minipig, respectively. Vertical lines between the contigs of the two minipig breeds highlight regions with mismatches. The grayscale in the upper left corner indicates the number of mismatches found in intervals of 300 bp, from white (0 mismatches) to black (10 or more mismatches). Callouts enlarge the gaps now closed by sequencing using the primers indicated by the arrows. Refer to Online Resource 1 for primer sequences

The Göttingen minipig genome draft generated by Heckel et al. (2015) based on *Sus scrofa* 10.2 was used as a reference genome. Known sequences of *FCGR2B* and *FCGR3A* were blasted (Altschul et al. 1990) against whole genome shotgun-sequencing data of the Göttingen minipig (accession: AOCR01000000) and the Wuzishan minipig (accession: AJKK01000000) to identify overlapping contigs (contiguous sequences). A minimum of 95% identity over 200 base pairs was considered as sequence identity. The ends of each newly identified contig and exon sequences from known porcine *FCGR* genes were again blasted against the data from both minipig breeds to form longer contiguous sequences (Fig. 1). All sequences were continuously screened for potential *FCGR* genes by pairwise alignment (EMBOSS Water) to published porcine, human, and mouse *FCGR* exons.

Genomic DNA was isolated from the frozen spleen of a Göttingen minipig using the DNeasy Blood and Tissue Kit (Qiagen). PCR on genomic DNA with primers JE24/JE26 (see Fig. 1 for primer positions and Online Resource 1 for primer sequences) allowed sequencing of the gap within an intron of *FCGR3A* (Fig. 1) (GenBank ID: MH574548). The two remaining gaps in the putative *FCGR2A* introns were amplified by nested PCR using primers JE62/JE64 followed by JE47/JE49 and JE58/JE61 followed by JE41/JE42, respectively (Fig. 1, Online Resource 1). The obtained products were cloned using the TOPO TA cloning kit and sequenced (GenBank ID: MH574549, and MH574550). All sequencing reactions were performed by Microsynth.

### Identification and sequencing of putative porcine *FCGR2A*

Total RNA was isolated from blood cells of Göttingen minipigs and RNA integrity was determined on the Agilent 2100 Bioanalyzer System (Agilent Technologies). Then, putative *FCGR2A* cDNA ends were amplified in a nested PCR approach using SMARTer RACE 5′/3′ kit (Clontech). Rapid amplification of cDNA ends (RACE) PCR was performed by generation of 5′- and 3′-RACE-ready cDNA and subsequent PCR reactions using SMARTer RACE 5′/3′ kit (Clontech). More precisely, 5′- and 3′-RACE-ready cDNA was generated from total RNA serving as a template. In the first round of PCR, the supplied universal primer mix (UPM) was used together with primer JE5 or JE28, designed on predicted putative *FCGR2A* sequences. In a second round, nested UPM-short was used with primers JE4 or JE2 to generate 5′ or 3′ cDNA ends, respectively (Fig. 1, Online Resource 1). The products were analyzed on a 0.8% agarose gel and purified using the QIAquick gel extraction kit. Sanger sequencing was performed using several primers designed on predicted putative *FCGR2A* exons to identify the cDNA ends.

A final nested RT-PCR was performed on total RNA from minipig blood using first strand cDNA synthesis (New England Biolabs), the outer primers JE35/JE5, and the inner primers JE36/JE4 (Fig. 1, Online Resource 1). The product was cloned using the TOPO TA cloning kit and 30 colonies were sequenced from both sides using M13 and M13r primer. RACE PCR and RT-PCR sequences were assembled to generate the full-length transcript of the putative porcine *FCGR2A*.

### Sequence analysis and comparison

Signal sequences were predicted by similarity to porcine *FCGR2B* (Qiao et al. 2006) by signalP 4.1 Server (Nielsen 2017), SMART (Letunic and Bork 2018), and Sigcleave (von Heijne 1986). SMART also predicted the extracellular structures. Transmembrane (TM) helices were predicted from similarity to human FcγRIIa (Moi et al. 2010) and by the average result from the following prediction tools: TMpred (Hofmann and Stoffel 1993), DAS (Cserzo et al. 1997), SOSUI (Hirokawa et al. 1998), PredictProtein (Yachdav et al. 2014), Phobius (Kall et al. 2004), SMART, and ALOM (a program implemented at Roche according to Klein et al. (1985)).

For the phylogenetic tree, protein sequences were first aligned with MUSCLE (Edgar 2004) then poorly aligned positions and divergent regions were filtered with GBLOCKS (Castresana 2000) so that only the conserved ECD region remained. PHYLIP software package was used to calculate a protein sequence distance matrix followed by bootstrapping with 1000 replicates (Felsenstein 2005). Data was graphically displayed with the TreeExplorer software V2.12 (Jie 2017).

### Single-cell RNA sequencing

PBMCs were isolated using Ficoll-Paque Plus (GE Healthcare) and Leucosep tubes (Greiner bio-one, 12 mL) from K2 EDTA–treated whole blood of three different healthy human donors, Göttingen minipigs, or mice. Lysis buffer (BD Pharm Lyse) was used for subsequent removal of erythrocytes. Cell count and viability were determined using the Countess Automated Cell Counter (Invitrogen).

Single-cell capture was performed using the microfluidic chromium instrument (10x Genomics) capturing single cells in microdroplets. Cell suspensions containing approximately 4000 cells per sample from three different individuals were loaded together with the provided enzyme mix, beads, and oil. According to the manufacturer’s protocol, cDNA was generated, purified, and quality was checked on the Agilent 2100 Bioanalyzer System (Agilent Technologies). In a second step, a sequencing library was prepared by attaching Illumina Indices to fragmented cDNA strands. After size selection for approximately 500 bp fragments, library concentration was measured by a Qubit fluorometer (ThermoFisher). Every sample was adjusted to a final concentration of 2.5 nM, by dilution with buffer EB (Qiagen). All samples were pooled in same amounts. A PhiX solution was added, resulting in a spike-in amount of 1% in the final pool. Pooled fragments were denatured and mixed with a master mix consisting of EPX reagents 1–3 (Illumina), resulting in a final volume of 50 μL and a final concentration of 225 pM. After cluster generation, the flow cell was inserted into a HighSeq4000 instrument (Illumina). The sequencer cycle program consisted of 27 cycles for read one, 8 cycles for the index read and 99 cycles for read two.

Sequencing data were further processed using cell ranger version 2.0.0. First, fastq files were generated using the mkfastq function. Second, count files were generated using the count function. Human sequences were mapped against the genome assembly hg19, mouse sequences against the mm10, and minipig sequences against the RefSeq (reference sequence) (Pruitt et al. 2012) genome assembly *Sus scrofa* 11.1 containing all *FCGR* gene entries. Raw counts were further processed using an R (version 3.3.2) based in an in-house pipeline. First, data were imported using scater::read10XResults (version 1.6.3) function and QC parameters were calculated. The human raw cells were filtered using a minimum of 1.000 and a maximum of 50.000 umi (unique molecular identifier) counts in total. Second, cells having less than 300 genes expressed or more than 5% mitochondrial gene counts were filtered out. Mouse raw counts were filtered using a minimum of 700 and a maximum of 20.000 umi counts in total and at least 200 genes expressed. Finally, minipig raw counts were filtered using a minimum of 800 and a maximum of 20.000 umi counts in total and at least 200 genes expressed. Next, data were processed using the scater::normaliseExprs function using the 99th percentile for normalization. Confounding factors were determined based on their correlation to the first ten principle components of the normalized data. For human, we identified pct_counts_top_100_endogenous_features, log10_total_features, and donor; for mouse, we identified pct_counts_top_500_features and total_counts; and for minipig, we identified pct_counts_top_50_features, log10_total_counts, and donor as independent confounding factors. We applied a linear regression model to remove the effects of the identified confounders on the normalized data. Finally, we used the Seurat:: FindClusters function (version 1.4.0.16) and Seurat::RunTSNE function to run the t-SNE (t-distributed stochastic neighbor embedding) dimensionality reduction on selected features. Clusters were summarized according to the differential expression of various genes (Online Resource 2).

### Flow cytometry

Antibodies directed against porcine FcγRIIa (AbD29332.1) and FcγRIIa/b (AbD32591.1) were generated by Bio-Rad using the HuCAL technology. Generation and specificity of the HuCAL antibodies used here will be published elsewhere. Whole blood from three different Göttingen minipigs was collected in K2 EDTA–coated vacutainer tubes (BD). Erythrocytes were removed with the lysing buffer (BD Pharm Lyse) prior to staining of dead cells with amine-reactive dye Zombie Aqua (BioLegend). Leukocytes were then incubated in separate stainings with antibodies against porcine FcγRIIa (AbD29332.1), FcγRIIa/b (AbD32591.1), FcγRIIIa (CD16-PE, clone G7, Bio-Rad), and HuCAL Fab-A-FH-negative control antibody (AbD05930). Unlabeled HuCAL antibodies were then stained with a secondary PE-conjugated goat F(ab’)_2_ fragment anti-human IgG, F(ab’)_2_ fragment specific polyclonal antibody from Jackson ImmunoResearch. Cell events were acquired on BD LSRFortessa with BD FACSDiva and analyzed using FlowJo software.

## Results

### Localization of porcine *FCGR3A* and identification of putative *FCGR2A*

The low-affinity *FCGR* locus on chromosome 4 in the minipig genome draft based on *Sus scrofa* 10.2 was successfully supplemented with contigs from the Göttingen and the Wuzishan minipig and completed by PCR, cloning, and sequencing (Fig. 1, Online Resource 3). Sequences from the two minipig breeds differ in 0.31% mismatches and 1.25% indels spread over the total alignment comprising 115,000 nucleotides. The new assembly enabled the identification of exon sequences of *FCGR3A* in a forward orientation. Additionally, exon sequences were detected with high similarity to the porcine *FCGR2B* extracellular domain (ECD) and to porcine *FCGR3A* transmembrane/cytoplasmic (TM/C) region. These sequences belong to the putative porcine *FCGR2A* gene that is located in reverse orientation where the orthologue to human *FCGR2A* was expected (Fig. 1 and Fig. 4). Thus, the obtained sequence of the minipig low-affinity *FCGR* locus is completed and entirely contiguous. The newly characterized locus is highly similar to the most recent reference sequence (RefSeq) genome assembly of *Sus scrofa* 11.1 (Li et al. 2017).

**Fig. 2:**
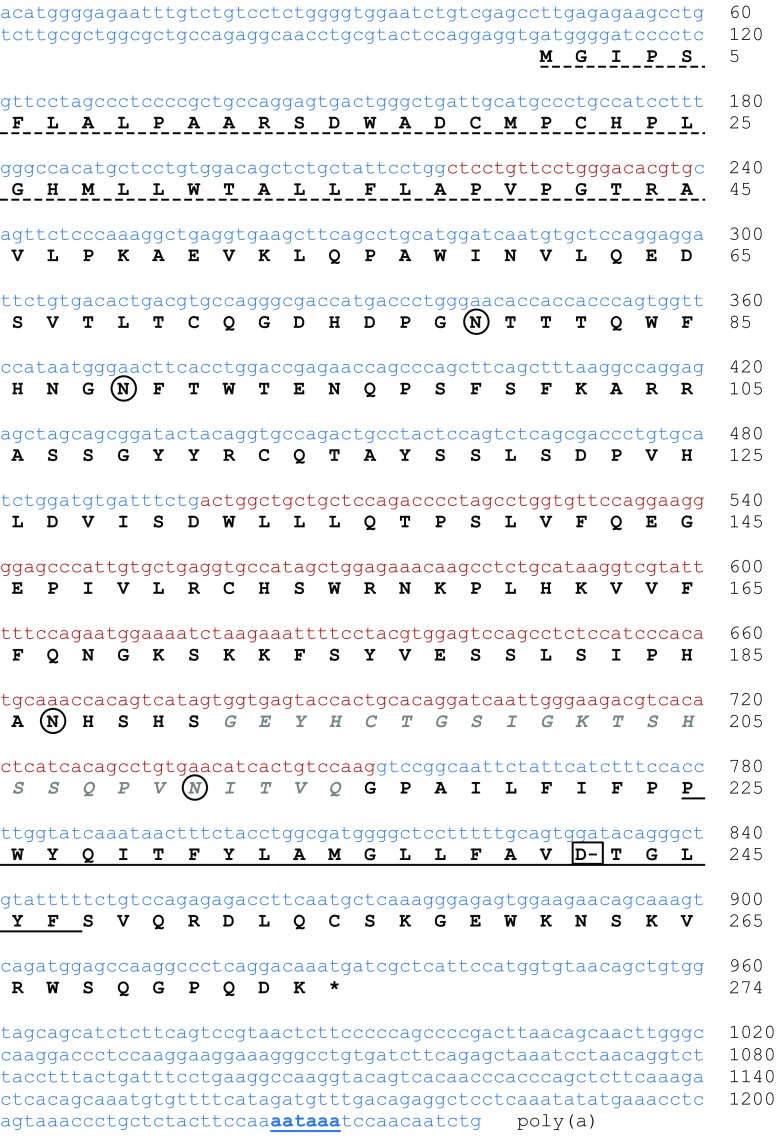
The sequence of putative porcine FcγRIIa mRNA is written in lower case letters with colors indicating alternating exons. In the 3′ untranslated region, the poly adenylation signal (aataaa) is underlined and bold. The amino acid sequence deduced from the ORF is written in capital letters below the nucleotide sequence. The predicted signal sequence is marked with a broken underline and the transmembrane (TM) spanning part is underlined. Letters in gray and italic mark the missing 24 amino acids observed in variant FcγRIIa1 and FcγRIIa3. All four potential N-glycosylation sites (N-X-S/T) are circled and the negatively charged aspartic acid residue in the TM domain, required for FcR γ-chain interaction, is indicated as "D-" in a box

Exon sequences of the putative porcine *FCGR2A* gene were disclosed from the low-affinity *FCGR* locus of the minipig by alignment of the sequences to porcine, human, and mouse *FCGR* exons. This enabled the design of gene-specific primers used for RACE PCR to identify cDNA ends. In combination with RT-PCR, we determined the complete sequence of the putative porcine *FCGR2A* transcript. The expected transcript, two potential polymorphisms, and three splice variants were identified in the total RNA preparation of one Göttingen minipig (Fig. 2) by Sanger sequencing of 30 clones.

The putative porcine *FCGR2A* cDNA is 1′241 bp long, contains an 822 bp open reading frame (ORF) translating to a 274 amino acids (aa) long protein (RefSeq No. XM_021089520.1). Bioinformatic analysis revealed a 45aa long signal peptide followed by an ECD region containing two immunoglobulin-like parts (Ig1, 74aa; Ig2, 78aa). Like porcine FcγRIIb, the ECD contains four potential *N*-glycosylation sites (Asn^79^, Asn^89^, Asn^187^, and Asn^211^) identified by the common motif (N-X-S/T) (Aebi 2013). The receptor sequence predicts a 23aa hydrophobic TM part with a negatively charged aspartic acid residue allowing interaction with the FcR γ-chain (Kim et al. 2003). In the 27aa long intracellular part, no immunoreceptor tyrosine-based activation motif (ITAM; Y-X-X-L/I) or immunoreceptor tyrosine-based inhibition motif (ITIM; S/I/V/L-X-Y-X-X-I/V/L) was found in contrast to human FcγRIIa or FcγRIIb, respectively (Isakov 1997; Ravetch and Lanier 2000) (Fig. 2).

The putative porcine FcγRIIa.1 variant revealed a 24aa deletion within the Ig2-like part of the ECD (Gly192_Gln215del) (Fig. 2). Further variants include FcγRIIa.2 lacking the whole Ig2-like part of the ECD (Asp131_Gln215del) and FcγRIIa.3 lacking the whole Ig1-like part of the ECD (Ala45_Ser130del) and bearing the 24aa deletion of FcγRIIa.1. Furthermore, four single nucleotide polymorphisms were detected, two of them affecting the coding sequence and thus representing potential polymorphisms. The A11S polymorphism is located in the signal sequence and the H205Y polymorphism in the Ig2-like part of the ECD.

**Fig. 3:**
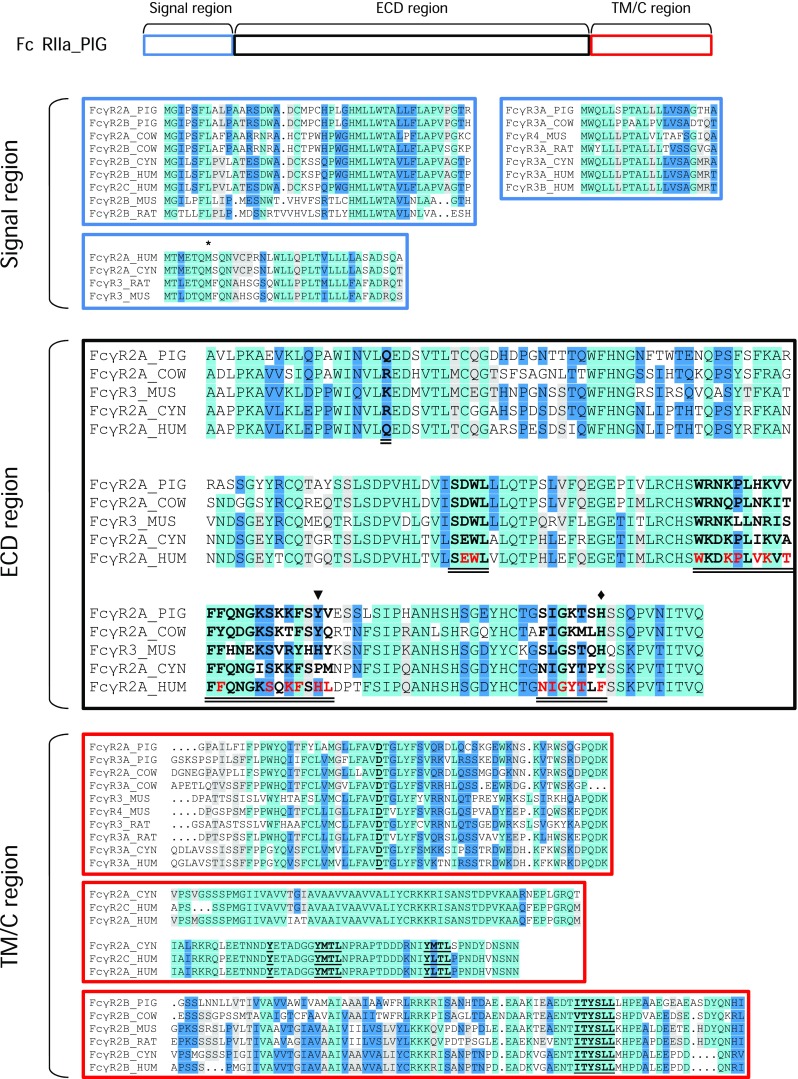
Comparison of FcγR protein sequences. A schematic representation of the putative porcine (PIG) FcγRIIa transcript is shown at the top. The boxes within the transcript represent signal regions (blue boxes), extracellular domains (ECD, black box), and transmembrane/cytoplasmic regions (TM/C, red boxes) of cattle (COW), mouse (MUS), rat (RAT), cyno (CYN), and human (HUM). Human FcγRIIa amino acid residues in the ECD involved in IgG-FcγR contact are marked in red and deduced areas of contact are bold and double underlined. Human polymorphism R131H in FcγRIIa and the minipig polymorphism H205Y in FcγRIIa are indicated as arrowhead and diamond, respectively. Above and below the ECD alignment are shorter alignments of the three different versions of the signal region and the TM/C regions, respectively. These alignments are enhanced with sequences from other related FcγRs to demonstrate the homology within each cluster. Note that, in the signal region, some protein sequences are annotated as starting with the methionine indicated by an asterisk. The conserved aspartic acid residue (D) for FcR γ-chain interaction, the ITAM (Y-X-X-L/I) and ITIM (S/I/V/L-X-Y-X-X-I/V/L) motifs are bold and underlined

After translation of the ORF, we compared the newly identified putative porcine FcγRIIa to orthologous FcγRs from different species by multiple sequence alignment (Fig. 3). All human FcγRIIa orthologs share high sequence similarity having a conserved extracellular structure including four cysteine residues required for disulfide bonds to form Ig-like domains (black box in Fig. 3). Human FcγRIIa amino acid residues involved in IgG-FcγR contact (Caaveiro et al. 2015) are marked in red and the deduced areas of IgG contact including residues predicted by other publications are indicated in bold and double underlined (Hulett et al. 1995; Radaev et al. 2001) in Fig. 3. In general, ECD regions involved in the IgG-FcγR interactions showed strong conservation among species, including conserved tryptophan residues, thus indicating that the identified putative porcine FcγRIIa is capable of IgG binding (Fig. 3, black box). Extracellularly, the putative porcine FcγRIIa (aa 46–215) shares 75% similarity to mouse FcγRIII (Uniprot, P08508; aa 31–196), 79% to cattle FcγRIIa (Uniprot, A8DC37; aa 46–215), 80% to cyno (cynomolgus monkey, *Macaca fascicularis*) FcγRIIa (Uniprot, Q8SPW4; aa 30–199), and 79% to human FcγRIIa (Uniprot, P12318; aa 37–206). However, striking differences between the species are observed in the signal region and the TM/C region of the Fc receptors. A closer inspection and comparison to other FcγRs revealed three different non-related signal regions and three different non-related TM/C regions (shown in Fig. 3 as blue and red boxes, respectively). These regions are well conserved between species and combined in different ways with the ECD region of FcγRs (Fig. 3). This suggests a gene “mosaicism” that is very likely the result of duplication and rearrangement of events in the complex *FCGR* locus. We note that this mosaicism implies that the concept of “orthology” should only be applied to the ECD region of the receptors. The intracellular ITAM of human and NHP FcγRIIa is required for direct activation signaling (Isakov 1997) (Fig. 3, red middle box). Mouse FcγRIII, cattle FcγRIIa, and putative porcine FcγRIIa, on the other hand, are lacking such an intracellular ITAM. Like human FcγRIIIa, these receptors signal through associated adaptor proteins including FcR γ-chain (Lux and Nimmerjahn 2013). Charged residues in TM domains are thought to be important for protein-protein interactions in the cell membrane (Cosson et al. 1991). Especially, aspartic acid residues in TM helices are thought to be required for stable surface expression and interaction with the FcR γ-chain (Kim et al. 2003). These residues are also present in the predicted transmembrane domain of the newly identified gene, suggesting that also the putative porcine FcγRIIa signals through the FcR γ-chain (Fig. 3 red upper box).

### A complete picture of the genomic organization of the porcine *FCGR* locus

The new RefSeq assembly contains genes and curated transcripts of *FCGR1A* (gene ID, 613130; transcript ID, NM_001033011.1.1), *FCGR2B* (gene ID, 613131; transcript ID, NM_001033013.2.1), and recently also *FCGR3A* (gene ID, 397684; transcript ID, NM_214391.1.1). The predicted transcript (transcript ID: XM_021089520.1) from the RefSeq gene LOC110260307 (gene ID, 110260307) codes for the 11A 205H polymorphism of putative porcine FcγRIIa. In contrast, the transcript identified from sequences of the Göttingen and the Wuzishan minipig (Fig. 1, Online Resources 3) codes for the 11S 205Y polymorphism of putative porcine FcγRIIa. However, both polymorphic variants were detected by sequencing of one Göttingen minipig.

**Fig. 4:**
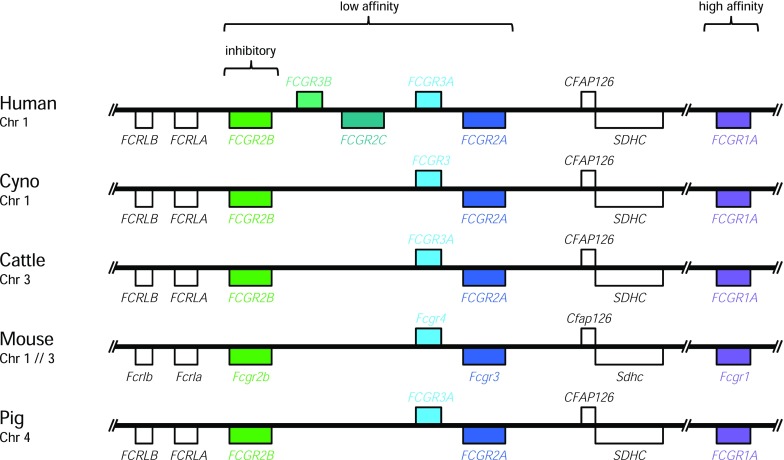
Genomic organization of the *FCGR* locus in human, cyno, cattle, mouse, and pig according to the Ensembl database. The black lines represent a stretch of genomic DNA interrupted by lines indicating a gap of diverse length. All species shown here, except the mouse, carry the gene coding for the low-affinity receptors on the same chromosome. Boxes above and below the black line indicate genes oriented in forward and reverse orientation, respectively. Open boxes represent conserved genes flanking the *FCGR* locus, whereas colored boxes represent various *FCGR* genes found in the species indicated on the left

The gene family of FcγRs displays a similar genomic organization as in most mammals (Fig. 4). Low-affinity FcγRs are organized in one locus flanked by *FCRLB* and *FCRLA* on one side, and *CFAP126* and *SDHC* on the other side. The gene coding for the inhibitory FcγRIIb is highly conserved in mammalian species. *FCGR3A* in humans and pigs is also known as *FCGR3* in macaque and sheep and as *FCGR4* in the mouse (Nimmerjahn and Ravetch 2006). Similarly, *FCGR2A* in humans, NHP, and cattle is referred to as *Fcgr3* in the mouse (Fig. 4). The human genome was found to have species-specific duplications of the low-affinity *FCGR2A* and *FCGR3A* and the high-affinity *FCGR1A* resulting in *FCGR2C* and *FCGR3B* as well as pseudogenes *FCGR1B* and *FCGR1C*, respectively (Machado et al. 2012; Warmerdam et al. 1993). Human and NHP have the gene-encoding high-affinity FcγRIa located distant to the low-affinity *FCGR* locus on chromosome 1. The same organization was found in pig and cattle on chromosomes 4 and 3, respectively. Dogs, mice, and rats, on the other hand, have lost the chromosomal cohesion of FcγRIa and the low-affinity *FCGR* locus. We assume that the ECD region of the newly identified porcine *FCGR* gene is orthologous to human *FCGR2A* and mouse *FCGR3* due to their sequence similarities (Fig. 3) and the orientation within the *FCGR* locus (Fig. 4).

**Fig. 5:**
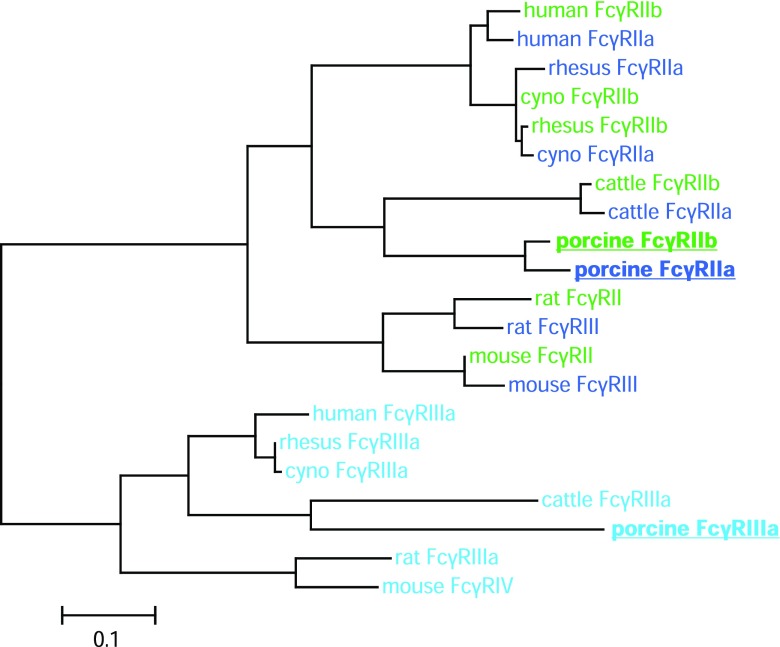
Phylogenetic tree of FcγR proteins in different species. Inhibitory human FcγRIIb and its orthologues are colored in green, whereas low-affinity human FcγRIIa and its orthologues are shown in dark blue. All human FcγRIIIa orthologues are colored in light blue. Porcine FcγRs are displayed in bold and underlined

The phylogenetic tree shows a high intraspecies similarity between ECD region of activating FcγRIIa and inhibitory FcγRIIb including the orthologues in mouse and rat (Fig. 5). FcγRIIIa proteins, including mouse FcγRIV, form a separate group with high interspecies similarity. Full-length porcine FcγRIIa, for example, shows an amino acid sequence similarity of 88% to porcine FcγRIIb (Uniprot, Q461P7), and only 61% to porcine FcγRIIIa (Uniprot, Q28942) whereas the ECD region of porcine FcγRIIa and FcγRIIb are highly similar to each other (95.3%).

### Cellular distribution of FcγRs

**Fig. 6:**
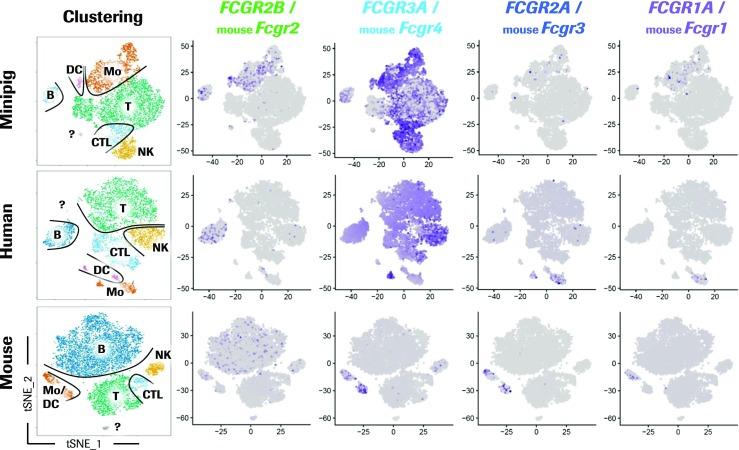
Single-cell RNA sequencing analysis of *FCGR* expression in minipig, human, and mouse PBMCs. For every species, the cells were clustered individually according to their gene expression pattern and displayed as dot plots by dimensionality reduction using t-SNE. The clustering for every species is shown on the left with outlines for better separation. Individual clusters are labeled with “Mo” for monocytes, “DC” for dendritic cells, “NK” for NK cells, “CTL” for cytotoxic T lymphocytes, “T” for T cells, “B” for B cells, and “?” for mixture cell types. In mouse PBMCs, monocytes and dendritic cells are summarized in the “Mo/DC” cluster. The visualization shows the expression of the *FCGR* indicated above where positive cells are labeled in blue and negative cells in gray

Understanding the functional impact of FcγRs requires a thorough characterization of their expression pattern in different cell types. Hereto, the expression of the different FcγRs in minipig PBMCs was addressed by single-cell RNA sequencing in comparison to human and mouse (Fig. 6). This technology was previously used to identify novel immune cell subtypes and monitor responses after immune activation (Jaitin et al. 2014; Villani et al. 2017); however, the cross-species comparison was not performed yet. First, cells of every species were clustered according to their expression profile and displayed by dimensionality reduction on the t-SNE plots (Fig. 6). Then, we identified clusters composed of NK cells, cytotoxic T lymphocytes, T cells, and B cells in all species by their characteristic expression profiles (Online Resource 2). Such an approach enables to enumerate the expression levels of any gene of interest in all cell types in an antibody-independent manner. It was striking to see that minipigs have a considerably larger part of PBMCs assigned to the monocytic lineage. At the same time, the number of B cells identified in minipig PBMCs is smaller than in humans and significantly smaller than in mouse PBMCs. Subsequently, the mRNA expression of the different FcγRs was then analyzed in every species (Fig. 6).

The activating low-affinity FcγRIIIa is most strongly expressed among the FcγRs in all the species studied here. Minipig PBMCs revealed a strong and relatively homogeneous FcγRIIIa expression on all monocytes, DCs, NK cells, and cytotoxic T lymphocytes. Interestingly, T cells and B cells showed heterogeneous expression suggesting either different cell subsets or activation states. Human monocytes are often separated in classical, intermediate, and non-classical monocytes according to the CD14 and CD16 (FcγRIIIa) expression (Ziegler-Heitbrock 2015). As expected, the larger CD14^high^ classical monocyte subset did not express FcγRIIIa, whereas the minor non-classical CD14^low^ subset was strongly positive for FcγRIIIa. Also, in mice, it is the cluster containing the monocytes that shows expression for FcγRIIIa, while the other immune cell types, in contrast to the other species, show no expression. The inhibitory low-affinity FcγRIIb was found to be expressed mainly on monocytes, B cells, and DCs of the minipig. Human monocytes were not found to express FcγRIIb, while mouse FcγRII was weaker expressed in the monocyte and DC cluster as compared to the minipig. Expression of FcγRIIb in human and mouse PBMCs was mainly found in B cells. FcγRIIa, the activating low-affinity receptor we identified with our mapping strategy, is expressed at lowest levels in minipigs and humans. In the minipig, FcγRIIa mRNA was only detected in very few cells of the monocyte cluster. More monocytes were positive in the human and expression levels are slightly higher. Mouse FcγRIII, the orthologue of FcγRIIa, is expressed on most cells of the monocyte/DC cluster at highest levels compared to the other species. Similar expression levels and patterns were observed for FcγRIa. In the minipig, the expression is at low levels and restricted to monocytes. In humans, CD14^high^ CD16- classical monocytes express FcγRIa, in contrast to CD14^low^ CD16+ non-classical monocytes. Mice show a similar FcγRI expression pattern on a subset of the monocyte/DC cluster.

**Fig. 7:**
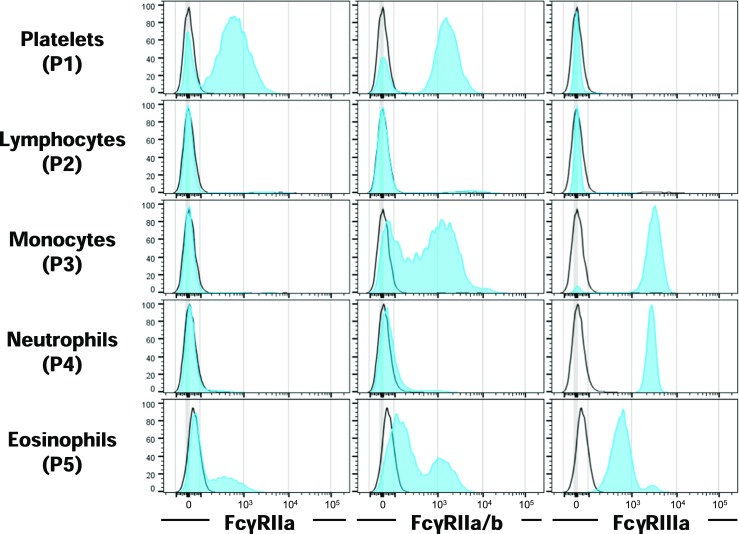
Flow cytometry analysis of FcγR distribution on minipig blood leukocytes. Gating strategy is shown in Online Resource 4. Histograms normalized to mode show stainings observed using an FcγRIIa-specific HuCAL antibody, an FcγRIIa/b cross-reactive HuCAL antibody, and an anti-CD16 (FcγRIIIa) antibody in blue. Stainings with a HuCAL control antibody are shown as an overlay with a black line representing the background. A representative analysis of one out of three experiments with different Göttingnen minipigs is shown

As gene expression studies only measure the mRNA, which may not fully reflect surface protein expression, we performed flow cytometry to assess the FcγR expression in the blood of three Göttingen minipigs. Cell types were identified according to the forward and side scatter properties, and their identity was confirmed using specific antibodies (Online Resource 4). Figure 7 shows a strong staining with the FcγRIIa-specific HuCAL antibody on platelets (P1) and a weak staining on a subpopulation of eosinophils (P5). The FcγRIIa/b cross-reactive HuCAL antibody stains platelets, most monocytes (P3), and some eosinophils as well. FcγRIIIa staining was observed with varying intensities on monocytes, neutrophils, and eosinophils. Only a few cells were positive in lymphocyte population (P2).

## Discussion

The three different classes of FcγRs form a finely tuned system required for efficient immune reactions in mammals. Minipigs represent a valuable alternative to NHP in preclinical studies. Thus, it is of particular importance to know all FcγR components in a preclinical animal model intended for testing of therapeutic antibodies. The characterization of the low-affinity FcγR proteins and genes in minipigs should provide a basis for preclinical studies with therapeutic antibodies.

While the inhibitory receptor is widely described as FcγRIIb (in the mouse known as FcγRII), the nomenclature of the low-affinity-activating FcγRs has evolved in a far more divergent manner. The low-affinity FcγRIIa is well-known in humans and has been described in the NHP, cattle, and other mammals, such as rabbits and sheep (Akula et al. 2014). The orthologue in the mouse, however, was named FcγRIII at its discovery (Nimmerjahn and Ravetch 2006). This receptor was initially not known in pigs due to an incomplete genome characterization and therefore was not described by Akula et al. (2014). In the present study, we were able to identify the putative porcine FcγRIIa located on chromosome 4 of the Göttingen minipig. The orthologue to human FcγRIIIa is known in NHPs, cattle, and other mammals, including the mouse, where it was designated as FcγRIV (Nimmerjahn et al. 2005). The orthologous FcγRIIIa cDNA and protein were also described in the pig but the corresponding gene and its genomic localization was unknown (Akula et al. 2014; Halloran et al. 1994). Here, we describe the localization of the gene *FCGR3A* encoding the minipig FcγRIIIa between *FCGR2B* and the putative *FCGR2A* on chromosome 4 of the Göttingen minipig on the forward strand. The identification of the putative *FCGR2A* and the localization of *FCGR3A* in pigs allow the comparison of the low-affinity *FCGR* locus to other species. We found that this locus of the minipig is organized similarly as in NHP, cattle, rat, and mouse with the position of the putative porcine *FCGR2A* gene coinciding with the other species. Nevertheless, significant differences to the human *FCGR* locus were observed. Thus, the complete characterization of the low-affinity *FCGR* locus of the minipig presented here confirms the absence of genes coding for homologs of the human FcγRIIIb and FcγRIIc, as is the case for all other animal species studied so far.

Sequence similarity displayed in the phylogenetic tree in Fig. 5 shows that FcγRIIa and FcγRIIb of the same species usually cluster together, probably originating from a duplication event early in speciation (Akula et al. 2014). The high similarity of the ECD region of porcine FcγRIIb to the newly identified porcine FcγRIIa fits in the pattern observed with the corresponding receptors from other species. Therefore, we suggest naming the transcript FcγRIIa. However, as detailed in Fig. 3, exons coding for the signal region and the TM/C region of the FcγRs appear to be shuffled during gene duplications and rearrangements leading to a mosaic structure that is characteristic for primates, rodents, and artiodactyls, respectively. Predictions suggest intracellular signaling by porcine FcγRIIa via interaction with the FcR γ-chain as it is described for cattle FcγRIIa and mouse FcγRIII (Lux and Nimmerjahn 2013). This similarity strengthens the hypothesis of the orthology among these receptors. On the other hand, FcγRIIa in primates is known to signal via integrated intracellular ITAM. It should be considered that differences in ITAMs potentially lead to functional differences between Fc receptors (Herik et al. 1995).

Two potential polymorphisms, A11S and H205Y, were identified in the main FcγRIIa transcript. The first located in the signal region and the latter was identified in the Ig2-like part of the ECD involved in the interaction with IgG antibodies (Fig. 3). Due to its location, the H205Y polymorphism could potentially influence binding affinities to certain IgG subclasses. Apart from that, we found three potential isoforms of porcine FcγRIIa with unknown functions and significance, probably generated by alternative splicing. Similar splice variants were already described for porcine FcγRIIb (Xia et al. 2012; Xia et al. 2011) and FcγRIIIa (Jie et al. 2009). In particular, humans were shown to have splice variants and polymorphisms with significant functional consequences. Altered binding affinities are associated with the outcome of therapeutic antibody treatments and with disease progression (Bournazos et al. 2010; Ziakas et al. 2016). Studies with more minipigs are required in order to assess the potential incidence of polymorphisms, splice variants, and sub-isoforms. Additionally, their biological relevance remains to be assessed.

Biological responses triggered by FcγRs do not only depend on the affinity of IgG interaction but also on their cellular distribution (Albanesi and Daeron 2012). Knowing the expression of FcγRs on immune cells facilitates the estimation of effects triggered by IgG interaction. We performed single-cell RNA sequencing on minipig, human, and mouse PBMCs to study the FcγR expression profile on various cell types.

In the Göttingen minipig, FcγRIa transcripts were only identified in monocytes at similar levels as observed in human and mouse. Like in humans, no FcγRIa expression was detected in minipig blood DCs although FcγRIa expression was often reported in human DCs (Nimmerjahn et al. 2015; Tamoutounour et al. 2012). FcγRIa expression, however, was usually analyzed in tissue resident or induced DCs and not found in blood DCs (Langlet et al. 2012). Devriendt et al. (2013) showed that the FcγRIa expression profile on porcine DCs depends on the activation stimulus, and similar findings were observed for human DCs. Therefore, FcγRIa expression can neither be excluded from minipig blood DCs nor from tissue-resident subsets. Varying expression levels of FcγRIa between minipig and human DCs could, however, result in varying capacity for antigen presentation by immune complexes and cytokine production (Cohen-Solal et al. 2004; van der Poel et al. 2011).

Only a few monocytes of the minipig showed weak staining for FcγRIIa. Generally, the FcγRIIa expression in PBMCs seems to be lower in the minipig as compared to humans and mice. This low expression was also observed in porcine gene expression data from NCBI (Li et al. 2017). Low expression of FcγRIIa in monocytes could theoretically be upregulated upon inflammatory stimuli similar to other activating Fc receptors (Nimmerjahn et al. 2005; Pricop et al. 2001). Like humans, minipigs express FcγRIIa on platelets as detected by flow cytometry (Rosenfeld et al. 1985). Platelets are mediators of immune responses upon binding of IgG immune complexes via FcγRIIa. This interaction can lead to platelet activation, phagocytosis, and ultimately to thrombus formation with pathological consequences (Worth et al. 2006; Zhi et al. 2015). The minipig might thus be a good model to study platelet-mediated functions and side effects of therapeutic antibodies, such as bevacizumab-induced retinal vein thrombosis, in contrast to mice that do not express FcγRIIa on platelets (Meyer et al. 2009). Gene expression data from NCBI Gene show that FcγRIIa is mainly expressed in the liver and the lung of pigs. Generally, the porcine FcγR expression is mainly detected in the liver, lung, and spleen tissue. This expression profile suggests that FcγRIIa mediates important immune functions in tissue-resident cells other than platelets in the blood.

Single-cell RNA sequencing of minipig PBMCs shows FcγRIIb expression on B cells, DCs, and monocytes. FcγRIIb expression on monocytes correlated with flow cytometry data using FcγRIIa specific and FcγRIIa/b cross-reactive HuCAL antibodies. Presently, the exact cellular distribution of FcγRIIb cannot be evaluated due to the lack of specific antibodies. A previous study postulates cross-reactivity of anti-human CD32 antibody (AT10) without showing data (Balmelli et al. 2005), a finding that could not be confirmed in our hands (not shown). The expression of FcγRIIb on minipig B cells and DCs reflects the situation in humans. On the other hand, minipig and mouse blood monocytes were found to express FcγRIIb as well, whereas human blood monocytes do not (Nimmerjahn et al. 2015). Low levels of FcγRIIa together with high levels of FcγRIIb on minipig monocytes could result in enhanced inhibitory signaling compared to humans. Hence, this could lead to an underestimation of effects or toxicity observed in minipig studies with therapeutic antibodies with FcγR-mediated effector functions.

Porcine FcγRIIIa was so far the best studied Fc receptor due to its high expression and the availability of specific antibodies. Its expression pattern was closely reflected in our single-cell RNA sequencing and flow cytometry analysis (Piriou-Guzylack and Salmon 2008). Minipig and human FcγRIIIa was found to be the highest expressed FcγR in PBMCs. In both species, T cells and B cells were found to express FcγRIIIa mRNA. Whereas FcγRIIIa expression on human T cells is controversially discussed in the literature (Nimmerjahn and Ravetch 2008), it can be excluded on B cells. Therefore, the FcγRIIIa expression in T cells and B cells of both species is considered as unspecific or represents different subsets or activation states. The difference between minipig and human is that FcγRIIIa is only expressed on monocyte subpopulations in humans, whereas it is expressed in all monocytes in the pig (Rubic-Schneider et al. 2016). The ubiquitous expression of activating FcγRIIIa on minipig monocytes could possibly counteract the inhibitory effects of FcγRIIb and the low levels of FcγRIIa. In therapeutic antibody research, a careful evaluation of the interaction to the various FcγRs would be needed to estimate the activation or inhibition potential of the antibody on minipig monocytes. Altogether, the human expression pattern of these FcγRs is more concordant with porcine than with murine monocytes (Fairbairn et al. 2013). The expression pattern of FcγRs is known to vary not only between species but also between individuals. As mentioned before, it can also be influenced by different stimuli, the immune status, or upon treatment. Therefore, further studies with more minipigs under different conditions are required to make a precise statement about the FcγR distribution in health and disease.

Our work allowed the localization of FcγRIIIa and the identification of the hitherto undescribed FcγRIIa on chromosome 4 of the Göttingen minipig. The newly identified FcγRIIa described here is considered as an orthologue to human, NHP, and cattle FcγRIIa as well as to mouse FcγRIII due to the highly conserved extracellular structures. The identification of FcγRIIa completes the picture of FcγRs in the pig and provides the genetic foundation for further studies. Our expression studies are the first to describe the expression of FcγRIa in monocytes and FcγRIIa on platelets of the Göttingen minipig. Additionally, FcγRIIb was found in monocytes, DCs, and B cells. The higher expression of FcγRIIIa and FcγRIIb and the lacking expression of FcγRIIa on monocytes are different to humans. Therefore, effects on monocytes should be carefully evaluated before using the minipig in preclinical studies with therapeutic antibodies. Nevertheless, FcγRIIa expression on platelets makes the minipig a valuable model to study platelet-mediated effects of therapeutic antibodies which are hard to evaluate in mice.

## Electronic supplementary material

Online Resource 1 List of primers used for amplification of *FCGR* sequences and the identification of the putative porcine *FCGR2A* transcript. Refer to Fig. 1 for an overview of the primer location. (PDF 294 kb)

Online Resource 2 List of differentially expressed genes used to summarize clusters to the indicated cell types. In the mouse, it was not possible to separate monocytes and dendritic cells. (PDF 323 kb)

Online Resource 3 Nucleotide sequence of the low-affinity *FCGR* locus of the minipig including *FCGR3A* in forward orientation and *FCGR2B* and *FCGR2A* in reverse orientation. Exon sequences from *FCGR2B*, *FCGR3A*, and *FCG2A* are highlighted in green, light blue, and dark blue, respectively. Adjacent 5′ and 3′ untranslated regions are marked in gray. Splice acceptor (AG or CT) and donor (GT or AC) sites are bold and underlined. Start and stop codons are marked with an open box. (PDF 254 kb)

Online Resource 4 Gating strategy for flow cytometry analysis of minipig blood. Whole blood from Göttingen minipigs was stained with the indicated fluorochrome-labeled antibodies. From single and live cells, gates P1-P5 were selected using forward (FSC) and side scatter (SSC), and cell types were identified using the following antibody clones: CD45 (K252.1E4), CD61 (JM2E5), CD3e (BB23-8E6-8C8), CD21 (BB6-11C9.6), CD335 (VIV-KM1), CD8a (76–2-11), CD172a (74–22-15A), CD14 (MIL2), and CD52 (11/305/44). Numbers indicate the percentage of cells within the respective population (P1-P5). (PDF 196 kb)
