# Supplementary material for: The genomic organization and expression pattern of the low-affinity Fc gamma receptors (FcγR) in the Göttingen minipig
Source: Immunogenetics. 2018 Dec 18;71(2):123–36. doi: 10.1007/s00251-018-01099-1 (PMC6327001; doi:10.1007/s00251-018-01099-1)
Supplement: Supplementary file 1 — List of primers used for amplification of FCGR sequences and the identification of the putative porcine FCGR2A transcript. Refer to Fig. 1 for an overview of the primer location. (PDF 294 kb) [file 251_2018_1099_MOESM1_ESM.pdf]

Primer	Region	Orientation	Sequence
**JE2**	*FCGR2A* Ig2	forward	CCAGCCTCTCCATCCCACATGCAAACC
**JE4**	*FCGR2A* TM/Cyt	reverse	GCAAAAAGGAGCCCCATCGCCAGGTAG
**JE5**	*FCGR2A* 3'UTR	reverse	GGCCCAAGTTGCTGTTAAGTCGGGGCTG
**JE24**	*FCGR3* Ig2	forward	CTTCGGAGGCTGTGAAAGTC
**JE26**	*FCGR3* TM	reverse	TGATGGGATAGGTGATGGAC
**JE28**	*FCGR2A* Ig2	forward	ACCCCTAGCCTGGTGTTCC
**JE35**	*FCGR2A* 5'UTR	forward	TGCGTACTCCAGGAGGTGATGG
**JE36**	*FCGR2A* 5'UTR	forward	TGCTATTCCTGGCTCCTGTTCC
**JE41**	*FCGR2A* intron	forward	GGTCAGTCTCTTGGGTCAGC
**JE42**	*FCGR2A* intron	reverse	CCACCTAAGATGTGGTCCCAG
**JE47**	*FCGR2A* intron	forward	GGGCTCAATGACTGTTTGCTG
**JE49**	*FCGR2A* intron	reverse	CTGATCCTCCAGGGCAGTATCC
**JE58**	*FCGR2A* intron	forward	TCCAGGGGCCTTCTTATACTC
**JE61**	*FCGR2A* intron	reverse	AGCCCTCGGATGTATGAAAAG
**JE62**	*FCGR2A* intron	forward	TTGCTGGCCTGTTAGTACCTG
**JE64**	*FCGR2A* intron	reverse	GAGGAGCCTACGTTTGGAATC
**UPM**	5' or 3' RACE		CTAATACGACTCACTATAGGGCAAGCAGTGGTATCAACGCAGAGT
**UPM-short**	nested primer		CTAATACGACTCACTATAGGGC
