# Supplementary material for: The genomic organization and expression pattern of the low-affinity Fc gamma receptors (FcγR) in the Göttingen minipig
Source: Immunogenetics. 2018 Dec 18;71(2):123–36. doi: 10.1007/s00251-018-01099-1 (PMC6327001; doi:10.1007/s00251-018-01099-1)
Supplement: Supplementary file 2 — List of differentially expressed genes used to summarize clusters to the indicated cell types. In the mouse, it was not possible to separate monocytes and dendritic cells. (PDF 323 kb) [file 251_2018_1099_MOESM2_ESM.pdf]

	Minipig	Human	Mouse
Monocytes	*SIRPA*	*CD14*	*Cd14*
	CD14	*CD16*	*Cd68*
	CD163	*CX3CR1*	*Adgre1*
	SLA-DRB1	*ITGAM*	*Lgals3*
	SLA-DRA	*CD163*	*Apoe*
	FCN1	*CD68*	*Mafb*
	LGMN	*CD86*	*Fcgr3*
	TREM1	*CSF1R*	*Ly6e*
	CLEC4E	*CCR2*	*H2-Aa*
	CLEC7A	*SELL*	*H2-Eb1*
	CCR2		
	CYP1B1		

	Minipig	Human	Mouse
DC	*SIRPA*	*ITGAX*	*Itgax*
	CD14	*PLAC8*	*Thbd*
	FLT3	*FCER1A*	*Cd38*
	ITGAX	*IL3RA*	*Cd209a*
	PLAC8	*CD1C*	*Cd74*
	FCER1A	*CD33*	*Flt3*
	CD74	*CD1E*	*H2-Aa*
	SLA-DRB1	*HLA-DRB1*	*H2-Eb1*
	SLA-DRA	*CLEC4C*	*Ifi30*
	CD33	*NRP1*	*Napsa*
	IFI30	*LY75*	*Itgb7*
	ITGB7	*ANPEP*	*Syngr2*
	SYNGR2		*Clec10a*
			Ahr
			Tlr13
			CD24a

	Minipig	Human	Mouse
B cells	*CD79A*	*MS4A1*	*Cd79a*
	CD19	*CD19*	*Cd19*
	MS4A1	*CD79A*	*Cr2*
	SLA-DRB1	*CD40*	*Fcer2a*
	SLA-DRA	*CD86*	*CD22*
	CD86	*HLA-DRB1*	*Ms4a1*
	CD40		*CD86*
			H2-Ab1
			Cd24a
			Cd38
			CD40

	Minipig	Human	Mouse
**T cells**	*CD3*	*CD3E*	*Cd3e*
	CD4	*CD4*	*Cd3d*
	CD8	*CD5*	*CD40*
	ITGB1	*IL2RA*	*CD8a*
	CD5	*CCR7*	*CD8b1*
	FOXP3	*MAL*	*CD5*
	IL2RA		*Ms4a4b*
	CCR7		*Cd28*
	Cd28		*Il7r*
	Il7r		*Lef1*
	Lef1		*Dapl1*
	Dapl1		

	Minipig	Human	Mouse
**Cytotoxic T cells**	*CD3E*	*CD3D*	*Cd3e*
	CD8A	*CD8A*	*Cd8a*
	GZMK	*CD8B*	*Gzmk*
	GZMB	*GZMK*	*Gzmm*
	GZMA	*GZMH*	*Ccl5*
	GNLY	*GZMA*	*S100a6*
	CCL5	*CCL5*	*Lgals1*
	Klrk1		*Klrk1*
	Lgals1		*Crtam*
	Crtam		*Eomes*
	Eomes		
	GZMH		
	Gzmm		
	S100a6		

	Minipig	Human	Mouse
**NK cells**	*KLRB1*	*NCR1*	*Ncr1*
	NCR1	*NCAM1*	*Klrb1c*
	PRF1	*NCR3*	*Klrd1*
	GZMA	*KLRC1*	*Klrc1*
	GZMB	*KLRD1*	*Klrk1*
	GNLY	*B3GAT1*	*Itga2*
	KLRD1	*NKG7*	*Gzma*
	NKG7	*KLRB1*	*Gzmb*
	CCL5	*CCL5*	*Prf1*
	KLRK1	*GNLY*	
		GZMB	
