# Supplementary material for: The genomic organization and expression pattern of the low-affinity Fc gamma receptors (FcγR) in the Göttingen minipig
Source: Immunogenetics. 2018 Dec 18;71(2):123–36. doi: 10.1007/s00251-018-01099-1 (PMC6327001; doi:10.1007/s00251-018-01099-1)
Supplement: Supplementary file 3 — Nucleotide sequence of the low-affinity FCGR locus of the minipig including FCGR3A in forward orientation and FCGR2B and FCGR2A in reverse orientation. Exon sequences from FCGR2B, FCGR3A, and FCG2A are highlighted in green, light blue, and dark blue, respectively. Adjacent 5′ and 3′ untranslated regions are marked in gray. Splice acceptor (AG or CT) and donor (GT or AC) sites are bold and underlined. Start and stop codons are marked with an open box. (PDF 254 kb) [file 251_2018_1099_MOESM3_ESM.pdf]

>mpig_FCGR_locus
CCTTGAGTTAAGAAGAAAATAAAACGAAAGTTGTAGATCATTTAGGGACA
AATAGCAATGACAAAAATACTTATCAAAATTGAATGGATGTGGACAAAAA
TAGCAATAAGAGGGAAAGTTATAGCCTTTGTAAATTTGTTTAAAAACAGA
AGCATAAGAATAAATGAATAATACAGTAAATCTAAATAAAGTACAATAAA
GAAATAATACATTAGAGGAGTTCCCGTCGTGGCACAGTGGTTAACGAATC
CAACTAGGAACCATGCGGTTGCAGGTTCGATCCCTGGCCTTGCTCAGTGG
GTTAAGGATCCGGCGTTGCCGTGAGCTGTGGTGTAGGTCGCAGACGAGGC
TCGGATCCAGCATTGCTGTGGCTCTGGCGTAGGCCGGCGGCTGCAGCTCT
GATTGGACCCTTAGCCTGGGAACCTCCATATGCCGCAGGAGCAGCCCAAG
AAATGGCAAAAAAAAAAAGACCAAAAAAAAAAAATAATAATAATAATACA
TTAGAAAATAAACATGTCTTGGTTCATTAAGTCAAAAGTTGTGACCAAAA
AAAAAAAAAAAAAAACCCACAGACAAAACTTTGGCAAGTCCCTAAGATAA
ACATATATAATAGTCAAGATGGTTTACTTTCCAAAAGTAACTCCACCTCA
AAATGATTAAGCTAAAGTAAGTTATTCCCCTTTCTAGTGATTGATTTAGG
AATGGGCATGTGACCTAGTCTTGACCAATATGATGGGACAGGAATACTTC
TGGAGAAAGTTTTCTCATTCTTAAGAGAGAGAGAGAGAGACACACATAGA
AGAAGTAGTTAGTCCCCTTCTCTATATCTTTGGGAGTGATGCCTAGAAAT
GCTATAATCATCATGCAGCCAGGCTAAGGGCAACACTCAGAGGATTACAG
AAAAGTGGATCCAGAGTCCTGATGTACTGGAGACATCCTGTCTCTGAACT
TTTTGTTATAGGAGATAATGGATTTCCTTATTGTTTAAGCCAATTCAAAT
CAGGTTTCCCGTTATTTCTATCTGAAGTCATTCTGATAAAAGAAAGCAAG
CCTGCATATGGGGGCAAGAGAAAAGTATAGATATATAAATTATGACAAAA
AAAAATAGAAGAAAATACAATGTATATAGCTGCATAGCCGTAAAATGGAT
AATTTTCTAGGAAAGTAACATGTTTTTCAAAACTAATTCCAAAGATATAG
AAAAACCTGATCAATAATGACAGGAAAAAAATTTGTCAAAAATCTATAGT
AAAAAAGCTTTCAAGCCCATGTGACACTATAGGAGAATTCTATCAAATCT
AAATTAATTAAGAAGAAGCTGAAGGATCCTAAAAACTATTAGAAACAGGG
CAGATATAGGGATATAGGAAGCTGGTCTCATCTGGGCAATGTCATTGAGA
TGAACGAACTTCGCTTGTTTTCAGTCGTAGTGTTTCTGCTCAAGATTTAA
ATTCCAGGGAAATGCTGTTGATTGGCCCAGTTTGAAGTGCATGTTCTCAT
CTAGAATCAAGAGATGAAGTCAACCTACCTACTCACTCGGACTGAAAGGG
GAAGTAGAGCTATTTAGTCAAAGAGAAAACAGGTTCTGATACTAGAATGA
GGGGAAATTCATGCTGGGTAGGGAAATAATCCATTATAATCAGGTTGGAA
AAAATTTTAAATAGTCAACACTGAGAAGGGGGTCAGGAAATGGATACCCT
AATATACTCTGGGAGGACATGCAATCATTTTTTTTAAGAGCTGCACCTGC
AGCCTATGGAAGCCTCTGGGCTAGAGGTCAAATTGAAGCTATAGCTGAGA
CCTATGCACAGTCATGGCAACACCAGATCTGAGCCACATCTTCGGACTAC
ACTGCAGCTTGTTGCAATGCAGGATCCTTAACCCACTGATTAAGGCCCAG
GATCTAATCCATATCCTACAGACACTATGTAGGATTCTTACCCTGCTGAG
CCTCAATGGGAATTCCTATAGGTGATCTTAATTATATCTTTTTCAGATTA
TTGATCTGATCAGGTTTTTCTATATCTTTTCTTTCTTTTTTGCTTATCTG
TTTTTTTAAATTCGTGGAATAAAATAATTTACATCTAAATAAAACAGTTG
CTACTGTGTAATTTATAAAGGGATTTGTTACTATAAAAATGGGCATGAGT
ATGTGAATAAGAAAATCACAAAAGAAGAACCATGAATGACTAGCACTTTT
TTTTTTTGGCTGCCCTGGTGGCATGCAGAAGTTCTTGGGCCAGGGATTGA
AACTGTGCCACAGCAGGCACAACTCCCAATCCTTAACCCACTGAGCCACC
AGAGTACTCTAATTCTAAAGCACATAGTTTGACATAAAGTAAGCCCTCAA
TAACCCAGTAATTGCAATTACTATTCCCTATTTTTATAACAATATGTGGG
TATGAAATCTGATATGTGAATATTATAGAAAGCTATAGAAACTACCCAAC
AAAGGAAGCTGCTAAATCCTTAATGCAAAAGTGTTGCACAATGAAGCTGT
GTCAGAGTCCCAGGAAAATGAGGCTTCTCCAAGGGCACCCACCAAACTCC
CAAGGGAGCTATTAGTCATATTTCTAACCTGCTGGGATGGCTTCGTTGAG
AAGGTCACATCTTTCTGGTAACTAATAGAAGGGCAGGGCAGGAAGAGTCA
GCAGTCTCCACTGGGGACTCATCTGAAGCTTGGCTTCCTGAAAACTGGTG
TTGCAAAGTGATTGTTTATTTGCCACGGAAGGAAGAGGAAGAGCACAAAT
TCACTGAGAGAAGGGTATTTGGTTTTAAGAAGAGGGACACTTGTCCTACA
TTCTAAATCTTTCCTTTCCTCTACCTCCTACCCATTACTACCTTCCCAAG
ATTCTTTTATCTTTTCTTTTCTTTTTTTTTTCCCATTCTTTTGTCTTTTTA
GGGCTGCACCTGTGGCATATGGAAGTTCCTGGGCTAGAGGTCTAACTGGA
GCTGCAGCTGCTGACCTATGCCACAGTCTCAGCAACAGGAGGTTGGGGGT

GGGGGAAGATGGAACCATATCTGCGACCTACACTGCAACTTGCAGCAACA
CTGGGTCCGTAACCCACTGAGCAAGGCCAGGAATTTAACCCTCATCCTCA
TGGATTCTAGTTGGGTTCTTAACCCACTGAGCCCCAATGGGAATTCCCCA
AGATTCTTACCATTGAAAACAAATGCATCGGATCAGTCTATGTCTCCAAT
TAATACAGCCTTTAAAAGGTAAAGAGAATGTTGTTTGTGTAGTATTTTAT
TATGAAAGTAATATATGATCTACACAGAAAAATAAAGGAAATGAAAAGCG
CCAATAATTCCATCACCCTGAAACAATTGCTTTTAATATATTGGCATATG
TTAGTCTGTTTCAACATTTCTGAAATCATGTATATTTATATAATTTTTCT
CATGTCATCTGTAACATAAGTATTTCCCGTGTTGCTACAATCTCTTATTA
AAAGTAATTTTCACTGGCTGTGTGGAAGTCTAACAGAAGAGTCCTAATGG
TGAGAAGTGCTGAGGCACAGAAGAAATCAGCAGGTCAGTTATCTGAGAAG
GTATTGTCTGTGGCTTTCAGAAAAGGGAGCTTCAGGATTCATGTAGATAA
CTCTGGAGGGGTCGTTTTCCTTTGCAACTGCGAAATTCTCTTCTCCTCCC
TAAGAGGAATTCAGGGCAGAAAACACCAAATCCTTATTCTCTTCCCCAAG
TCCCAGGCAAGATAATGAAATTTAAATGTGGTTCTGGTAATCTGAAGCCT
CTGCTTCTCCTTCTGCAGCTTCCGGATGCAAGAGAAGTGAATAGGTGATT
GTATCCTCAGC**CT**AGAGAAAGCAGAAAGGAGTAAGGGGGATCCATAGAGA
GGTCACAGGAATGGAGGCAACAAAAGAGGAGGCAGGGAGGGGTAGGAAGG
AGAAGAAAAAGCAGGAGGCTGGCATGGCTC**AC**CTCAATTTTGGCAGCCTC
TTCAGCATCAGTGTGATTGG**CT**GTGGGCAAAAAAACAGTGATCAGCCAGG
GCACTGCTATGGAATAGGGCCTACCCATGCTGCTGCTGAGACCCAAACCA
GGCTTGAGGCAAGGACCACTGACTCCAACTCAGGCCTGTAGGCCAAGAGC
TCCATTTCCCACCCACCCTCAGTATCCTAGACATGGCCTCTCCCACTGGC
CACACTGATAAAAGGAAGTAGTCACTGGTGTTGCATTCTTTTTTTCAGAG
AATCACTGAGTTTTCCTCAGAGCAAACCAGGCAGCTTTAGGGATCTGAGA
TCTTGGGGAAGAGATGGGGGGTGTTCAGTATTAGGGTGAAGTAATCCAGA
TTTTTGCCAGCCATAGTTGTGGCCTTTCCATATTAGCCAGATGTAATATA
TCTTTGTTGGAATTTTGAAGACCCTGATACTCACTGACCTCAAGAAGAAA
AAAAGCATGAGAATGAGAGTGAGCAAGGGAGGCTGGAAGGAAGGAGAGAA
GGAAGCAAGCAAGCCAGCCAGCGAATATTCTCCCTGGTTAACACCTAAAG
CACATCCAAATTTCCTCATGTTTTTGCCAATAAACTCACTTTGCCAGTGC
AAAAAGCAGCCTCAGTATCTGCAAAGCAGCATTCCTTAAGAAGTGGGGAA
GGAGTCTGAGACAATGCATCTAGTGTCTTCATTCATCTCCAGGACATAAG
GAGTTTCTGTGTCCCACGCTCCACATTCTGGAACAAACCATGCCCTGAAT
TCTCCTTCTAATGTATCCCTCAACAATCCCAGCAAACTAGTTGTGCTTTT
TGAGAGACCATCAATCCCAGTGCCTTCTTGTGAAATAAATGCCTGGTGAA
ATACATTTATGCCAGGTAGAACCAAACATCAAAGGTGTTAGTCCACTTGT
GTTTGGATTTGTTTCTACATTGGTTTTGTCCTGGTGCAAACTCAGGTCCA
CATCCCCACTTGGGCTGGCCAAGGGACATAGGGACTTTGCGCTAAGCGCA
GCTAGGCAGCTGGGCCATTTTGCTCAAGCTCTCAAAGCTGTTGGCTCTGT
TCTGAGTCATACTCCAGCCATCAGAGGTGGCGTCCTCAAAGGCTTCTTTT
TCCTGTCCATTTCTGCTGTCTGGGAGCTTCCAGAGAAAGCTCCAGGTTTC
CTTATGCTCTGGCTTTACCTACAGTCAGGAAATTGCCTGGAATACTCCTG
TTCCTTTAACATACTCCCAGCTGGGCCAGGAGCTGGATGGTTACTGACCC
TCAGAATGTCAGACCTGAAAGGACTCAGAGGTCCTCCAGGCCAACCTACT
CATTTGCCCCCGAGAGGTGACTAAACCTCAGGATGAGGAATGACACTAAG
GATTCCATTATACAATGGTGACAGGGCTAGTGCCAGGCCTGCATGTAATA
AACCTACACAAGTGGGCATTAGAGCCAAGCTTTGCCCACAGAGTAAAGAC
CACTAATGCACCTAGTACCCCAGCACTTTCTTAAGCCAAAAATCTGCCCT
TAATGTTCCACTAAGAGCTGAGCTCGGTTACCCTTTCCCTGTCACTCCAC
AGAGGGACACTCTGCTGCAAATCTAGGGTTTATTCCTTTGGCCTCCCTCT
CCTTTCCTCCTAGAATCTTATCCCCTCAATTCTATTTGCCTCAGTCTTAC
AATTTCTAACCCCTGTCCTGTGCCAGCCCTTCCAAGTTTCCATGGAAAGA
CCTAAATATCTCTGATGTCCTGCTCTGGAAATATCCAGCTGGTCTAACCC
CAGAAACTCGGACAAGGCCCTCCCCTCTCTATCTTGGACAGGTGTTCATA
TCTTCTCCCCATCTCCAGTGAGGTCCTCCTTAAGCCCAGGAGTTAGTCTC
AGAACTGACCAAGAGATTTGAGCCCCTGCCCTCCTCCCACTGCCCCCCTC
CCTCTCCTGTGCTCCCAGCAGAACTCACACAAGCCACTTCTGATGCACAA
GGTGGGGCGTCACTGAGCCCCCAGAGGAGGAGCTGCACTCACCTGGTTCC
TCAGGGAGGGTATCTCCCTTTTCCCTGTGCTTAGGGGTTCCTGGGAGAGC
TAGGAGGGGCACATAGGAGGTTAGTAGGATTATGGGATAGGACCTGAGTC

TTCAGGACCACGGACCAGAGATAAGACAAAGTTAGAGCCAACACAGACCC
TTCTCCCACACAGACATCTCTCCTCCCCATTCCCCAGGGACTGCCTTAGC
CTGTAGAAGGTCTAGGGAGATATAAGTTGGCTAAGCTAACAGNNNNNNNN
NNNNNNNNGCTGGTCTAGGGAGATATAAGTTGGCTAAGCTAACAGTAGGG
ACTGCTGGAACGATTAGAAAAGCCATTGTTCTGCCTTTTTTTTTTCTTTTC
TTCTTTTTTTTTTTTTTTTTNTTTTTTTTTGTCTTTCTAGGGCTGCACCTGC
GGCATATAGAGTTTCCCAGGCAAGGGTTTGAATCAGAGCTGTAGCTGCTG
GCATTCGCCACAGCCACGGCAATGCCAGATCCAAGCCTCACCTGCACACT
ACACCACAGCTCACAGCAATCCCAAATCCTCACTGAGTGAGGCCAGGGAT
AGAACCTGTGTCCTCATGGATGCTAGTCAGATTCATTTCCACTGAGCCAC
AACAGGAACTCTGCTCTGCCTTTTATTTATTTATTTATTTATTTATTTAT
TTATTTATTGGTCTTTTTACCATTTCTTGGGCCGCTCCTGCAGCACATGG
AGGTTCCCAGGCTAGGGGTCGAATCAGAGCTGTAGCTGCCAGCCTACACC
AGAGCCACAGCAATGCAGGATCCAAGCCGCGTCTTTAACCCACTGAGCAA
GGGCAGGGACCGAACCCGCAACCTCATGGTTCCTAGTCGGATTCATTAAC
CACTGCGCCAAGATGGGAACTCCTGCTCTGCCTTTTAAAATCACCTTTCT
GAAAATCATAGACTTGGAGAAAAGCCTTGTGGCTACCTGATGGGAGGCGG
AGGGAGTGGGAGGGATCGGGAGCTTGGGCTTATCAGACACAACTTAGAAT
AGATTTACAAGGAGATCCTGCTGAATAGCATTGAGAACTTTGTCTAGATA
CTCATGTTGCAACAGATCAAAGGGTGGGGAAAAAATGTAATTGTAATGTA
TACATGTAAGGATAACTTAATCCCCTTGCTGTACAGTGGGAAAATAAAAA
AAATAAATTAAATTTAAAAAAATTAAAAAAAAATAAAATCACCTTTCTCC
CTCTCAGTATCTCTCCCTGTTTCTCTACATGTATTTATTATCAGTAACTG
AGGTCTTGGCTATGAACCATTTGCAAAGTGGTTTGTGGTTTGCTGTGTGC
TAGGTACTGTTATAAGAGCTTCAGATTCGTTTATACTCAACCCTATGATG
TGGGTTCTATTTGTGTCCTCATTAATCAGATGAAGAAATGAGGCACAGAG
AAGTTGAATGTGATGAGAAAAACTTTTGCTTTCCTGAAAAAAAACAACTT
TGTTTCTCTGGGTTGCTGCCTAGGCATTTTACAGTGTTGATAACCCTGCT
CACACCCAGAGTTTGGACATTTAAGATAAGGACCTGAGGAGTTCCCGTCT
TGGTGCAGCAGGGGCGGATCTGACTAGGAACCATGAGGTTGCAGGTTTGA
TCCCTGGGTTAGGGATCTAGCGTTGCCGTGGGCTGTGGTGTAGGTGGCAG
ACGAGGCTCAGATCTGGCGTTGCTGTGGCTCTGGTGTAGGCCAGCAGCTA
TAGCTCCGATTAGACCCTTAACCTGGCAATCTCTATGTGCCACGGGTGCA
GCCCTAAAAAAAAAAAAAAAAAAAAGATAAGAACCTGAGTTTAGGATTCCC
ACCATAAGTTCTCACTCCGCTTCTCCTGGGGGCACCTTTTAAAATAATGC
CTTTAGAGTTAAGCCCACAAAAAAGTTAATGTCCTCCACCCAATCGCCTT
ATAAGAAATAGTTGCTTGCTGTCCTGCTGGCTCTTCCCTGCTCACCCATG
ACCTGGGACAGAGGACTGCCACCCCCAACCCTCCTCATTTTTGGGATCTG
TAAGTAACAAATCTTGTGACACTACTTCCTTTGTATGAGGGTATTGAAAT
TGTGCCTTCAATCAAAATGACCCCAGGGTTTTCACTTCCCCAATCTGGGG
ACTCAGCTGCTAAGGGAGTTGCTGACCCCATTTGGGTGATTTATGCCTCC
TCGTTTATCACATCGCAAACTGCATGTTCTTAATTCACCAGCTCCATCTT
GTCAACACATTGAGTAACTCACCCAAGGTTATTCAGTTGATGACCTGAGG
AGCTGAGATTTGAACTCAGGTAGTCTGCCGCACCAGGTTGCTTCATCACT
ACACATCATTGCCTCCCTATGAAGCCTTCTCTAACTGCTCCAGTCCATTT
TGGGTTATGCAGGCTCTTCTGTACCCAGTCATACCTTTTGCCTTCCCTAA
CACTGGTCTTATCCCCTCTTATTATCATGATCTATTTGTACTTCAGCCTT
CTGCACAAAATTATGAACTCCCTGAGGGCAGAGACAGAACTTCTTTATCT
TGGCATCATCCACAGAGTCTATTGCAGTGCTTGATACATAAGCAAGTTTG
TTCTGGCCTTCTCAAATGAAAGGTTGAATGAATAAAATATGGACTATAT
CTGAGTGCTAAGGATTCTCTAGCACAAAGACAGCAGTTCTAGCAATGTGT
GGGGTTAGGGCCTTGGCTAAGTAGGACCTGATAATGAGGGACCAAGAGGA
GCCACAA**AC**CTGAAATCCGCTTTCGCCTGAGGCGGAACCAGGCTGCTATG
GCAGCAGCAATAGCCATTGCAACAATCCAAGCAACCACAGCCACAACTAT
CGTCACCAGCAAGTTGTTCAAGCTGGACC**CT**GGGAAGACAGAACTCAGGA
CAGGCTGTACTCACCCTGCCCCACCTCAACCTCCCTGCCCTCCTCCAATG
CTATACTTGGAGCTGTTTTCTCTTTGGGTCACTCACAGGTACTGGACTCA
CTTTCTCCCCCAACCTCACCTGCACCGGATTTCTGTGACTGGCCTTCTTC
CTCCCCATCCTCAGCTTTCTCTGCTAATCAAATGAAGGTACACTGTGTAA
GGCAGAGGGAAGTACTCCTCTTAGCCCTCGGGAATAGAACGGAAGGTGGA
CCTGAAGCCTCTGCCACTGTATCCAGCACAGCCTTAAACTGTCAGGGACA

TGCCATGCCTTTCAGAGCGGAATGGACATAGCATGAGACATAAGAGGAAA
TTAGGAAAACCTCAGCTCTTAGGAATGTCCAAGAGCTCAGGGAACCTCTG
CACCAATGAGAGAGCTCTGTTCTGCTGCTGTGCATATTATGATGGGGCCT
ATATTTCAACAACCTTGTCACCTATCACCTGGAAGCTAAGGACTCTGAGC
TTTAGCCACTACTTAGTAGGCACCCATATCCCTGCCCTCTGCCCCAGTGC
CCAGGTTTGCTGTTACGGCATTTCTCAGATCTCCATGTAGGTCCGTATGG
CCTTATCCTTAAATGTCCATCCCTTCTACTCTCCTCCTTTCATCTTGACA
GAGTTCCC**AC**CTTGGACAGTGATGTTCACAGACTGTGATGAGTACGACAT
CTTCCCGATAAATCCTGTGCAGTGGTACTCGCCACTATGACTCTGGTTTG
CTTGTGGGATGGAGAAGTTGGAATCCACATAGGAAAACTTCTTAGATTTT
CCATTCTGGAAAAATGCAACCTTATGCAGAGGCTTGTTTCTCCAGCTATG
GCACCTCAGCAAGATGGGCTCCCCTTCCTGGAAAACCAGACTAGAGGTTT
GGAGCAGCAGCCAGT**CT**GAAAGACACAAAGAGACCACAGGATCCGGGAGG
TCTGACTCAATACTGAGACCCAGACCATCCTTACTGGTGGGATGTTAGGT
GTGGAGGGGAAAGCTTAAGTTTGGAATCAGATTGCCCTGGTTTCTAATTC
TGGCCCCATCATTTATGAGCTCTGTAATTGTGAGCAAGTAATGTTAACTC
TTCCAGGCCTCAGTTTCCTCTTATGTGAAAATGGTGACAATCACATCATT
ACCTCTCTAGAATTTGCAGGTGCTCAGGATATGCTTGTCATCAGGTTCCT
GTCCTGGAAGAAGCCAGCTGCAGCCCTGTCACGCCTGACAGGGCTCTGTA
GGAAAGATGCAGGAGACTCTGGGGTACAATCCCTTCTCTGAAGTGGGGGG
GGGGTCCAGTCTGTCATGGGAGATAGCAGGTGAGGGGGATACAGCAGAGA
TATATGTCTTTGGGGAAAGTCATGAACATCCAACAAATATTAATTTGGTG
CATATTAGTAAATGTGGAATTTCATTTCAATAGGGTCTTTGTCTAAAGGC
AGCCATGTCATGGGTGGGCCATTAGCTTTCTGTGTACTTTCTTCCCCTCA
ACTTTCATCCGTTCACTGAGGAAAACATATATTCAATTTCTTATTCCTCT
TCCCCTTCTTCATCATCATCATCATCTTCTTCTTCAGGCTGTGCCCACAG
CATGCTGAAGTTCCTGGGCCAAGGATCAAACTCACACCACAGCAGTGACC
TGAGCCACAGTAGGGGCAATGCCAGATTCTTACCCACTAGGCTATGAGGG
AACGCCTATATTTAATTTTTTTGTTGTATAGGGTATAGAGGCTAAGGTGG
GTACTTGTAATCCTGACCTCAGCCCACAYACTCCTTTTTCCTAAAGCTAT
AGGACTGAGCAGAGTTAGAATTGGAAGGAAAGTGAAGAAAACAATGGATA
CATGGAAGAGAACCAGGAGATGAGATAAAAATGACAAAAGCCACATGTGT
GCCAGCCACATGTGTTTGGCTACAAACATACTAGGTCCCTGAAGCAAATC
AGAACAATGCCAACAAAAGTCTCTACACAGATGAGGGAATAAGTGACAAC
AAAGAGAAAGCTCTGGGCCACAGCTACAGTGGGACAATCCAGAGGAAAGC
TGGGGGACACCAGATCTTGCTACTTGTGTCACAGSAGCTAGAAAACCCCA
CTTCTCCCACTGGGAAGGAACCCCTCTGTCCCAGCCATTTCCTTCCCTAW
CATCCTGTGWATTCTTTCTCTTGGAAAACTCAGACCCTTGCAGGACATGC
CACACCAGGATTAGGTGAGCAGTCCTGGTTCTCTAGAATGCCCCTCTTAT
AAAGCTTGAGTTAGGGTAGAGGTAGGGAAGGCTAAGAATTGTAAAAAAAA
AAAAAAATTTAATGAAGTGGAACCCAGAGAAAATGTCAAAGCTGCTTGCA
TGTAGCACCAGCAACCAATTCTTGCACTTCCCCTCCCTGCTTGGCTAAAA
TCCCCTCTACAGAAGGTGCACCTCATTAGAACAGCATGTTCTCCCTAGCA
GTCTCAACTGGTATGAGCTTATAACCTGGACAGTGGGGAGTGTCTGCCTA
AGGCTGGTTTGTTCAAGAGGGCCAACTTACGCCTCCTTGAGGACAATTTC
TTGGTGCATGTCTTGAGGGCAGGGCCCAGTTTCATGTCCATGGTACTCAT
GAGATGTTCTTTAAGAAATAAAAGGGCTTTTGCATCAATYTCYTGGGAAG
AGAACTCCTCTGCAATCTTCTGGAAGTCTTGAAGAGAGGCGATAAACTCA
TACAATCTTCTACACTCCACTGGCTAAGTTACTGGACAAGAACACAGGGT
TGATGCTGTGAATCCTGGGGTAGGCAGAACTGTGTCAAAGTTCCCTGGGT
CACACTCCCCCTGACCAGCTCTCACTTATAAAGACCCAGGGACATTGGAG
AGAGTGCTTCATCCTAAGTGGAATTCTCTGAACCCTCGCTAGAGTCCTCT
TACCTTCRGAGCCACTTGCCCTGGATCTTAGCATGGGTRATGTCAGAGGA
GCTGCAGTAGGGTCCACACTGGTGAACACAAGCATTATGGACTTCTCAAG
ACACTTCCATGTGTTTTCCTCTTCAGGCAGAACTGAGGCTACAGCTCACA
CTATACCTTTTTTTTTTTTTTTTTTTCTTTTTCATTTTTTGCCACCCCGT
GGCATATGGAGTTCCCGGGCCAGGGATCAGATCCGAGTCTCATTTGTGAC
CCAAGCCGCAGCTCTGGCAATGCTGGATCCTAAACCCCCTGTGCTGGGCC
GGGGATCAACCTGCTTCCCAGGGCTCCAAAGACAGGGCCAGTCAAGCTCC
TATACTCTACCTCTTAGTGCAACTCATGGAGTAGAAACTCTTGGAGCCAC
AGAACTGTTCTGCAGGAGTGCACKTGCCACAAGGCTGGAACTTCAGGAGA

TTTGTCCTCTTGACTACTCAGCAGRTGGGCTGTCCCCTCCCCAGGGGCCA
CCTGACTGATTCTCATTCAGCCTTGTCGGGAGGCCAGTCTACACAGAGCA
GCTTCTCAGACTTTTGAGTAACAAACAACCCACTGGGAAAGGTTCTGCTC
CATCCTGGATTCCAAAGCCCTCGATCATGTGGCTGAGCATGTGGGGCTTC
ACCATGGAGGTTTGAGTCACCCATTTGTCTGGACACCATGGCTAGTGTAG
GAGGTGGTGCTGTGGGCAGGGGCCATGGCTGCCAGTTCCCCCYTTGGTGA
ATTAGYATTCCCTCTGGTCACTGGTTCAGCTTCTTCTCCAAACTGCTCTC
CTTCTCCTYGGTGGCCTTGGAGCTCACTGCTAGTGCAGAACCCTTGGCAG
GAACCCTGAACCCAAGGTGAAGACATCATCTCCTTCCTCCTTGCATTTGA
CAGCCAATGTCTGGGTTTGCACAGCAGGTACACAGACTGCATGTAGAAGA
CAACAGGGACCCAGACCTGTTGGCTGAGGGGCAGTGAGAGTCGTGGCTGC
TGCTGCTGCTGCTGCTGCTACTGCTGCACCCACTGGAGGTGCTGTGGGGA
GAAGTACCGGGATTGACAGTGCTGGAGCTGTCACCAGMGGTCGATAGCAA
TCTGCTGCCTGGTCATCACCTGTTTCTCCAAGAGCTGGATCCGTTGCTAT
TGCTAAATTGGTGAATGGGGCTGGATCTGTGTGTGGGTGACTGAGCTAGT
AAGGGTCTGCCTGGGGGCAGGTGTAGCTCTCTATGTCAGGTTCATGCCCA
CATCCTGCTGACCACTCCCATCTGCTTCTGTCTTCTTGGCCACTGCACTT
TCTGCCTCTGTCCAGCYRCCCTGGATCACCATCAGTGTTTGAAATGCAGG
CAAGGGCTCCACCACATCCGCCTTTCCTGGGACAGCTYCCACCACCACCC
ATTCCAGAGGAGGAACTGACTCAAGGCCCATTTCTACCTGGACMCATGGA
CCTCAGAATGCTATTCCCACTGCCTCCACCAAATGRACTAATGCTAAAGA
CTGGCATGATCCCTATAKAGGAAACCTGAGCCACAGCCAGATGTCTGGGG
AGGCTATAGACAGGGGAGGCTCAACGGGAGAACAGTCTTCTGGTTGGAGA
CTTGGGTTGAGGCCTGTTGGTTCCTCACTGCCCAGTGCTGCTCCTGATCT
GCATCTACACGGACTCCAGGAGCCTGAGCASCTGGCACCTCCAGCCGGAC
TACAGTCACTGTCCTAATAGGCATCAGGATGAGTTGAGAAGCTAAAGGCA
CATTCCAGCCCATGGTCTGGTTTACTTGGAAAAGGTTCCCCAGCTGTAGT
CTTAGGTACATCTGGGCCTGGAACAGGTTGAGGGGAGGAGGAGGTGGTGT
TTCCCAGGAGCAGAGCTTGGGTCAAAGTGGTAACATWGGGAGAGTTCACA
CTCGAGGAYTGGCTGGTCAGTTGGGTGGCTGATGTTGTGGCCAGATTGAC
TGAGGCCTGGGTGTCGGTCAGCTGCTGTGTAGTGCTGGTGTTTGGGGAGC
TGGCCTGCTGACTAGCAGCACTTGTGATCAGCTGCACAGAGGCCAGGCTA
TGCAGCTGGGTCCGCTGAGCAGCAGGTGCACATGAACTGGTAGAAAGACG
GGGGCCCATAGAGCTATCACAGCAATGCCTGCAGAGCCTGTTCTGCTTGC
TGTTCATACAGTGGCTTTGACTATTGGGGGCCGAGGGCTACCACCTGAGT
TGGGACTCTCATTGGTGCAACTGGCATTCTGTTGGCTCTAATCTCCACTA
GTGACCCAGGGTCCCCCAGGCTATTGCTCTGGCTCAAGACTTTCACATCG
AGCTCTGCACTCAGATCTTCCAGGGCAGTGTTCAACTCACATGCTGGGGT
CCCCTATTGCCAGGCAAGGTGGCTGGGGGTAGGGGTGATCATACAGTCAT
GAGGGGTGGAATTCCCTCATCTCCACCCCATTCCTGGAGGGTCGGGGCAC
TGAGGTCAGGGCTCTGCCAGGCCTCTGGGGATTCCCTCCAGGACCATCTT
TCCCTGCTAAGCCCAGAGGGGAAAGATGATAGGTCTGGGGCMGAGGCCCA
TTTGGCCTATTAGCTATCTTTGAGGAAAATTTAGGAGCCAGGTAAGAGAA
GGGGACAAAGTGAAGTRTAACAATGACAATATTCATCATAATTGCATTAY
GCCTTTTCTTTATGGAGAACCTACTTCTTGCCAAGATCTTTGGAGAATCT
TTTTTTTTTTTTTTTGTCTTTTTGCCATTTCTTGGGCCGCTTTCCTGCG
GCATATGGGGATTCCCAGGCTAGGGGTTGAATTGGAGCTGTAGCCGCCGG
CCTACACCACAGCCACAGCAACGCGGGATCCGAGCCGCGTCTGCAACCTA
CACCACAGCTCACGGGCAACGCTGGATCCTTAACCCACTGAGCAAGGCCA
GGGATCGAACCTGCAACCTCATGGTTCCTAGTCAGATTCGTTAACCACTG
AGCCACGATGGGAACTCCCAAGGKCTTTGGAGAATCATTGATCTGTTCAG
CAAACAGTCATTGAGCCCTGCCCTGTGCCTGAGGCTGGAAATACAAATAA
GATTAAAACACCACTTTCACCTCAAGTTGCACAGAGAGCCRGGGTTGGGG
GTGKGGCAGGGAAATGCTGATTCTCACCAAGACCTGATAGAGGAGGCAGT
AGTCTCTCCATTGAGCAGATGAAGAAACTGTGGCTCAAAGCTAAATGGAA
TCATCATGTTTACAYGGCTAGTAAGTTCCAGGGCTAGGATTCAGTTGTGG
TCTGTCTGTGCATTTGACTCTGCTGTAAAAATGGAAGCGGTCCTATGAGT
TAGGGATTTCCTTCTGCCTCTCTCACACTCAGCTGCCTGTTTATGGCCTT
CTTACCTTCTTGTTCAAGTCAGGACCGAGGCAAAATTGCCCAAATACTGA
GAAATCTCTTAGGCAGTTCCACCCTACCRCACATACTAAACCAAGGTGGW
GGGTGGGAAGGGAAATGAGTGGGCAAAAATGACTTCAGATTCATAGCCAT

GCTTCTCAGTAAACAGGCCACCCTCTTCCCTAAAAACCTCTGCCCTGTAA
GCTGATTTCATCTCTCCTTGTCCTCCCAKACTCTCCCAGGCCTTCTTCCA
CTA**AC**CAGAAATCACATCCAGATGCACAGGGTCGCTGAGACTGGAGTAGG
CAGTCTGGCACCTGTAGTATCCGCTGCTAGCTCTCCTGGCCTTAAAGCTG
AAGCTGGGCTGGTTCTCGGTCCAGGTGAAGTTCCCATTATGGAACCACTG
GGTGGTGGTGTTCCCAGGGTCATGGTCGCCCTGGCACGTCAGTGTCACAG
AATCCTCCTGGAGCACATTGATCCATGCAGGCTGAAGCTTCACCTCAGCC
TTTGGGAGAACTG**CT**AAGGGGCAGAGAGAAGAGGCAGCATGAGGAAAGGG
GTCCCCTAGGGCCCAAGTAGACCCCAAATGTTGGATAATGGGAAACTGTG
GCCCTGCTGGAGACATCAGGAGAGGAACCCAACCAGAACTGTTGTGCAAC
TGAAGGCAATYCCTCCAACTCATATAGCCCACCCAGGGGCCCCAAGAAGC
CAAGTTCTCCCAGGGAGCTTGTCTGCTCCTCAGGCAGAWTAAGAAGGAAG
GGAATAGGAGAGGGGCAAGGCAGACACATTACAAATACAAGTACACCTGA
CCAAAGGATGTCTAACCGGTTCCTTCTTTCTCTCCATCTCTCTGTCTCAT
CTCTCTCTCTCTGTTCCTCTCTTTCTCTCTCACACACATGCACTAYACAT
ACATCTTTTTGGGGAATCAATTTCTCTCCTATAAATTGAGGTATTTGGAC
GTTAGTAGATAAATGCTCTCCACAGTTGGATGAAGACAAGTGGGGACATC
TGGTGGAGATGCCAGGCTGAGAGTCAGATGTCATGTTTCTGTTCTACCCT
CTTCTACTCCTTTACCTTAGGATAGACCTGTCCTGACACCCAAGCACAAA
AGGGTCTCCGTGGTCTCAGTAGAATGAGAAAGGGGGAGAAAGACTGGAAA
GCCAGTCCAGGGAAATCCCGACTTCAGTGCCGCAGGGAGAGCCCCATCCC
CCTAACTAGTCCTGGGAAGTAGAGAGTTTAGTGGTGCAGAGAGAGAGGTG
CTCAGCAGAGTTCACACAACCACACTGCAACCAATGGTATCTCCTCCTGT
TAATGCCCAGGGATATAGGCCGTAAAGCCCTCAGATATATGAAAAGTACA
GGATTTATCAAAACATGAGTATAACCTTAGCATGATTTTGYATGCAGCTA
CAATTAATTTTTTTTTTAAGTCTTTTTGCCTTTTCTAGGGCTGCTCCCGC
AGCACATGGAGGTTCCCAGGCCAGAGGTCGAATCGGAGCTGTAGTCACCG
GCCTACGCCAGAGCCACAGCCACACAGGATCTGAGCCGCGTCTGCAACCT
GTATCACAGCTCCCTGCAACGCCAGATCCTTAACCCACTGAGCAAGGCCA
GGGATGGAACCCGCAACCTCATGGTTTCTAGTCAGATTCGTTAACCACTG
CACCACGACGGGAACTCCAGCTACTACTAAATTTGCCTGGGCTTGTGAAT
TAGTATGGAGAGAGGAAGGCGATTGAAGTAGAAAGCAGTTCTTTTCTGTG
ACAGTATCTGTGAGGTCTCCTGGTTAACAGAACCAGAAGGCTGAAGCACC
TGTACACAGTACTGCCCTTCCCCCACTTCCCTGGGAGGAAGGAGAAAGGG
ACAAGGCATCTTGGGGGAGCAATTACTCTTTCCTGGCTCTGCCTAGGTCT
GGGGTTGCTGCTGCATKCAGACAGAGCATCTCCAGACAGGGTGCTGGGAA
CAGAGCAAGGGGGTAGCTGTGGGGAAAGAGGATGTGGGTGGAGCACTT**AC**
CAYGTGTCCCAGGAACAGGAG**CT**GTAAGAGAGAAAGCAAAGATCAGCATG
GTGTTTGGATTTGTTGATTCATGGTTATAAATAATCACCTCCTTGCCTTT
CTCCTTAATTCTCCTGTCCTTCTCTGGCTGCAATTCATTCACCTGTGTTG
TCTGTCACCTTTACTTCTTCAGCTATGTGTTAATGAAATTTCCACATAGA
GGCTGGATTCATATCTGTAAATTTACTGTCTCCATATTCAAAAGGACACT
AAACTGCCATTCTGATGCTTTTCTCTAGTAAATTTGCTCTGAGGCTCTTG
GTAGTGACAATTGGTTTTTTCCTCATACCTCAAACTTTCCCATCTTCTGC
CTTCAGTCCCCTCTCATTCAAAGCTAATGTCCTCCTTTATCCTCACAGAG
AAAACAAAAGCAGTTAGACAGAAGGTCTCACGTCTTCCCCTAACTAAACC
CTCTATCCTATTTGTGTCTCTACCCATCTTCCTTTCTTGGTTTTCACTGG
GATAATATCTTTCCTTCTATCTGTGGTCAGGGTCTATCCTGTGTCACCTT
CCCTTTCACCCTCTCAAGGATTTCCCCCTCTCGGTTATTTCCTTTTGCTT
CTGCCTCACCCATTTCTCCTTCTTAGCTGGATCTTTCCTACCAAAATAAT
TATATTCCAGGATCTTTCATTAAAGGGAAAAAAAACACACACTTGGTCCC
ACATACAATTCCAAAATTATTTCTCTGCTCCCTTTCCCAGCCTGATTTCT
CAGTTCTTTTTCTACATAGGCTGTCCTGATTCCTTCTCCTTCCAATCACT
CTTTCATTCTCTGCAATTTGAACTCTGCCCTTACCAGTCCACTAAAATTT
TTCTTGTCAAAATCGCCAATGACTTTTCTGTTGTCAAATCAAGTGGACAT
AACCTATCACCTCTCCCAGGTTCCCCGATAAAAGTGTATATCTCCACCAT
CTACCCAGGTAATCATGCCTGAAAACTGCAAGTATTTTTATTCCTCTCTC
TCTCCCAAACTCCTTCTGCCTTTGCATCAATAAGTTGTGTTCTGCTTCCA
ATTAGTTCTTGAATGCATCAACTTCACTCCACATTACTATTTTATTTTAT
TTTATTTTATCCTATTTTAATGGCCACGCCCACTACATATGGAAGTTCCC
AGGCCAGGGATCAAATCCAGCTATTCCAATCCTCAACTCACTATGCCAGG

CCTAGATTGAATCCACACTACCACAGAGGTAATGCCAGATCCTTAACCTA
CCAGATCCTGTACCACAGCAGGAACTGTCATACTGCTTTGTCACCACAAG
CTTTCTTCCATCTCCTAATTTGTCTTTTCCCTTCTTTTGTTCACCCTGCA
ATTCATTCCCCATGGAGAAGCTAGCAGGTTTTGTTTGTTTGTTTGTCTTT
TTGTCTTTCTAGGGCCAGACTCGCAGCACATGGAGGTTCCCANNNNNNNN
ATCTAATTGGAGCTGTAGCTACCGGCCTACAGCCATAGCCACAGCAATGN
NNNNNNNNNNNNNNNNNNNNNNNNNNNNNNNNNNNNNNNGGCAACACCAG
ATCCTTAACCCGCTGATGGAGGCCNNNNNNNNNNNNNNNNNNNNNNNNNN
NNNNNNNNNNNNTCGTTTCCGCTGTGCCATGAGGGGAACTCCTAGAGTTA
TTTTTAAAATAACAAATCAGGAGTTCCCATTATGGCTNNNNNNNNNNNNN
NNNNNCTAGTATCCATGAGGATGTGGGTTTGATCCTTGGCCCTGCTCAGT
GGATAAAGGATCCAGTGTTGCCATGAGCTGTGGTATAGGTCACAGACACA
CCTCAGATTCTGCCTTGCTGTGGGCCAGCAGCTGTGGCTCTGATTTGACC
CCTAGCCCAGGAACTTCCATATGCCATACATGCAGCTGGCCCTAAAAAGA
AAAAATAANNNNNNNNNNNNNNNNNNNNATAAAATTTTTAAAAATCAGAT
AATATCATATCCCTGCATACAAGCCTTCAATACTCTTCCTTTGCTTTTAA
AATAAAATGTAAATATTTACCAGGCCACACCTGATAGGACCCTTGCTCAC
CTCTCCAGCCTCATCTCGAACCCCACTTTCCCTTCTCATTTAGCCCCAAT
AACACCAGCCCTCTTGAAATTCTTCAAACTTGGGGCTTTTCTGCGTGCTG
ATCTCTCTACTCCAAACCTCCTTCCTTTGTATCTTCAAATCACTTAATCC
TTATCATCTGAGTCACTTCCTCAGAAAATTCTTTCCATATCATCCTGTTT
GTGTAGGCGGTTCTCTCATCTCCTCCACAACAACTGTTCTTATCAGAATA
TCCTGTTTACATCTTCGGAGCCATTTTCCAACTTATATTTATATTTATTT
TTGTGTTGCTTATATCCCTCAGTAGAAGGTAAGCTCAAAGAGGGCAATAG
CCGTGTTAATTTTATTTGCCAGCGCTCTGGAAATCATTCATTGAGTCCAT
CCAGGTGGGAGATAGGAGAGCCCAAAGTTGTATTGATAATAGAAAGTGAA
GACGAGGAGTTCCCTTTGTGGATCAGAGAGTAACGAACCTGACTAGGATC
CACGAGAATGTAGATTCGATCCCTGACCTCACTCAGTGGGTTAAGGATCT
GGCATTGCTGTGAGCTGTGGTGTAGGTCGAAGACGTGGCTCGGATCCCAC
GTTGCTGTGGCTGTGGCTGTGGTCGGCAGTTGTAGCTCCAATTCGACCCC
TATCCTGAGAACCTCCATATGCTGCGAGTGTGGCCCTAAAAAGCAAAAAA
AAAAAGAGAGAGAGAGAGAGAAGAAAAAAGAAAGAAAGTGAAGATGAGAT
AATGGAAAAAAATAAAAATCATTTNNAAAAAGTGAAAATGNNNNNNNNNN
NNNNNNNNNNNNNNNNNNNNNNNNNNNNNNNNNNNNNNNNNNNNNNNNNN
NNNNNNNNNNNNNNNNNNNNNNNNNNNNNNNNNNNNNNNNNNNNNNNNNN
NNNNNNNNNNNNNNNNNNNNNNNNNNNNNNNNNNNNNNNNNNNNNNNNNN
NNNNNNNNNNNNNNNNNNNNNNNNNNNNNNNNNNNNNNNNNNNNNNNNNN
NNNNNNNNNNNNNNNNNNNNNNNNNNNNNNNNNNNNNNNNNNNNNNNNAA
AAAAAAAGTGAAAATGACATGGCATGAGAGAAATTTAAGAGGCAGAGAGA
ATGTGGCACTTGATGAGATGTGGGGATGATATAAACAGCTGACTAGGAAA
AAGGTTGATTGTGGTCACACAAATGTAAATGTAGGAAGGACAAGAACATT
TTCGAGGGAAAGATGACAGTTAGATTATAAATACATTGTGTTCCTGAGGC
GACAAAGCAATTCGGAAGTTTCCAACCAACAGGTAGAAATATGAAATTGT
ACTTGACACAAATAAGAAAGATGGGTTTGGAAATTACTGACATAGAAGTC
ATAGCTAAAAACCAGAATGTGTAGGATTGACAGCATGACAGTGCGGGGAA
CGGGTAAAGTAGAGACCAAGAGGAGAAGGAAGAAAATAGTTAAGAGTCCA
GAAAAGTAAAGTCAACTTCCATTCATTCCTTCCACATTCAAGAAATATTT
ATGAATATTAAACAGCTTTACACTCAGTGTACTCACTGTGAGGGAGACAA
AGACCGTGTGCTCTAGGTTAGGAAGGCTGTCTCTCCTGTTTACAGATAAA
TCCCCAAGTCCTAGAACTGAGCCTGCACATGGCATTTATTTGATGTTTGA
ACAAGGAAGTTTGTGGAATCTTTGAAAAGCAGAGAGTTCAGAGTATATAC
TGCCTGGCTCTAAAGGAAATCCACTCGGAAGAGTAGAATTGGAGCTTTAT
TAAGCACTGGAGACACTGAAGGTATGTATGGAGGGCAGGGACTCCTCCAG
AGCTGTAGTTGAGGAAGTCATGACTTCAGGGTGCAGCATGGATTCAGGCA
AAAGGACACTAGGAGCAATCAAGAGTGGATCAGGAGGGAGTTCCCATTGT
GGCACAGTGGTTAATGAATCTGACTAGGAACCATGAGGTTGCAGGTTTGA
TCCCTGGCCTTGCTCAGTGGGTTAAGGATCTGGCATTGCCGTGAGTTGTG
GTGTAGGTTGCAGACGCAGCTCAGATCCCATGTTGCTGTGGCTCTGGCAC
AGGCCNNNNNNNNCAGCTCTGATTCGACCCCTAGCCTGGGAACCTCCATA
TGCTGCAGGAGCAGCCCTAGAAAAGGCAAAAAGACTGAAAAAAAAAAAAA
AAAAAAGAGTGGATCAGGAGGTACTGTGAAAATTGAGTTACTGAGCAATG

AAGACCAAAACAAGGCGAGGTGCGGCAGATAAGGATCAAAAAGATGGAGT
AAATCCTTGCTGACTTAGCAAAATGGCAAATGGAGGAAAATACCAAAGCC
TCTCCCAGGAGTCTTGTCTAGGCATCACACTGGTATCACCCAGGCAGAGG
TAGGGAAGCATACCACCTCTCCAAGCCTGAGCTTCTTAAAAAGGCTTTGC
TGGCTTTCTCCTTGATCAGTGACAGGACCTATCCCCATATCTGGGCAGTA
CAAGGAACATGCCTGCTTCTCTGGTCCCACAGGTGATGATCCCAGAATTG
CCTGGTGTTTATTCTTGAGCCCCATTCCMTGGATAACCRAGACYCTCTTA
TGCTTTTTCCCAGGGGAATCTCCTTTCACTTCCGCTGCTTGAAGTGGTKT
CAGGGGCACAGAGAATGGCCACTGCACCCCACCACAGTGTCTTATTGCTT
CCCTCCCCCTCCTATCTGCTTAAAAGATGATTAGCCCTCCCAAGATTTGG
TCCCAGTAAGACATAAATTTCAAAGGAAGGTAAACACCTTGGCCCCTCAT
TAGCCTTTCAAGTCTATGACACTAGACTTTCCTCAGAGCTGGCTCTGCTG
TGCAGGGTGGCAGAGCACCAAGGACATGTGAACCCAGTGAGATTGGGCAA
AGACTTAAAAGAGATTGGCAAAAAAACTTCCTGATTCTGCCAATTTCACA
ATTTGCCAAAGCCAGCAGGGTGGTCAGAATTTGATTTCCATCAGCCTGCC
CTCACCCTCCTGACTTTCCTGACGTGCTTCCCATGCCCCCCAACTCCTCA
ACTTTGCCCAAGCTCAGCCCCAACCRAGGTGGCAGCAGCTGAGTTAGAAA
AGCACACCTGCCTCTGCTCCTGCTCCACCGTCCCTCCTGGGGACTCTCAC
TC**AC**CCAGGAATAGCAGAGCTGTCCACAGGAGCATGTGGCCCAAAGGATG
GCAGGGCATGCAATCAGCCCAGTCACTCCTGGCAGMGGGGAGGGCTAGGA
ACGAGGGGATCCCCATCACCTCCTGGAGTACGCAGGTTGCCTCTGGCAGC
GCCAGCGCAAGACAGGCTTCTCTCAAGGCTCGACAGATTCCACCCCAGAG
GACAGACAAATTCTGGAGAGCAAAAGAGGAAGTGAAAAGAAATGTTTTCT
CATTAGCTCCTCCCCTCCACCCTCTTTCATCTCAACTGTCTCCTCCCAGA
AGATTCAGGGCTTCCCAGAATGGATGTTCCTCAGTCCCACTCCAAATTCA
CAGAAAATGTAGAAATTGGGAAACAACTTCTCTTCCTCTGGGGCCTCAGC
TGGGCTCAGCCTCTGGGAGAAAGAGGGGACTAGTTACYCAAACTCAGCTC
ACAACCTCCTGTCTSTCTCTGTCTCTTTAGAAMAGGCGATGGAGGAGAGT
GASCCCCCAGYCTTTCCGTACTTCTAGATTCATTCAGAGGCCTAGACACC
TGCATCAGATTCCTTCTGAGGCTGAGCTTGGCGGCTTGAGACCAACCACC
ATCATCATTTGATTTTCTTTATTATCATAATCCTGACTCTGTTTTGGATT
GTGGAACAAGGACAAAGAGGTGTGTCAGTGTGACAGTAGGATGGTCACAC
TAACCCTCTTTATTCCCTTTGTCATTCATCCTTTTACGCATTCCTCTGAC
ACATCTCTCTCTCTCTCTCTCTTTTTTTTTTTTTTTTAAGCCGCCCCTGCA
GCAAATGGAAGTTCCCAGGCCAGGGATCGAAATGGAGCTGCAGATGCAAC
CTATGCCAGAGCCACAGTAACACCAGATCTGAGCTGCCCTGGTAAACTAC
GCTGCAGCTTGCAGCAATGCCAGATCCTCAACCCACTGAGCAAGGCCAGG
GATCAAACCTTCATCCTTACAGAGACAACATCAGGTCCTCAAACCTACTG
AGCCACAATCAGAACTCCTAACAGCTATCTTTTGAGCAACAGTTTGTGCC
CTGGTTCTTTATCTTTGTCGCCCTCATTCCCATCACAGTTTTATCCTTAC
AGCATAGTGAAAGAGCAGGGCTTTGGAGCTGTACATGTCTTATTTTGGTT
TTTGGTTTGGGTTTTCTTTTTGCTACAACCATGGCATGCGGAAGTTTCTA
GGTCAGGTACTAAACTCAAACAATAGCAGCAACTCCAGCATGGCAGTCAC
CTGCCACGGGGGAATCCCTATGTAAGACTAATTTTGAATAATATTTTCTT
AGAACTACAGATTCCTCATTTGCATAAGTGTAGATTATCATGCCTATCCT
TCAAGCAGGATTTCTCAGCCTCAGTGCTATTGACATTTGGGGCCAGTTAA
TTCTTGCTGTGAGGACCTGTCCTATACATTGTAGGATGTTTAGCCACATC
TCTGGCCTCTACCCCCTGGATGCCAGGGGCACCACTAGTTGTGAAAATAA
AAACACTTCTAAACATCGGCAAATATTCTCTTGGGGGCAATACTGCGTCT
GGTTGAGATCCACTGCACTCAAGATATTTGAAAATTTAAAAAGAACCTGT
AAGCTAGCAAAGTGCCTTGTATACTGGAGGTATCTAATGAATGCTAGTAA
CCCTTTTTACTTGCTGTGATCATCCTCATCACCATTTGTTCCCTCTCAAC
ATCCTCAGAACATGACATGCTCTTTGATGCCTCTTGGTGCTCTGTGCTAA
GGCCTCTCTTCCAAATGCTCTCTCTGGAAGCAAGCTGTAATAGTCAAGGA
AATGGCCTTGTAAAACCTGCTCTCATATTCTGTGGTCTTCAGAAACCTCC
ATGGCTTCACCCCCTTGACCAAGGCTTTTATCACTGGCTTCATTCTTCCC
CATCCTCCTGCCCCTAATTTACTTCCAGGGCTTATCTGATTTTTAGGTGA
AAAAAATTTCTTTATCAGTGACAAGACTCCAACGAAATGACATCAACACT
TTAGAACTTAATTTATATAATGATACCCACAAAATTGGAGCCACCTAAGT
AGACCAGCCCTTACCATAAATTTAAACCTAAGTCAGTCTGCACTTACAGG
AAACTTCTTTTGAGGAACCTAACATACACCAATCACAGTCTTCCAATTCA

GCTTTAGCCAGTTTACCTTACCCTAGAAACTACAGCCTGCTAGCCTTTTA
AGGAAATTCCCCACCTCCTACCCAATCATGTTCTGTTTCCACATTGCTTC
TTCTAAAGAGCCGTAAGGCTTGGTCTTGCCCAGATCCCTTGGGAAGAGTT
CTCCACTGCTTATGAGGAACTGTATACACCCAGTCCACAGCTTGTCTTCT
CTTGAATAAAGGATATCAAATTTGTAACTGAATTGGTTCAGTTTTTTCAT
TGAACAGATCAGGCATATCTCATTTTATTGTGCTTGCTTTATTGCGCTTT
GCAGATAATTGCATTTTTTTTTACAAATTGAAGGTTTGTGGCAATCCCGG
CTGAGTCTATCACTATCACTTTTTCCAATGCATTTGGCTCACTTTGTGTC
TCTGTGTCATATGTTGGCAATTCTCACAATATTTCAAATTTTTCCTTCCT
ATTATATGTGTTACGATGTTTTGTGATCAGTGATCTTTGATGTTACCATG
GAAATTGGCCCTTTTTAGCTATAATGTCTTCTTAAATGAAGATATATATG
TTATTTTATAGACATAATGTTATTGCACACTTCCTAGACTATAGTATAGT
GTAAACATAACTTTTATGTGCACTGGGAAACCAAAATTTGTGTGATTCAT
TTTATTGCAATATTCACTTTATTTCAGTGGTCTGGAACCAAACCCGAGAT
CTCCCTGATGTATGCCTATAATGTTATGAAGTGTGATATGAAAACAGCTA
CTGAAGATTGCAAGGTNNNNNNNTTGGCAAAGGTAAAGATTCCTATGGAA
CCACTTCAGTTTTCTGCCTCTCTATCTACTCTCCATGATCTCCAATAAAT
GTCTCTTACATTGAGTTCCACCTATTTCCCATTTTGAGCACAAGTTCATT
CGTTCTAATCCTTGTAGTTTGAAAATGGGGGTGCGAAAGGCTCCAGTTTT
TATTGTTGTTGTTGTTGTTGTTTGCTTTTTAGGGCTGCACCTGCAGCATA
TGAAATTTCCCAGGCTAGGAGCCAATCAGAGCTACATCTGCTGGCCTACA
TCACGGCCACAGCAACACCAGACCTGAGCCACGTCTGTGACCTATACCAT
AGCTCATGGAAATGCTGGATCCTTTAACCCACTGAGCAAGGCCATGGATC
AAACCCATGTCCTCATGAAGGTTAGTCGGGCTTGTTGCCGCTGAGCCACA
ATGGAGCAACAATGGGAACCCCCAGATTTTTGACAATGTGGAAAACTGAA
GTATGACAGACTCCAGATATTTCGTAGTGAGACGTAGGGGGCCACAACAT
CTGAGTGGGTGCCCAATCTCATCTCCAATGCCAACACCTTCAGAAGGTAT
TGACAGATCTCTGGAACAGAACCCGGCTAGCCAGAAACAGTAAACCTGGT
TGAACTAGAGGAAAATACATAAACAAGGGAAAATAATAGGTGTCCCTCAA
TCAAAAGAAAACATGATGTTTTCCATTAGGAAACTCACAATGCTTGAGTT
AAGTCCATGGCATAAACTGCTGGACAGGTTGGAGAAATTAATTAAGACAA
TAGATTTGGACTATTTTGGCCAAAAGCACATCAAAATATGTGTAGGCACA
AATCCAACTATTGAAAGCCGTCAGTACATCTGCCACTGTAAAGAAATACT
CTCATGAAAGGAGTAAAGGTCTGCTGTGAATGGGAATCTCTGAAACCAGA
GCAGCAAAAGTGGTGAGCACAGATCAAGCACTTAAAAATCTCATAAAAAG
GGAAAAGGTCTGCTGTGAATGGGAAGCTCTCAAACAACAGGAGCAAAAAA
GGGGAGAACACAACAAAAACTTAAAAAGCTGTTAGAGCCCTCATCGCCTC
ACTCTCAGACTCCTGGGCTAGGATAAATTGGTCAAGGAATGGGTGAGATC
AACAGCAGGTCACATGATACCCTCAAGAAAATTTCCATGTAACCAGATAC
ACTGTTACATACCACATAACCCTCAATCCAGTGGTATATTCCTATTAGGC
TCTGAGAAATCCAAAGCTTAGCTACAGAAATGGACCCCTCAAATTTCAAA
GAACTAAGCATGCCACCTTCTGGCACACATGCCTATTTTATATCTGAGGA
ATGCAGTCCAAAAAACGATAGATATCTACAAAAAACAGCATCATCTTACT
AAAACAACCTGGAATTACAACGGCCATTATAGGGAATCTTCCAGTTACAC
AAGATCATTCCTTTAAGAGTGCACTTGAGGAGTTCCCGCCATGGCTCAGC
AGAAACAAATCTGACAAGTATCTATATACTCAGACGGAAAGAAAATATTC
CAAAGACAAACTGCCACACATCTAGAACATAGACTTCTTTTGATTTGTAT
CTTAAAGATCTTCTCCCTGGTATGAAGAAGCAAATTGGGAGTTCCCGTGG
TGGCGCAGTGATAAAGAACCTGACTAGTATCCATGAGGATTTGGGTTTGA
TCCCTGGCCTCGCTCAGTGGGTTAAGGATCCGGTGTTTCTCTAAGCTAAG
GTGTAGGTTGCAGACGCACCTTGGATCTGGTGTTGCTGTAGCTGCAGCTA
CAGCTGCGATTTGACCCCTAGTCTGGGAACTTCCATGTGCCACAGGTGCA
GCCCTAAAAAAGAAAAAAACCAAACAAACAAACAAAAAAAAAGCAAATTGA
AGATAATGTAGTTGTGTGGATGGGTCAACCGTTTGAGGTCATACAGGCTA
CAATCCAATTCCTTGAGGAATTAAAAACTGTCCTTGTTTTATAGGACCAG
AAATGCAGTTCTAGAAACAATTCAAATCTCACGTATCCTTTTTACCAGTT
CCAAAACTTCCTCATTCATCTCAAAAAGATCATAGGCATTGTAAAATTCT
GGATTTAGAAAATAAAATTTTTTCTTGGAGGAACTTGCATTGTTTCCCTG
TGACATTTCTTTGATATTCAAACTTCACATATATATATATATTTGTCTTT
TTAGGGCCCAACCAGGGTATATGGAGTCAAATTGGAGCTATAGCTGCTGG
GCTACACCACAGCCACAGCAATGCTGGATCTGGCTGTGTCTGTGACCTGC

GCACAGCTCAAGGCAACGCCGGATCCTTAACCCACTGAGCGAGGCCAGGA
ATCAAAACTGCGTCATCGCTGATACTAGTCAGATTAGTTTCTGCTGAGCC
ATGACAGTAACTCCAGAGAGATAATTCTTATTAAGAAGAAAAAAAAAAAA
AAAAGAGGAAAGGTTATTTGGAAGACATAAATGAAAAATTTTCAGTCTTC
ATAAAATTAGTAAAAAATTAAGCCATCAGAGCAGGTAGGCTTAACTTATT
TCACCTGCCAGGAAAACCATTTTGATCCAACAGTTCTTTTATAAACTACT
GAGTTTTGCATTATCACAGCTATTTTATTTTATTTTTTGGCCACAGTGTG
CAACAGTTTGATGTGGGATATCAGTTCCCAAACTAGGAATTGAACCCAGG
CCATAGGGTGAAAACGCTGAGTCCTAACCACTAGATCATCAGGGAAAGCC
CTCGTGGCTAAAATTTTAAAATGAAAGCTTTAAGACTTCTGTTTGCTTCT
GTCTCTATGTTTATGTATGTCTACATATGGATGTTACAAATGTGTGGATT
TTTTCTACCTTTGGATGGCATTGCCAAAACTGATTTGTAAAGAGCTCTGC
TTAATTGGCTTAAAGAAATTTAAGAATTTATATAAATCACCTATACTCTC
AGTAATACAGAAATTAACTCAAATGTTTGCCAAGGACACATGGTCTAGGA
TAACCTCCTGTAAAACAAAGCTAGTTTAAATTTGTTGGTTTAATCCAAAC
AGGAGTGTCTTTAGAGTTATCAGCATTAAATATAATACTTTTAATCTACC
TAGGTTTGCTAAAAACCAAATAAGCTCATGTTATCTCTGTTACAGAATTT
GTGAGCAAGAAAGATAACATGATGATTAGCTGTTTAATGTCTTTTAAATT
CTCAGAAGGAATCTAAACATAATTGTCAAGAACAAGTGAAGTTTAGAAGT
GAAATTTTCAACAATTATTATGTTTTGTAGTACATCTACCTAAAAAAAAA
TAGTTTCCAGGAGTTACCGTCGTGGTNNNNNNNNNNNNNNNNNNNNNNNN
NNNNNNNNNNNNNNNNNNNNNNNNNNNNNNNNNNNNNNNNNNNNNNNNNN
NNNNNNNNNNNNNNNNNNNNNNNNNNNNNNNNNNNNNNNNNNNNNNNNNN
NNNNNNNNNNNNNNNNNNNNNNNNNNNNNNNNNNNNNNNNNNNNNNNNNN
NNNNNNNNNNNNNNNNNNNNNNNNNNNNNNNNNNNNNNNNNNNNNNNNNN
NNNNNNNNNNNNNNNNNNNNNNNNNNNAAATAGTTTCCCAAATTTTTTTGC
TTACTTGAATCCTAAAGTTACACCAAAATAAGTAAAATGGTGACTATCCA
CTGAATATCTAGATCATTTCCCAATAAGATAAAATACTGAAACATTAATT
ACTAAGCATAGGTTTATCCACTTTTGGCTTCCTTTTACAGAAGAACTAAA
TATATTTGGATTTCTTAGTAAACATTGTTTGCGCCACACTGAAAAAAAAA
ATGTATTGAGGAAGAATACATTTCTAGAAATATTTGCAAATTTGCCAATC
CACAGAATTCTAGTGCAACAGACAGTCCACAATTGTTTTCACTAGGAATT
AAGAGATTCTTAGGTAAGAACTATAATTAATATATGTAATTAAAATTACC
AGAAATAATAAGGGAAACATCACTATGCAAGATGTGGAAATGTGGGTTTT
TTGTTGTTGTTTGTTTGTCTTTTTTTTTTTTTTTTTTTGTCTTATTAAGGC
CGCACCTGAGGCATATGGAGGTTCCCAGGCTAGGGGTCCAACCGGAGCTG
CAGCTGCCAGCCTACGCCACAGCCACAGCAACACCAGATCCGAGCCATGT
CTGCCACCCACACCACAGCTCACGGCAATGCCAGATCCCCAGCCCACTGA
GCAAGGCCAGGGATTGAACCCACAACCCCATGGTTCGTAGTCGGATTTGT
CTCCGCCGTGCCACAACGGGAACTCTGAGAGATGTGTTTTTGTTAAGGAA
CAAAAGAGAGTAATTTTTTCCTAAAGTAAGAAGATTATCCAGAATGAGAA
AGAAAGGGGGAAAAAAAAAAGACAAAACTAGAATTGATATAAAAAGTTGT
GGAATATTTGTGGAAAAAATATTCCTTGGAAAGAAATTTGGAATATGGTCA
AGCTGGAAGGTATGGCTAAAATTGAAATGCATTTATTTAAGTTTTGGAAT
TTTTTAAATGAATTTTAATAGCAAAAATACTCTGGGGCAAAAGTTGAATT
GGTTTTCTGTCTTTTAAAAGAGCAAAGTTTTATTAGACTATTGGTCTGCT
CTTGACAAATGTTCGTGAAAGTTTAATATTATTATTTTTTAATTTTTCAG
GTAATCTACCTGGAAAGCAAATATTTTATGCCTTACCAGAATAATTTCCT
ATGCTTCATGTTAACAAATGTCTTTGCTAAGTCATTGATTACTTAAGAAA
ATTCAGTCTTCTCAATATTGAAAGAACTAAGTCTTGGTCACAAATACGTA
ACCTTCTGCATTTGTCTTTAAAATATTTTTTTGTCACTTAGTTAAATAGA
TAATTAGGTACTGTTTCATAATCTCTGTGATCCTAGTCAAGTGTTCAAAC
CTTTTCTCATTTTCACAATTCCCCAAATCAAATTATAAATGCAGCATTTT
TAACCCTCAATGTAAAAACTGGGATTTTTATTAGAGCACCCCCTAGAAATT
TCAAAGGCTTTTTCTCTGATTGTAAAGGAGAGATGTTAAATTAATTAGGT
TTATTTGGCATATTAAATTACGTGGGAAACATTGGCAAATAGGAAGTGAT
GATAAACCTTAAGTTATATATGTGTGGATGTATGTTACTGATATAGGTAT
TCCAGAAATTGTTTAAAGATCCTAGAATTTTGTCAACACTCTCATTGTTG
AATTATTATCTTAGTTTATTGTATGCCACAGAATTAACTACATTTCCTTA
TGAAATGAACTCTCATCAGATTTTTATCTTGAGCATCTTTAATTTTTTTC
ATTAGCAGACATTTATTATTTTACTCTAATGCTTTTGCAAAAGTGAGACT

CATGGAAGGGACTCTACCAAGAATTCTTGACCACTGACCCCTGTAGGCCA
ATTACAAGTTATTGAAACTTGCATGTATATTTCACAGCTTAAAAAGACTC
CACCTGACATCTGGTCCTGAACAAACACTCGAAATCAAGCTTACTTGAAT
GAGAAGCTACTGACATCATGAAGTAGAATGCTTCCACCAGAGAACAAGAA
TCCCAGTCCCACTCTCTTCATGAAGCAGCTTCTCCCTTTTGTACCTGCTT
CATCATTCTTCTTTCTCTTTCTGTCATTTATACAGATAATGCTATTATCT
ATATTTCTGGGGCCCTTGCAAAGGGGGAGACCTATTTGATTAGTGGGTTT
GCCACCAAAGACCCCCACAAGGTCTAGGATGCTGGTGATCCTGCAGTTCT
GCCTGTTACAAACTTCTCCCTGATTTCCAATGCCTCTTTCTCTGGACTAA
ATTAAACCCCCTCTCTATCCTGTCAGATTCCTAACCCTCACCCTACAAAT
CAATTTCTCTGCCTCCAGTTAACAACAAATAGAGAGGGAAATCAGAACAA
TATTCTCTGTGCAGTCTACAGACCCTTAAATCTTAAATCATAAGTCATCT
GATTTCCAGAGCTTTAAGCTTTCCAATTTTGAGCCTTGACTTATCACTCT
GCACTCCCATTCCAATATTCTCCACCACAGATATACCATCTGTGCTCTTC
CTGGATTCATCTTTATCTGTGGACAATTAGTGTCCCCTCCAGTATAGGAA
GCTCAAATTTATTTATTTGTTTTTTAATCTTTTGTCTTTTTAGGGCCACA
CTGCAGCATAGGGAGGTTCCCAGGCTAGGGNNNNNNTCGGAGCTACAGCT
GCTGGCCTACACCAGAGCCACAGCAATGCCAGATCCAAGGCGCATCTGTG
ACCTACACCACAGCTCATGGCAGCACCAGATCCTTAACCCACTGAGCGGG
GCCAGGAATCGAACCCGCAACCTCATGGTTCCTAGTCAGATTCATTTCTG
CTGTGCCATGATGGGAGCTTGGGGTTAATAGATGCAGACTATTGCCTGCT
GTGTAGTACTAGGAACTGTGTCTAGTCACTTATGATGGAGCATGATAATG
TGAGAAAAAAGAATGTATACATGTATGTGTAACTGGGCCACCATGTTGTA
CAGTAGAAAAAAAAATAATGTATTGGGGAAATTAAAAAAAAAAGCCAACGTA
CTGGACTAGATCAACCAGGTATGTACAAACGCCTTAAAACAGTGCCTGGT
ACATTAAAAGTTATCAATTAAAAAAAAAAAAAAAAAAAAAAAAAAAGGGAGT
TCCCATCGTGGCTCAGCAGAAATGAATCCAACTAGGAACCATGAGGTTGT
AGGTCCGATCCTTGGCCTCGCTCAGTGGGTTAAGGATCAGTGCTGCCATG
AGCTGTGGTGTAGGTCAAAGATGCGGCTTGGATCCTGAGTTGCTATGGTG
TAAGACGGCAGCTGCAGCTCCGATGCAGCCCCTAACCTGGGAACCTCATT
ACGCCGTGGGTGCGGTCCTAAAAAAAAAAAAAAACAAAAAGACAAAAAAA
AAAAAAAGAAAAAAGAAAGAAAACAGCCAACTAACTGACTGAGGTTTCAA
TATCCTAACCTATGGAGAGAGTCAGCCCAATTACAACATCAACAGTAAGT
GATAGCTCAGAGTGGCACTAACACCTTATATTTTGATCCATGCTCTCAGA
GCAACTGAATGTTTGATCACAATGAGAGGAATTGATATAGTTAATTAGTA
TATTGGAATTCTCCAGATGGTAGCCATTCCAGCGGCCCAGCCAAGTCACA
AATCTAAACCTAAGTCAGAGTGCAGTTACAGATAACACCTGTCAAAGGAA
ACCTAAGCTGTATCCATCAGAGTTTCCAAATTCAGCTCTAAGTCGCTTAC
CTTCTAGAAAATAGGACTTTCTAGACTTATAGGAAAATACTGATCTGCCA
GCCAAGCCTGCTTTGTTTTCACATTGCCTCTTCTAATGCCTTATAAAACT
CACTCTTGCCCAACATCCCTCGGGAAATGTGCTTCACTTTAGAAAAAGAC
ACAATACTCCTGTTGCTGAAACTCAGCTTCTGTCTGCCAGTGCCATATTG
AAATACAGAGACAGAGTTTGGGGCAAAGGAGAATAAAATAGCTTTATTGC
TTTGCCAGACAAAGGAGGGTCACAGCAGTCTAATGCCCTAAGGACTGTGC
CCCCCATTAGAAAGAACTGTGAGGAGTTTTACAGTAAAAAGGAGAAAAGC
GGGTTTTCAGATAGGAATCAGGATTGGAACAAGCAAGCATTCTTCTTTCT
TTGGGGGAATATTAGTCATCAAAGCTGGAGTCAGGAGATCTTTGCATGAT
CCTGATGGTGGTCTTCTGGGTTACTGCCTAGAATAACAGTACTTGCGAAA
AGGGCATATTGATCAGGGATTAGGACAAACTAGGAAAGTTCCTGAAAATC
ATCATGTGCTAATAATCTTTAACTCACAGGTGATTATGCTCAGGGTGCCT
AATCTTTAGCTTTCAGGTGATTGTGCAATTAAGCCAGGGATGAGGACAAG
GAAGGACTACAGGCTGTTTGGTTTTAAAGGGATTCCTTTCTGAAAGAAGC
ATAAGGAATACAACTTTTAGGCATATAAGATGCCTATATTATTATGTATA
TTCCTTAAGAGGGAACCAGGATCTTGCCCCAAGGCTGTACTATTTTTCCT
TGACTGTTCCTCCCTTGTCTTTGCATCCCCTTCCTTCCCTGATCAGCAAC
TGTCTGAACCTGTCCTTGGGAACTCAGGGAAGGCCATGATGGCTGAGTGA
GGCCCATTTCCTAAGAACAAGAAATAAGAGACACAGAAATGATTTTGTGC
CCAGGAGCCCCACAGGGCCATGCTCGGTTTCACTCCTTCAGTCCATGGAC
TGCTTTTCCTTGAATAAAGAACATCAAACTTGGAGTTCCTGCTGTGGCAC
AGTAGATTAAGGATCAGTGTCACCACAACTGTGGCATAGGTTTCAACTGT
GGCTCAGGTTTGATCCCTGGCCCAGGAACTTTTGGTGGGGACAAAAAGAA

AAAACATCATATTTGTTCTTTAATTGTTTTAGTTTTGTCAGTGACAAGAC
AAAAGCAAAGACCAATATATGGTTAACACACTAAGAGACCAAAATTAATA
GTTACTCTGTAAGGATATGAATGCTTACGCTGGAAGAGGCACTTTACACA
TATTACCTATAACGCTTACAATTTCAGAATGTAAATTTAGTCATCCTCAC
TTGCACACAAATAAACTATTGAGATAAACTAACACATATTAACATTTTTA
AGAGTTTATTTGAGCAAAAATCAATTCCAATTGGGCAGTGCAAAACTAGA
AATGATTAGGAGTGCTCCATAAATAAGAACTTGGAGAAATTCTTTTATAG
AGAAAAGGTGAAAGCAAAGCAAGGAAATTATTTAATTGGCTATAGCTTAA
GCTGTTGCATTCTTTGGAAAATACAATTGGCTGTTTTTAATTTATTGTAA
ATGGGACCAGAGTAGGCCACCCCAAAAATATGCCTCTTTGGCAAAAGTAT
TATCTTGATCTGGTTATTTTTAACAAACAGGGCATACTGGAAAAGCACTA
AAAGCTGAGAAGTTACCCTTTGTAAGGGAAATTTACATTTATAAGGGAAA
TCTAAATTTATGAGTGTCTCTCTATATATACCAGGAAGAGAAGGCTGATT
CTAAATCCCAAAAAAATTCTTATCAATGGAGAAGGCTCAGACTCAAATCTT
CCTAACAACTTTACCCTTTACTGTGCTTTTTTGATAACCTCCTATAACTG
GATCCCCAAACTCCAGAGTCTTTCTTTTGCCTTCAGCAGATGATAGCATT
TAAGGGTGATGGCTTGGACTATTTTGGAGAGTCACACAGTTTTCCTAGGT
ATCTCCCATGTACCCAGGAGGTATGCGTGTTATTAAAATTCTGGTTTTTT
TCCTGTTAATCTCTCTTTTATTAAGGTGAGAGGCGATGTCTCAGGCAAGA
CCTTGGAAGGGTAGAGGGAAAACTTTTTCCTCCCCTACAGAACCAAAACA
GAAAGAGGAGTCTTTCTTTCCCATTATTGTTGGGGGGAAACAAACAAACC
TAAAGAAAGGACAAAGTACAAGGAAATGTTTCTAAAGGAAAATGATTACA
AATATAAGGGGCCCACTGTTTATTCAAAACTGAAATAAGCTAATGAGAAA
GGAGAGTTCTTTAAAAGGGACAGATTGACTTAAAGTATATATGTCTAGAA
ATAGGAAATGTATGAATAACTCAAACATCAGAAAAGCCTGGTGATTAAGA
GAGTGGACTGTAGATAGGTAGACATAGATCAAAAGCCTTTTTCCTTAACA
GCTGTGTAATCTTAGATATTTTAATCTTTCTTTCTAAGCTCCATGGCCTC
ATCTGTAAAATGGAAATTACTTTGCTATTTACCTTATAATGTTGTGAAGA
TTAAATGAGAAAATCCATAAGCCATAAAATTCAACTGAGTAAATTTTAAA
TATCTTATTGGGTTTATTCAATAATTCATGAATCAGGCGGCATGCAATCT
AGCAAACAGAAAGGAGCTCTGAGGAGCTGCACAAAATGAAAACCTTTTAT
AGGCACACAAGAGCAGAAAAAAAGGAAGTTGCTCTAGGCCAAAAAGCAGAT
TGGTTATGGCAAGTTTACTTTCCTTTAGAGGATGGTATCACTGTTGGGCT
GCATCCTGCAGGCCTATAAGCCTGCACTTGCCCAGGTTTAAAACCAAAGA
AAGAGCCTGGAATCAGCAACAAAGACATCAATAGTTTAATGGATGGAGGA
GTTTACACTTCTGAACCAAGGTCCTAGAATGATACCTCACCACGTGCAGC
AGATGACAGGCAGGACAGGGCAGCAGGCTTCACTCCCTGAGGGAATGGGA
GATTGCCAGTTACAGGAGGCTGGCTTATTAGTTACCAGGGAAGGCAGCAG
AGGGGCACATCCCTCACTGCCCCTTTGACATGAACGATCACCCACTGGTG
CCTGGGGCATGTAGGAAGTTCAGCCATGTGAATAAAGTACAGGTGAAGTA
GACACTGGTCAGCCAGGGGATGTACAAAGAGCAAGAGAACGGCCATCTTT
GGTGACCTGACCATACAACCACACTCCCTGATTTCAAACTATACTAAAAG
TTATAGCCATCAAAATAGTATGGTTCAGACCCAAACCCAACACATAGATC
CCTGAAACAGAATAGAGAGCCCATAAATGAACCCATACTTATATGTTTAA
TTAACCTATGACAAAGGAGGCAGGGATATACTATAGGGAAAGACAACCTC
TTCAATAAATGGTATTGGAAAAACTGGATATCTACATGCAAAAGAATCAA
ACTGGACTATGTTTTCACACCATATATAAGATGGGTTAAAGAATTAACTA
TAAGACATAAACCATAAACATTCTAGAATAAAACACAGGGGTATGCTCTT
TGACATCAGTCTTAGCAATATTTTTTTGGATATGCCTTCTCAGGCAATGG
AAACAAAGGCAAAAATAAACAAATGTCACTACATTGAACTAAAAAGATTT
TGCACAACAAAAAAAGTGATCACAAAAATGAAAAGACCGCCTGCTGAATG
GAGGAAGATACTTGCAAACGATATCTGAAAGGGGTTAATATTTCAAAGTA
TACAAAGAACTCACACAACCTCACAACGAAAACAAAAAAATTAAAAAACA
ACGCAATTAAAAAATGGCAGAGATTGGAGTTCCCACTGTGGCACAGCAGA
AGTGAATCTGACTAGGAACCATGAGGTTGCGGGTTTGATCCCTGGCCTCG
CTCAGTGGGTTAGGGATCTGGCGTTGCTGTGAGCTGTGGTGCAGCTCCCA
GACTCGGCTTGGATCTGGCATTGTTGTGGCTGTGGCTGTGGCGTTGGTCA
GCGGCTACGGCTCCGATTAGACCCCTAACCTGGGAACCTCCATATGCCGA
GGGTGTGACCCTGAAAAGACAAAAAAGACACACGCACACACACAAAATTG
GCATAGATTCATGTGCCCCCTCCAAAAAACAAAAAAACAACAACCAAAAA
AAGGCAGAGGACTTGAACAGACACTTTTCCCAAGAAGACTTACAAATGGC

CAAAAGGCACATGAAAAGATGCTTAGGATGAGATATCACTTCACACCTAT
CAGAATAGTTATTATCAAAAGAATCAATAACAAGTCTTGCTAAGGATGTG
GAGAAAAGGAAACCTTTGCACACTGTTGGTGGGAATGCAAATTGGGCAGC
CACTATGGAAAACAGTATTTGAGGAAATGCCTCAAAAAATATATGATCTA
GCAATCCCACATCTCGGTAGCTATCTGGAGAAAAACATAATTCAAAAAGG
TATAGGCAGGAGTTCCCATCATGGCTCAGAGGTAACAAACCCATCTAGTA
TCCATGACGACATGGGTTCGATCCCTGGCCTCGCTCTCAGTGGGTTAAAG
ATCTGGCATCTGTGGTGTAGGTTGCAGACTCAGCTCAGATCCCAAGTTGC
TGTGGCATAGGTTGGAGGCTGCTGCTCTGATTTGACCACTAGCCTGGGGA
CTTTCATATGCCATAGGTGTGGACCTAAAAAAAGCCAAAAAAAAAAAATAG
CATGCCCCCCCCNNCCATGTTCATTGCAGCATTCATTACAATAGCCAAGT
CATAGAGGCAACCTAAACATCCATTGACAGAGGAATGGATAAAGAAAATG
TGGTTTATATACCCAAAGTGATATTATCAGCCATAGCAAATAGTGAAATA
ACGCCATTTGCAGCAATATGGATGCACCTAAGATTATCCTACTAAGTGAA
GTTTGTCAAACAGTAAAAGACAAAGACCATATGATATCACTTATATGTGG
AATCTAAAAAAAGGATACAAATAAAATATTTCTAGTTTGAAGTTCAGAAA
TAGATTCACAGACATTGAAAATAAACTTATGGTTACCAAAGGGGACATGC
AAGAAGGAGGGATTAACTGGGGATTTGGGATTGGCATATGCACACTGTGG
TATATAGAATGACTGGCCAAATGGGACCTGCTATATAGCACATGGAACTC
TACCCGGTATTCTGTGATAATCCATATAGGGAAAGAATCTGAAAAAGAAT
GGATGTGTATATATGTGTAACTGAATCAGTGTTTTGTACAGTAGAAATAA
TTACAACATCATAAATCAACTATACCTCAATAAAACTTTTAAAAAATGAA
AAAAATATATTAAAAAAAATTTTAAATTCGAGCCACCATACAATCCAGCA
ATTCCACTCTTGTGTATTTGATGAAAAGGAAAACACTAATCAGAAAAGAT
ATATGCACCCCAATATTCATTGCAGCATTACTTACAACAGCCAAGATATG
GAAGCAACATTAGTGCCCATCAATCAATGAATGAATAAAGAAGGTGTGGT
GTATATACACAATGGACTATTACTTAGCCATTAAAAAAAAAGAAGAAGAA
ATCTTGCATGTGTTACACCATGGATGGACCTAAAGAGTATTATGCTAAGT
GAACTAGTCAGAGAGACAAACACTGCATAATTTCACTTATATGTGGAGTC
TAAGAAAATAAATGAACAAGCATAACTAAGTAGAGGCAGTCATAAATACA
GAAAACAAACAGGTAGTTGCCAGAGGGGAGGGGGATTGCCAGAAAGAGAA
ACAGGTGAGGGAGATTAAGAGGTACAAATTTTTGGTAGCAAAATAAATGA
GTCTTGAATATGAAATGTACTGGGTGGGTAACATAGTCAATTAAAAAAAA
AAAGACTGGGATAGTCTAGCCCACTGCTTCCAGTGGGGGATAGTGTTCAA
GGACCTGCTTTGGGAAAGACAGATAAATGCTATAAACTCAAATGACTTCA
GCTCTAGACATTGTCAGATTCCAGCCACTTGATTCAGAAGATGCTCTTTT
CTCTTTTCTCTGCTTGATTGGGTTTTGTTGCTCTTCCTCATAAAACACTG
ACTGCCCTTTCAGGGATCCCTGTAATTGCTTTATCAGTCCTTTCTAGGGT
TTCCTTTTTACAGCCACTTCCTCAAGAGCTGAGCCACCCTAGGCAACACT
ACAATCAACTGCTGACCTCAAAGGAGGAGGAAGTCTGGTAGGAAAGGCAA
CCACAGAAGGGGAAGAAGAGAGAAGGAATGATGAAGTTATAAAAGACGGT
TTGAAGGAGCTCAGCTAGATCTACACAACGGCATCGCCAGTCTACCTAGG
AAATCTTCCTGTGGGAATTGATGTCTTTGTGTTAAAGCATTTAACACACA
TTGAGGTCTTTAAAGCACGTCACCTTTAACAACCTTTTAGAAATGCAGAT
ACCCTAAACTCTGTGGATTACTAACTTCAGAGTATGGATTTGTTCGGTCT
TTTTCAGAATAGCAGAGTGCTGAAGAATTTTCATGAGAGGAGAGAGAAAT
CTGAGAGAACGAAGGGCTATTTCTCAAGAAGCAAGGACCCGTGAAAAATG
AGGGGCTTAAACAACAGAGGGCTATTTATCTCCGAGGACCCATATTGGGA
AGGGAAAGATAAGACAGATTTCAGACCCAGCTTTGCCACTATGTGGTCAT
GGGAAAGGCACTTTTCTTCTCTGGGCTCAGTAAAGTAAGGGGGATGGACA
CGATGCTCTTTATCAGATATGATTTTTGCAAGTATCTTCTCCCAAGTATG
GCTTGTCTTCTTACTCTCTTGACAGTGTCTTTTGCAGAGCCAAAGTTTTA
AATTTTATTAAAGTCAAGTATCAATTATTTCTTTCACACCTTTTTTTTTT
TTTTTTTTTTTTTTTGTCTTTTTGCTATTTCTTGGGCTGCTCCGGTGGC
ATATGGAGGTTCCCAAGCTAGGGGTCGAATCAGAGCTGTAGCCACCGGCC
TATGCCAGAGCCACAGCAACATGGAATCTGAGCCGCATCTGCGACCTACA
CCACAGCTCACGGCAACACTGGATCGTTAACCCACTGAGCAAGGGCAGGG
ACCGAACCCACAACCTCATGGTTCCTAGTCGGATTCGTTAACCACTGCGC
CATGACAGGAACTCCTCTTTCACACTTTTGATGTTGTATTTAAAAAATCA
TTGTCTGGACACAATAGAGATTGTCATACTAAAGGATAGGTTCCCGGAAG
GATAGTGACAGGACCCCACCCCCACCCCACCCCCGGCCCCTGAATGTGGC

TGTTCTCCTTGGAGACCTGCTGCAGACATGGGTATGGCCAGCACGAGATT
TATTCCCTTTCCCAGAATGCAGATGCTCAAGTGGGGCAGCAAGAAACAAT
GGCCTACAGGAAAACAACTGCCTGCATAGATTAACAACCCACAAGGATTA
ACTGCCTTCTGTCATGGGACTTCATCCCCTACCCTTCCCCTCTTCTTCCT
TCACTGTTCCTGTCACAAGTTCCCCCCATAAACTCCGACCACAGATTGCC
CCACAGATCCCTTATAAGCCTAGCATCCCCTCACAGTTGGCGCTGGTACC
ATCTTGAGCTCAGCCCATCCACTTCAGATGCCTTAGTAGATCTCTCTCAA
TTTACTGACTTGACTTTGCTCATTCCTCCTTTGGCAAACACTAAGTGAAG
TAAATCAGAGATAGAAGGACAAATACCATATGATATCTTGTATATGCAGA
ATCTAAAATACATCACAAATGAACCTATCTACCAAACAGAAACAGACACA
TGGACAAGGAGAACAGACTTGTAGTTGCCAAGGGGGAGGGAATTGGGGAG
ATAGATTGGGAGGTTGAAGTTAGCAGATGTAAGCTATTAACATGGAGGGG
ATAAACAATATGGTCCCACTATGTAGCACAGAGAACCATATTCAATGTCC
TATGATAAACCATAATAGAAAAAAATATTTTATTTTATTTTGGTTTTTTT
TTTTTTTTTTTTTnnnnnnnGTCTGTTTGCCATTTCTTGGGCCGCTCCCA
TGGCATAnGGAGGTTCCCAGGCTAGGAGTCGAATCAGAGCTGTAGCCACC
GGCCTACGCCAGAGCCACAGCAATGCGGGATCAAGCTGCGTCTGCnACCT
ACACCACAGCTCACGGCAACATCAGATCTTTAACCCACTGAGCAAGGCCA
GGGATGGAACCCGCAACCTCATGGTTCCTAGTCGGATTCGTTAACCACTG
CGCCATGACGGGAACTCCATAAAAAAAGAATATTTTAAAAAGAGTGTATG
TGTATATATATATTCAGAAATATATCTGAATCACTTTGCTGTACAGCAGA
AATTAACACAATATTGTAAATCAACTATACTTCAGTAAAAAAAGTAAAAT
TTAAAAATACATGATAAGTCATTTTATACTCAAGGTCATTTAGATTTTTC
ACCTTTATTATCTTCTAGGGTTTTATAATTTTGTGTTTTGCATTTAAGTC
TATGATCCATTTCTTCTTTTTTTTTTTTTnCGTCTTTTGTCTTTTGAGGG
CCACACCCTCAGCACCCAGAGCACATGGAGATTCCCAGATTAGGGGTCAA
ATCGGAGCTGTTGCTGCCGGACTACACCAGAGCCACAGCAATGCCAGATC
TGAACTGTGTCTGCAAACTACACCATAGCTCATGGCAACACCAGATCCTT
AACCCAGTGAGCAAGGCCAGGGATTGAACCCGCAACTTCATTGTTCCTAG
TGGATTCATTTCCACTGCGACATGACAGGAACTCCTATGATCCATTTCAA
GTTAATTTTTTGTGAAGTGTGTATGTTTGGTATCTAGATTCCTTTTTTTT
TCTTTTTGGTCTTGGATGCCTAGTTGTTGAAAAAACTATCTTGGAGTTCC
CGTTGTGGCTCAGCACTAGTGAAACCCGACTGATATCCATGAGGATGTGG
ATTTGATCCCTGGCCTTCCTCAGTGGGTTAAGGATCTAGCATTGCCCTGA
GCTGTGGTGCAGGTTACAGACAAGACTCAGATCTTGCATTGCTCTGGCTG
TGGCATAGGCCAGCAGCTGTAGCTCTGATTCAACCCCTAGCCTTGGAACT
TCCATATGCCACAGGTGTGGCCATAAAAAGCAAAATAAATAAATAAATAA
ATAAAGGCAGTGAGGAAAAAGAAGACTATCTTTTCTCTGTTATACTGCCT
TTGCTTCTTTTTTTTTTTTTTTTTTTTTTTTTTnTnTTTTTnnnnTTTCT
TTGGCCGCTCCCATGGCATATGGAGGTTCCCAGGCTAGGGGTTGAATCGG
AGCTGTAGCTGCCAGCCTACGCCAGAGCCACAGCAACGCGGGATCCAAGC
CGCATCTGCAACCTACACCACAGCTCACGGCAACGCCAGATCGTTAACCC
ACTGAGCAAGGGCAGGGATCGAACCCGCAACCTCATGGTTCCTAGTCGGA
TTCGTTAACCACTGCGCCACGACGGGAACTCCCTTTGCTTGTTTGTAGAC
AATTCTGTTCTAGTGGTTTGTCTATTTTTTTGCCAATAACAGGATATTCT
TTAAGATTTCTTCCTGTTCTAACCAGTTCTTCCTGTTCATTAACCTATAT
GTAAATGAAGAAAAACACAGATTCCTTTCCAATTTTACCCAGCTCAGACT
CCAGACTCTACTTCCTCTTCAATAGTGATTTCCAGGCTGAAAGTGACCAA
TCTTCCACAATCTGTAGGGAACAAGAGAGTATTGGGATCCTGGGTCACAC
CAAGATGGGCTGCTCTCTGGGGCAACCACAGAAGGGTGTGGACAAAGAGA
TAGCTTACAGCGACTGCTATCCTAAGCAGGTGCTATGCTAGAAGGGAAGA
ATAATGCCATCACCAGGAAAGAAAGCAGAAGAGTCAGGAGATGAGAAGAC
CATTAATCAGGTATCTGGAGCAGAAATACTGAAAGATGAGAAGTTGGAGA
CAGATGGAAGGGTGGTAGGTCCAGATTCAGTCTGAAACAAATCTTGTCAA
CATGAAGTTCCCTCCATACATTCCTTTCATGCAATAACTGGCAGATTTCT
AGGAAAGGACAGATCCTAAAAGGATAAGAATATAAATAAAGGATCTCCT
CTGACCATAACAGTGAGGTAAAGTCACAAATATAAAACTAGTATTATTGC
TAGCTGAAGAGTGTTATGGAAAAGTCCCTCCATAGGGGTCACTTTATCAA
TCTAGAAACTGGGAAAAATTACTTTGTACAAGAAGACAGTGGGGGAGTTC
CCATAGTGGCGCAGTGGTTAACGAATCTGACGAGGAACCATGAGGTTGTG
GGTTTGATCCCTGGCCTCGCTCAGTGGTTATGGATCTGGCATTGCCCTGA

GCTATGGTGTAGGTCACAGACGTGGCTCAGATCTGGCATTGCTGTGGCTG
TGGTGTAGGCCGGCGGCTGCAGCTCCGATTGGACCCCTAGCCTGGGAACC
TCCATATGCCGCGGTTGTGGCCCTAAAAAAACAAAAGACAAAAAAAAAAA
AAAGACAGTGGGGGGAGTGCCCATCATGGTGAAGTGGAAACAAATCTGAC
TAGGAGCCACGGGGTTGTGGGTTTGATCCCTGGCCTCGCTCAGTGGTTAT
GGATCTGGCATTGCCCTGAGCTATGGTGTAGGTCACAGACGTGGCTCAGA
TCTGGCATTGCTGTGGCTGTGGTGTAGGCCGGCGGCTGCAGCTCCGATTG
GACCCCTAGCCTGGGAACCTCCATATGCCGCGGTTGTGGCCCTAAAAAGA
CAAAAGACAAAAAAAAAAAAAAGACAGTGGGCATTCCCAGCTAAGCATCT
GTTCTTTCCTATGGCCCCCAGTGTCACTACAGGCCCCAAAAAGAAGACTT
TTCTCTGGCAAAGGTACAGAGTAAGTGGGCAGGCTGGAGTAAACTTGGGC
CCTGTGAGAATTGTAAAATGATACTAGGTCCTCAGGGATAACTGCAGGGC
AATAATGCTGAGGCTGGCAATAGCATCAAATACTAAGTAAGGGAGAACTT
GCCCAAGAGGAAGCAATTAACTAGAGACTGAGTGACTCAATGAACATCCA
GTGCTTTAACCCTCTGTTGTCTCTATACTGATAAAATGGGGGGATGGTGG
GAGGAGAAGATGAACAAATAAGTAGAGACAGGAGTCTCTGCTCTGGGGAA
CTCTCATTATAATGGCAATGACAGGATATATGCAGGAAACATATATAAAA
ACAGAATAAACACATCCTATCTTTCACCTGTGTTACCATAGTAGAAATGA
GCTTTGAGTTTGTGACTAGAATTAGAAATTGAAGACAATTATGAGGATTT
GAAAAGATAATTATTAACTGTAGAGCCAAATCATTTTATCAAAAGGGTTT
TGATTTCAGACACAGCTGCCAATTAGCTGCAAGGTAGGTCTGAATCTCAA
GCAGTATTAACATAAATTTGAGAAAACACTGAGCAACAATAAGAGAGAAG
CTAAGAGATGAAAGTTTACACCATCAATAGTTATGATAGTCATAAAATCA
AAATCATATTTGTATTATAAGCCACAAAGCCTCAGTGAAAACAAATACCA
ACATCACCAGTGGTGAAAACTTAGCACAAAAAAGATTGCAAACTGAACTA
GTGATGAGAAGAACATATAGGAATCCTGACATCATTGCATGGCTCCTGCT
TATGTAAACCCCTGTCATAATTAGGGTCTAATTCTTAATCACTTTTGATG
AGGAGATTTAAAACACACCAGATAAGACAGAAACAGAGGGAAAAAGAAAA
AAAAAATGGAGGTACTAAACAACCAGAAGGCAAATGATAATATGGCAGTA
GAAATTACTTACATATTGATATAAGTAGATTAAATTCACCAATCAAAAAA
TACAGAGGTGAGAAAGTTCCCACTGTGGCTCTGGAGTATTGAGTCAACTA
GTATCCATGAGGATGCAAGCTCAATCCCTGGCCTCACTCAGCGAGTTAAG
GATCCAGTGTTGCCCTGAGCTTTATGTAGGTTGCAGACACAGCTCAGATC
CTGCCTGGCTGTAGCTGTGGTGTAGGCCAGCAGCTGTAGCTCTGGTTTGA
CCCCCAGCCTGGGAACTTCCACATGTCACTGATGTGGCCCTAAAAAGCAA
AGCAAAACAAAACAAAAATAAACAAACAAAACAACCCAAAACCAACCATC
CAAACAAACAAAAACCAGAGTGGGAATTCCCCAGCATTTTAGCAATTAAG
GATTCAGCATTGTCACTGCTATGGCTCAGGTTCTGTCCTGGCCTAGGAAC
TTCTGCATGCTATGGGCAGGGCCAAAAATAAATAAATAATACAGAGTGAC
TGGATGGATTTAAAAAAAAAGAAAAAAAACAAGACCCAACTATGTATTGC
CTACACGAGATTCATTTCAATTCTAAAGACATACATAGGCTCAAAATGAA
GGGTTGGAAAAGGATATCCCACACAAGTGGAAATCAAAAGAAAGAAGGCA
TAGCCATACTTATATCAGACTAAATAGACTTCAAGCCAAAAGTAGTAACA
AGAGACGAAGAAGATGGTTATGAAATGGTCACTACATCAAGGAGCTATTA
CACTTGTAAATAAACACACACACACAATATCAGAGCACCAAACTACATTA
AGCAAATACTAACAGATATAAAGGCAGAAGTAAACAGCAATACAATAATA
ATAGGAACCAGAGTTCCCGTTGTGGCTCAGTGGTTAACGAATCTGACTAG
GAACCATGAGGTTGCGGGTTCGATCCCTGGCTTTGCTCAGTGGGTTAAGG
ATCCAGTGTTGCCATGAGCTGTGGTGTAGGTTGCAGATGTGGTTCGGATT
CCAAATTGCTGTGGCCCTGGCGTAGACCAGCGGCTACAGCTCCGATTCGA
CCCCTAGCCTTGGAACCTCCATATGCCATGAGAGTGGCCCAAGAAATGGC
AAAAAGACAAAAAAAAATAATAATAATAATAATGGGAACCCAGGAACTTT
ACTGAAAAACTGGAAAATAATAAAATTATTTTCAGAATGGGAAAAAAAAA
AACCAATAGTAGGGGAATTTAACACCTCATGGTTAGCAATGGATAAGTCA
TCCAGATAGAAAATCAAAAATGGACAAGTACCTATTGAGTTAACAAACAT
ATATAGAACACTCAACAGTAGCAAAATAGACAGAACACTCTCCAAGACAG
ATTATATGATAGGACCCAAAACAACCCTTAGCAAATTTAAGATTTAACTC
ATACCAACCAACTTTTCTGATTACAGTGGTATAAAACTAGGAATCAAGAG
CTGAAGAAAGCCATTGGAAAATCTGACAACCTCATGGAAACTAAATGACA
CAGTTCTAAACAACCAATGGGTCAAAGAAGAAGTCAAAGGAGAAATAAAA
ATTATATCTAGGAGTTCCTGTCATGGCTCATTGGTTAACAAATCTGACTA

GGAACCATGCTGCAGGTTTGATCCCTGGGTTAAGGATCTGGAGTTTCCAT
GAGCTGCAGTGTAGGTGGCAGACGCAGCTCAGATCCCATGTTTCTGTGGC
TCTCGCGTAGGCCGGCAGCTACGACTTCAATTGGACCCCTAGCCTGGAAA
CCTCCATATGCCATGGGTGTGGCCCTAAAAAGACAAAAAGACAAAAAAAA
TTATTTCTAAACAAATGAAAATGGAAACATTATATCAAAACTTATGGGAT
GCAGAAAAAGCAGTTCTGAGAGGAAAGTTAACCACAATAAATGCATATAT
TAAGAAATTAGACAGCAACATGGATGCAACTAGAGATTCACATACTAAGT
GAAGCAAGTCAGAAAAAGAAAGACAGATATCATATGATATTACTTTTATG
TGGAATCTAAAATATGACACAAATGAACCTATCAACAAAACAGAAACTGA
CACACAAACAGAGAACAGAATTGTGGTTACCAAGAAGAAAAGGGGAGGAG
TGTCTTGGACTGGGAGTTTTAGTTGGTAGATGCAAACTATCATATTTAGA
ATGAATAAGCAATAAGATCCTATTGTATAGCACAGGGAATGAAATCCAGT
CTCTTAGGACAGAACATGATGGAAGATAATATGAGAAAAGAATATATATA
TATGTGCGATTTTTGTCACTTTGTTGTACAGAAGGAATTGATAGGACAGT
GTAAATCAACTATACTTTAATAAAAAATAAAATTTAAAAAATGCCTTTGA
GAAAATAAAGATTTTTAAAAAGCTGAGAAAAAAAAAAGAACTGGAGTTCTG
TCATGGCTCAGTGGTTAACAAACCCGACTAGCATCCATGAGGATGTGGGT
TCAATTCCTGGCCTACCTCAGTGGGTTAAGGATCTGGCATTGCCAAGAGC
TGTGGTTAGGTCAAAGACACGGTTCCGATCCTGCATTGTTGTGGCTCTGG
CGTAGGCTGGCAGCCACAGCTCTGATTAACCCCTAGCCTGGGAACCTGCA
TATGCCACAGGTGTACGGCCCTAAAAGATAAAAGGGACAAAAAAATCAGA
GTTCCCATCATGGCTCAGTGGTTAACGAACCCGACTAGTATCCATGAGGA
TGTGGGCTTGATCCCTGGCCTCAGTGGGTTAGGGATCCCACATTGCTGTG
GCTGTGGTGTAGGCCGGCAGCTATAGCTCTGATTTGACCCCTAGCCTGGG
AACCTCCATATATCACGGATGTGGCCCTAAATAGACAAAAGACAAAAAAC
AAACAAACAAAAAAAACTAATTTTATTCACCTCATGAAACTTGAAAAAGA
ATAAAGTTAGCAAAAGGAAGTAAATAATAAAGATCAAAGTGGAAATAAAT
AAAACAGATACCAGAAAAAAACAATAGAAAGGATTGACAGAACTAAGAAA
TGGCTCTTCAAAAAGCTAAACCAGGAGTTTCCATTGTGGTGCAGTGGAAA
CAAATCTGACTAGTAACTATGAGGTTGCAGGTTCAATCCCTGTCCTCCCT
CAGTGGGTTAAGGATCCGGTGTTGCCAAGAGCTTTAGTGTAGGTCGCAGA
TGACGCTTGGATCCTGAGCTGCTGTGGCTGTGGGGTAGCCTGGAAGCTGT
AGCTCAGATAAGACCCCTAGCTTAGAAACATCCATATGCCGTGGGTGCAG
CCCTAAAAAGCAAAAAAAAAAAAAAAAAAAGCTAAACTGAAATTTAGTTAA
ACTAAGAAAAAAAAAGAGAAGATTCAAATAAATAAAATTAATAAAATTAGA
AATACAAAATGAGACCTTACAACTGACTACAAAAATACATAGTATCATAA
GAGATTATTATGAACAATTAATTATATGCCAGAAAATTAGGTAACCTAGA
AGTGGATAAATTTACAGAAGCACACAACCTACCAAGACTGAATCATCAAC
AGCTCTGATTTGACCCCTAACCTGAAAACCTCCATATGCAGCAAGTGCAG
CTCTAAAAAGCAAAAACAAAACAAAACAAAACTGAATCATCAAGAAATAG
AAAATTTGAACAAACCAATAACAAGCAGGGAGATTTAATCAGTAATAGAA
AAACCCCAGAACAAATGACCTCACTGGAGAATTCCACCAAACATTCAAAG
AACAATTAACACCAATTCTTCTCAAACTCTTCCAAAAAATTGAGGAGGGA
ATACTTCCAAATGCATTCTACTAGGTCAGGATTATGTTATACACTATAAC
CAAGTGGGATTTATCCCTGGGAGGAAAGAATGGTTCAGCACATGTAAATC
AATAATGTAACATATCATATTAATAAAATGAAAGATAAAATCACATAATT
TTCTCAATAGGTGCAAGAAAAATATTTGACAAAATAAAATATTCTTTAAT
GATTAAAAACCCTGGGAAAATTGGGTATAGAAGGAATAATAACATAATAA
AGGCATATATGACAAAACCACAGGTAACATTATACCCAATAGAGAAAAGT
TGAAAATTTTTCCTCTAAGATCAGGAACATGACGAGGATGCCAACTTTCA
CCACTCTTATTCAATATAGTACTGGAAGCCCTAGCTAGACTAGGGCTAGA
CAAAGGAATAAAAGGCATCCAGACCAGGGAGAAGTAAAAATTTCACTTTT
TGCCAATGATACGATTTTATACATAGAAAATCCCAAAGACGCAACAAAAA
TTTTTGGAAATAATCAACAATTTCACTAAAGTTGCAGACTACAAAATCAA
CATACAGATATCAGTTGCATTTTTGTACACTAATGATAAAACAACTGAAA
AAGAAATAAACCTATCCTAGTCACAACAGCTTCAAAAACAAAAAAATACT
TAGGTACAAATTTAACCAAAAGAATTGAACAGCTAAAACTACAAGACTTT
GCTGAAAGAAATTGGAGAAGACATAAACAAATGGAAGGACATTATATGTT
CATGGATCTGAACAATTAATATTATTAAAATGTCCATACACTCAGGTCAT
CTATAGATTAAATCCCCATCAACATACCAATGGCATTCTTCACAGAAATA
GAAAAAAATAATCCTAAAATTTGTATGGGACCACAAAAGACCCCAAATGG

CTAAAAAATACTATTCAGGAAAAAGAACAAAGTCAGAGATAACACACTTC
CTGATTTCAAACTACACTACAAAATTATAGTAATTTAAAAAAAAATAAAA
TAAAAGACCAATGGAATAGAATAGAGAGACCAGAAGTAAATCCTTGCTTA
TATAGTCAACTAATATTTGGTAAGGGAGCAAAGAACACTCAGGGGGGATA
AAATAGACTCTTCAACAAATGGTTTTGGGAAAACTGGACAAACACATGTA
GAGCAATGAAATGGGACCTCTATCTTACAAAACCCACACACACAAAAAAA
TCACTCAAAATGTATCAAAGATTCAAACATGAAACCTGAAGCTACAAAAG
TCCTGTAAGAAAATATAGGGTAGAATTTGTCAATTTTTTTAGATATGACA
ACAAAAGCAAAATTGGCAAATGGGATTACATAAAATTTAGAAGCTTTTGC
ATAGCAAAAGAAACAATGAACCAAATGAAAAGACAACATACAGAATGGGA
GAAAAAACTAGCAAGCCTTGTATTGGATTAGAGGTTAATATACAAAATAT
ATAAAGAACTCATAGAACTCAATAGCAGAAAAAGACCAAAACAAAACAAC
AACGAAACTGAACAACCAAAGAGCCTGATTAAAAATTGGCCAGGGGATTT
CCTGTTATGGCGCAGCAGAAACGAATCTAACTAGTATCCATGAGGACTCA
GGTTTGATTCCTGGCTTCTCTCAGTGGGTTAAGGATCCAGCTTTGCTTTG
AGCTATGGTGTAGCTTGCAGATGTGGCTCAGATCCTGTATAGCTGTGGCT
GTGGCATAGGCCAGCAGCTGTAGCTCTGATTCAACCCCTAACCTGGGAAC
TTCCATATGCCACAGGTATGGCCCTTAAAAAAAAAAAAATGACCAGAATA
GCTAAATAGACATTTTTCCAAAGACATCTAAATAGCCAACAGGTACATGA
AAAGACACCTAACATTACTAATCAGTAGAGAAATACAAATCAAAACCACA
GAGAGATATCACCATATACCTGTTAGAATGACTATCATTAAGAAGACTGT
TGAGGATGTAGAGAAAAAGGAAACTTTATACAGTGTTGGTAGAAATGTAA
ATTGGTTCCACCACTATAGAAAACAATATAGAAGCTCCTTTAAAAATTAA
AAATACTGGAGTTCCTGTCATGGCACAGAGGAAATGAATCTGACTAGCAT
CCATAAGGATGCAGGTTCCATCCCTGACCTTGCTCAGTGGGTTGAGGATC
TGGCATTGCCATGAGCTATGGTATAGGTCGAAGATGTGGCTGGGATCTGG
CATTGCCGTGGCTGTGGTGTAGGCCAGAAGCCACAGCTCCGATTCGACCC
CTAGCCTGAGAACCTCCATATGCTGAGGGTGTGGCCCTTTAAAAAAAAAA
ATTTTTTTAAGTACTACTACCTTGTGAATAAGAATCCTACTTCTCGGTTG
ATATTCGAAGGATATGAAAACAGGTTTAGAAAAGATAAATGCACTCCCAT
GTTCATCATAGCATTGTTTTTATTTTTATTTTTTCCATTATAGTTGATTT
ATAGTGTTCTGTCCATCACAGCATTATTCACAAAAGCCAGGATACGGAAA
CAACCTAAGCACCTAGGAACAGATGAATGGATTAAGAAGATGTGATATAA
ACATGGAGGGTATCCTGCCATTTATGACAATGTGGATAAAACTTGAGCAA
ATTATGCTAAGTGAGATAAGTCACAGTAAGACAAGCACAAGAGTATGATA
TCACTTATATGTTGAATTTTTAAAAGTGAAACTTGTGGAGTTCCTACTGT
GGTTCATTGGCTTAAGACTAAAGCTGCAGCTTAGATTCATCCCCACCCAG
AAATGTCCATATACTGTGGGTGCGGCCATTAAAACAAAATTCATTTATTC
CTTCCAAACTTGCACTTGTTTGTCTATGATCTACTCTGTTACCTAAGTCA
AAATCCTTCACTTATCCTAAATCCCTTATTCTCCTTCATCATTGCTATCC
AATCAATAATTAAATTTTCTCAAAAAAAACCCTTCCTGCTAGTTATTAGC
AGTATAAAACTTTGAGCAAGTCACTTAATTTCATGGTGCTTTGCTTCTCC
CACTTGTAAAATAGGAGCAATAATAGTTCAATTACCTCACCAGTTTGATA
TGAGGGTTAAATAAGTTAATTATTTATTGTTTAGAATGATGCCTGAACAT
AGTAAGTGCTATGTAAGTACTTGCTATTATTATTTTTTCATGTCCTCATT
ATTCTCTGCCCAAAATATTGAAATAGAATCTTAACTAGTCTTCCTTCCTC
TCAGTCTGGCTCCATTACAATCCATCTTCAATAACTGTTTTCAAAAAGAC
TTATCGAAATATTTTTCAACACAACACATCATTTAAGAAGGGAGGGACCA
CAGACTAGGAAAAGATATATGTAATAGTTATATTTGACAAAGGATTCATA
TCAACAATACATAAAGAATTCCCCACCCCCCTCAAAAAAAAGGACTTCCC
ATCGTGGTGCAGCTGAAATGAATCTGACTAGGAACCATGAGGTTGCAGGT
TCGATCCCTGGCCTCGCTCAGTAGGTTAGGGATCCAGCATTGCCGTGAGC
CGTGGTTCAGGTTGCAGACACAGCTCGGATCCCAAGTTGCTGTGGCTGTG
GCATAGGCCAGCAACAGCTCCGATTAGACCCCTAGCTTGGGAACCTCCAT
ATGCCGCAGGTGCAACCCTAAAAAAACAAAAAAGACAACAACAACAAAAA
GAATACATAAAGAATTCCTACAAATCAAAAAGAAGAAGACAGACAAACAA
ATTGAAATGTAAGCAAAAGATTTAAACAAGCACTTCACAAAAGAGGACAT
TTGAATGGCCAATTAAGCACATGAAAAGATACAACATCATAACTTATCAG
GGAAAAGCGAATTAAAAATACAACAAGATATCACTATACATTGATGGGGT
TCAAGATACACTATCCCAAAATGTAGTAGCTTGGTAAATTGAATATTTTA
AGCTGAGGGAGGTTGTTTTTTTAATGGCAGAATCTAGAAGTTCACTCCGA

CCTCCTCCACCTTGCCCTTTCTCCCTGAAAACAGGAGATAAATATCCCAA
GTAAAAACACCCTCCCTATACCAGAAGAAGGGGGGGGGGCATTCTTATCA
ACAGAGATGGGAAGTTTAGGGCCAAGAAGGCTGTATAAACAAACCTTGTT
TACTCTTCACTATTTATTTACTACCCATAGCCCAAACCCCTCTGCCTTGT
CAATTCTTCAAAAATGTGTTGTTTCTTTGTCTGAAAGGCATAAAATCTTC
CTGCTATGTTCACTTCTTCAAGTCTTTTATCTTAGTGGGGCTCCCATTAA
CACATATACAGAATTAATTTTTCTCCTGCTAATATGTCTTATGTCACTTT
AAAAATTAGACTACCCAAAGACAGAGGAGAAGAAGTGGGATATTCCCCCC
GCCCCCCAGCACCCAAACTTTAAAAGATTGAAAATACCAGAATATACCTC
AGTTGAAACGAAAAACTGAAAACCAAAATTGCCTAACACACCAGGTCGCT
GCCCTGTTCAAAATCCTTCGATGACTTCCCATGCCTTCAGGATGATGTTC
AGCCCTTCAAGATGACATGCAAACCCTTCAGGGTAGCACAAAATGTCTGA
GAGCATACCTTTCACTGCCATCCTCACCCCTATCCTATGCTCCAGCAGTA
CCAAACGTCTTGCTGTCCTCCCAATTTGCCATGTATTGCCATATTTTCCC
CCATGCTGTTTTCTGTGAACAAAGCCATTTTGCCAACTCGCCCATTCCTT
AAGACCAAATTCCTTCTCCATAAAGCCTTCCATAAGTTGCACATAGCTAA
ACTTTTGTTTGCAAGAAGAGTCACCTAAATGTTGCTGACTGTCCAGAGGC
TGCCCTGCCTGGACAGAGATCTCTCCCATTTATGAACACACAGGAAGCAT
GATATGCCATATTTCGGCTATCTTGACATTAGGATAATGTTTTAGACTGA
CTTTAATCCGCTCCCTGCTGGGTTTAGGAAAGCAAGCTGTTTGCTGCTTT
CTACTGCCCTCTGTTGAAACAAAGTGGACAATGTGAGTCTCATCTCTAAG
GACAAAGAGTCAGTTTTCTTCTTCCTTTTTCTGTTTTTGGCTGTACCCAC
AGCATGTGGAAGTTCCAATGGCAGGAACTGAACCTGGACCACAGCAGTAA
CCGGAAACAGCAGTGACAAAGCTATATTCTTAACATGCCATGCCACAGTG
GAATTCACTTTTTGGGGGGCGGGGGGGGGCACAAACCCACCACATGTGGA
CCTTCCCAGGCCAGGGATTGAACCCAGGCCACAGAAGTGACCGAGGCCAC
AGCAGTGCAATGCCAGATACTTAACCTCTAGGCCACCAGGGAACTCCACC
CAGTTTTCAAAAAAAAAAAACTAAAAAATATCTAAGTAGTTTAACATAAC
AAGTGGCTAGCTCATGGGTTAGGGGAGTGAGAGTCAGGTTCAGGAGCAAG
AATCTGGGGATGTTCTTTTTTAAGCCACTTGTTCTGCTCTTCTGCTTGAG
TGACTCTTAACCTCAGACAGTAGCATCAAAGTCTCACTTTCATTCACTTG
CACTCTGGCTTTAGGCCCTTAGCAGTGAGGACACAATTTGAAGTGGTAGC
AGAAAACTAGGAATTAGATTCCAACATCTAGGGTCTGCAATCAAATGGCT
TAGTACCTCCCCCTCAAGCCTACTTTGTCCTTTAACTGAATCAAGTGCAT
GGTTTCCAGCAAAGGTAAAGATTCAGAAGGGTAAGAACACTATCTTATGC
TAAATATGCTGAGAATTGCTGGAAACTACTTCTGGGGAGGGCTGAAGCTG
ACCTTCTTAGTAGTAAGCAAATATTTGGTAAGACTATTTGAATAACTATA
AATGAGGTTCTAAGTGATTCAAAATTATCTTCTTGTCACTGAAAATAGAC
CTACCTCAAGGCCTCAGCAAATATAGGAATCACTTCTCTGGCTTCCATTT
GATTTTCTAAAGCACAGTTCAGCTGGAGACCACCTCTCTAGTTCTGGCCC
ATACCTCTCCTTCCAGGTATCCAGCTCATCCTGACCACGCCCCTGGTATG
TCCCACAGGCACTTCAAACTCATAATATCCACATCTGAACTTATTATATC
TCCCCCCTCTATTCCAGCTAAACTTCTCATCATAGGTCCCACTTCAAGTA
ATAGCACCTCCTAAGCCAGAAATCTAGGTGTCATTGTGGTTTCCTTACTG
TCTTTCATAACCCACATCCCATTTCACCAGATCTCCTTGTTTTTTGTTGT
TGTTTCGGTTTGGTTTTTGTTTTTTGTTTTTAGGGTCATACCCGCGGCAT
ATGGGCTTCCCAGGCTAGGGGTCCAATAGGAGCTACAGCTGCCGGCCTAC
GCCAGAGCCACAGCAATGCCAGATCCAAGCTGCATCTGCGACCTACACCA
CAGGTCACGGCAATGCCAGATCCTTAACCAACTGAGCGAGGCCAGGGATC
AAACCCATAGTTCCTAGTCGGATTCATTTCCACTGTGCCATGATGGGAAC
TCCCAGATCTCCTTGTTAAATATCTAAACCCCATGACTATTACCTTAGTC
CCAAGCTGTCTTCATCTGCCACCAAGACTATTGCAAGAGCCTAACTTTGT
CTTTCTGCTCAACTTGTTTATAAGCTGCAACTAGAACAATCTTCTTTAAG
TACAAATTAGATTTTTTTACTTTCCTACTTAAAATAATTCACTGGTTCCC
CATGCTTCAGGACAAAGCCCAAACTCCTTAAGGTGGTCTATAAGGCATTG
TGGTACAAAGACCCACATATTCCTTTGCAGATTTATCTTGCTACACTACT
CTCCTTATGTTCAATATCAAACTAACTCAAACCTGGGGAAGGGGGGATGC
CGTTGTGGCACAGTGGAAACAAATGCAACTAGTGTCCATGAGGATGCAAG
TTCAATCCCTGGCCTGGCTCAGCGGGTCAGGAATCCAGCATTGCCATGAG
CTGTGGTGTAGGTGGCAGACTGAGTTCAGATCCTGAGTTGCTATTGCTGT
AATGGAAGCTGGCAGCTGTAGCTCCGATTGGACCCCTAGCCTGGGAATGT

GCTGCAAGTGCGGCCCTAAAAAGCAAAAAATAAATAAATAATAAAGTAGG
GAAATTTTTTTGGTCCCAGTGTAGATATTGCCTTCTCTGGGAAGCCTGTG
CTGGCTTCCTAAGGTTGAACAGTTTGAGAATGTTCAGTCATTTCTATCCA
AATGTGCTTCTGCCTTAAATTTAAGTCCTAATCTGAAAAGAAGTATACAT
GGGGAAAAGTTAATAATATGAAAAAAATGGTACAAGCACATCATATTTCT
TTATAACCTCTTTTATATCTTTTCCAGGGGAGGGGTTCCTAAGGAGGGTT
AAAAAGTGGAAGCTAGGAGTTCCCACTCATGGCTCAGTGGTTAACGAATC
CAACTAGGAACCATAAGGTTGGGGTTCGAGCCTTGGCCTTGTTCAGTGGG
TTGAGCATCCGGTGTTGCCGAGAGCTGTGGTGTAGGTCGCAGACGCGGCT
TAGATCCCGCATTGCTGTGGTTGTGGTATAGGCTGGCAGCTACAGCTCTG
ATTAAATCCCTGGCCTGGGAACCTCCATATGCCCCAGGTGTGGCCCTAGA
AAAAAAAGACAAAAAGACAAATAAATAAATAAATAAATAAAAAGTGGAAA
ACTAAATTTCTACAACTCCACAACCAAAAAAATAAGTTACCCAATTTAAA
AATGGGAAAAGGACTTAAATAGACAATTCTCCAAAAAAGATATAAAAATG
GAGTTCCTGCCGTGGCACAGTAGGTTAAGAGACTACAGCAGCTCTGGTTG
CTGCAGAAGCATGGGTTCAATCCATATGTCGCTGGTGCAGCCATTAAAAA
AAATTAAAGACCAACAAGTATATTAAAAGATTTCAACATCACTAGTAATT
AGGGAAATACAATCAAAACCACAGTGAGGTATTACATCACACCTATTAGG
ATGATCACTATAGAAAATTAACAAGTGCTGGCAACAATGCAGAGAAATTG
GAACACTTGTGCACTGCTGGTGGGAATGTAAATGGTGACACTTTTATAGA
AACAGTATAGAGGTTCCTCAAAATATTGCTACCATATGATCCAGCAATCT
TGCTCCTGGGTATATATGCAAAATAATTCAAAGCAGGATTTTAAGAGATA
TCTGCACTTCCATGTTCATTTCAGCATTATTCACAATAGCCAATATATGG
AAACAACGCTAATGGCCACTAAAAGATGAATAGATAAAGAAAATATGGCA
TGTATACACAATAGAATAGTATGCAGCCTTTAAAAAGAGGGAAATTGGAG
TACCCACTGTGGTACAATGGGATGGGTGGCATCTCTGGAGTGCTGGGACA
CAAGTTTGATCCCTGGCCTGGCACAGTGAATTAAGGATCTGGTGTAGGCC
ACAACTGCAGTTGGTTCTGATCCCTGGCCTGGGAACTCCCATATGCCAAG
GGGCAGCCAAAAAAAATAAAAAGGGGGGAGAGGAGAATCTTCTCACATGG
TATACCATGGAGGGTTGAACCTCAAGGACATTATACTAAGCAAAATAAGC
CAGTCACAAAAGAACAACTATTGTATGATTTCATTCATAGGATATAGCTG
AAGCAGCCAAAACCATAGAATCAGAAAATTAAAAGGTGTTTGCCAAGGAG
TTCCCATTGTGGCTCAGTGGGTTAAGAACCTGACATAGTGTCCATAAGGA
TGCAGGTACAATCCCTGGCCTTGCTCAGTGGCTTAAGAACCCAGTGTTGC
TGCAAGCTGTGGCATAGGTCACAGATGCGGCTCAGATCCAGTGTTTCCAT
GATTGCGGCATAGACCTGATTTGACCCCTAATCCAGAAACATGCACGTGC
CACAGGTGCAGCCATTTAAAAAAAAAAAAAAAAGGTGGTTGCCAAAGGCTT
GGAATAGGGGAGAGGAAGAATTAGTGTTTAATGGGTATGGACATTCAGTT
TTGCAGGATGAAAAAGTTCTAGAGATCTGCTATACAGCAATTTGAATAGA
CAACCCTACTGAACTGCACACTTTAAAATGATTAAGTTAGTAAATTTAAT
AGGTTTTACTCACTAAATTTTTTTAAGTAGGGAGCTAAGTGGCAGGAACT
TGGAGGTATCAATAAGCCCCTTTTAATAAGTCAAGTTGTTAGGCAGATAA
CTCCTCTCAGAAAAACACACAAATACATTTCAGAAGGACCATAGCTATAG
AAACTAGAAGAATCAAGCTATAGAAAACCCAGAATTCAATAGAACTGAAT
ATTGATCAAATTCTCTGAAGGAATAATGATTTCTAATTTTAGGGGGAAAA
TACAAAGGAAACAACCCTCACAAAACTTTTACAGTTCTGAATAATGCAAA
ACAGATATTGATGCTGGAAAGATGTAACCAGAATCCGAGTTCTTGTTCAC
GCAGTCAAAGAATGAACTCCATGAACACACAGGCATCAAGCAAGCAAAGT
CTATATTACAGAAAAGCAAATAGCTCCCAGGGCTGCTGGGAGGGAGGAGA
AGAGCCCCCACTCTCTATTGTCTTACAGGGGTTTATATCCCTTAAATATG
GGGAGGTACCAACGTGGGGTCCAGAAAGATGTGGTTTTCTCCAACTGGCC
TTGCCCAATTACCTATATTAGTCCTTGTCCAATAAGGGTTTTAGGGGTGG
AAATTTCCCATAAGTCTCATAGTTTCTTTGCCCTTTTCTTCCCATAAACT
GAGTCACAGGGGATTAGGGATTAATGTCCACCTGGGCATAGGTACTCCCT
ATAGTAAACAAGGCCTGTGGGGATGTCACTCCCCAGAGCTCTTAAGCCAC
AAATGTTAATCAATAGCCATATCAAAGGATGCATCGAAGTCCATGCTCCT
ATACTGACTACCTGAGTTAATATTCTGCCTCAAAATCAACCCAAAATAAA
CAATAGATTGTAAACCGGAAAAAAAATGTTTTTTAATTATGCCTCACCAA
AAGTGATATTTTTTATTTTACAAGAAAAACATTGAGTCCTGGTAAATAGG
CAAAGGCAGGAATATCAACAAAGAAATTCCAAAAAATTAGGTTAAACAAA
CAGTAAATGACCAAGTGTGACAGGCACTGTGCTAAGTGCTTTCCACCTAA

TAGAAATATGAAAGAGGAAATGTGCTAGTAAACAAACACACAGGAAATGT
TCAGACACACTAGAAACCAAAGAAATGCAAATTAAAATGATGGGACCCCA
TTTCTCATCTATTAAATTACATAAGTCTAGGAAAAAGAATGTGGTTAAGG
GGATTCTCACAGATGGAATGTAAACTAAAACAATTGTTTTGGAAGGAAAA
GTGGAATACTATCAAACGTTAAGAAATATGCATGCCAGAGTTCCCGTCAT
GGCGCAGTGGTTAATGAATCCGACTAGGAGCCATGAGGTTGCGGGTTCAG
TCCCTGCCCTTGCTCAGTGGGTTAAGGATCCGGCGTTGCCGTGAGCTGTG
GTGTAGGTTGCAGACGCGGCTCGGATCCCGTGTTGCTGTGGCTCTGGCGT
AGGCCGGTGGCTGCAGCTCCGATTCGACCCCTAGCCTGGGAACCTCCATA
TGCCGCGGGAGCGGCCCAAGAAATAGCAACAACAACAAAAAAAAAGAAAA
AAAGAAATATGCACGCCTATTACATTGTAATTTCACTTCTGGGAAATATG
ATGAAATACATACTTAAAAAAACACTATTACTCTTTGTGGCACCATTGAA
AATAATGAAAATTAGAGACAGCCTAACCATGTCCAAAATCTGACTTTAGG
TAAATTGCTCTGCAACTACATAATCACCTGGAATCTCCCTGAGAGAGGTA
TATGTATCAGTTAAATGACTTTCTGCAATTAGTACCAGAAAATTCCTCTC
AGATGGCTTAAACAACAAGAAATATTTTGCTATCTCACATATTTTAGTTG
TCTGAAAATAGGGCAGTTTCAACGTTGGTTAATCTATAACTCAATAATAT
CACCAGTCACCCAGATTTTTCTCTCTTTGCTTTGCTATTATTTGCATGTG
AGCCACTTTCCTCATGGAAGCAAAATCCCTACAGCTGCTCCTGGCATCAC
ATCCTTAAACACCATCATGTTCAAAGACTGACAGAGAATTCATTACTTCT
TATGCCTCAGGATCTCCACCCCCTGAAAGTAGATTGCCCCTGAGTTTTTG
TTGACCAGAATTGTATCACTTATTCAGTCTTAAGTCAATCATTGGCCAGG
GAATTGGTTCTCAAAGTGTGATCTGTAGCGTCAGCATCGTAATCTGTTAG
AAATACATTTCTGAGGTCCCAACAGAATCGAAAATCTGAGAGTGAGGCCC
ATGGATTTGTATTTTGACAAGCCCTCCAGCTGACTCTGATTTGAGAGTCA
GAGTCAAATTTGAGAACCACGGACTTAGAGGAACCAAAAGCCACCTCCTG
GGGTTGTGAAAAGTTCTAGCCTTCTCTAATACTCATGGCCACCGTACATC
TGAACAAAATCGTGGTCTGTCTGCAAAGAGGAAGGGGAGAAATGGCTGTG
AGGTAGGCAACCTCTGTATAGGAGAAAGCCCTTTTCCAGTGCTGATAATG
AGTCTTTTAAGGATACAAGAACATCAAAGGAGTAATACTATCTCTTCCTT
TCTACACATTTCTCAAAAATAATTTTTTTTTCAATTAAAAAATTTTCATG
TGTTCAAGCCCCAGCCTGATGCTGTGGGCTAAAGATCTGACATTGCTTCA
GCTGTGGCATAGGTTGCAGCTGTGACTCAGATTCTGTCCCTGGCCTGGGA
ACTTCCATATGCCTCGGGTGCAGCAAAAAAAAAAAAAAAAAAAAAAAGGAA
AATATTGAGTGGTGTGTGCAAAAATGCACTTCCTTTGTGTATGTGAAGTA
ATTATAAAAAAGCAAGTTGCAGAATAATCGTGTAAGGTAATATGTAAATA
ATAACAAAAGTGAATAAATATAAATATGTAAAAGTAGGTGTGTGCTTATA
TGGAATATAAATATGTAAAAGTAGGTGTGTGCTTATTTGTATAGGGAAAG
TTCTGGAAGAACGACAAGCTTTTCAGTGGTTATCCTTGAAGAAGGGAATA
AAATTTGGGGAGGGAGGTATCATTTTCATTTTTACTTTATTTCTTCTGAA
TTATTTCACTTACTTAGAGAAAGAATATATTATAGAACATGTGTGTATCT
TCCAACAAAAAATTGTTTAAAGCACCAACGCAAAATACAGGTGAGAGGAA
CAAGGACTGAATCTCTAGGAATGGTTCTGAGCTCTGCTTATATTCCCACC
GATGAAAGGATAGGACTAATCAATGAAATAAAACAGAACATTATTTTCCA
TTTCCTTTTCAAAACTAAGTTTTCTTTTGGTGTCACCATTATTTTTCTAG
TAGGTTCTCATTGGTTTGCATGGAAGAGAAAGCATAAGTTGGGTTTTGGG
GGAAGGGGCTGTTTTTGGTTTTTGTTTTGAAGGTTAAAGTTTTATTGGGG
CAAAATTAGGACTTAGGCTGGGGAGACAGCATCTCAGGTAGCCCTGAGAA
ACCTCTCCCTCAATTTTTATAATTAACTTCCAATTCTTAGAGCTATTAAT
TTTCTAGTTCTGCTAGACAATTCCTTGGACTCCCCGAAATAGCCCAATAG
CCTTCAATAGGATTTTGCCTTTTAAAATTTTTGCTAATTTTAAAAGGGAA
AATTTACTAGTTTTTACAATGTCTAGAATTTATAATTGGAAGTCAGCATT
CAAAACATAGAATAGTTAAGACTGAAAGAAATAGACATAATCTAGCTCAA
TTACCTCACTTATAGATGAGGCAACAGAGGTTTAGGAAAGAGAAGTGCTT
CGACAACTGATCTCTATCAGAATGAAACCAAAAGTTAGATTTCCTTCCTC
CTTGACTAGTATCTTTCCCATAACACATCACCAACTTAAAATTTTGCAGG
TTTGGGGGGAGTCTAAATCCATACTGATGTGAAATAACAAGATAGGGTAT
TTATATGTTAATCTAGTAGATAGCATCAAATTTCTGGATAGCTCTTACAT
GTTAATGGACTATATTAGTTTAATGATCACAGCTAAAGCACAGCCGTGGT
TTATGGTTACACCACAAAATGTATTTTAATTCCTTATAGTTTTCTTCCTT
TGAGTCTATCCATCTCTTCATATTTTTGGAGTTGTTCTATCATCTTCACT

GTCGTTCTATGTGTTACATGTCTGTTTCCAGAATTCAGTGTGTGCATCTT
GTTCTGTGTGCATTTTCAATCTATGTTTGAGACTCAGTGTTACTTCCGAC
AAGCACCTTTGTCTTTGTTAGTTTTCTCTCTCTCTTTTTTTCTTTTTATG
GCACGTGTAGTATCTGTGACCTATGCTGCAAGCTGTGGCAATGCCAGATC
TTTAACCAATGAGCAAGGCCAGGGATCGAACCCACATTTCTATGTACACT
ATGTCAGGTTCTTAACCTGCTGAGCCTCAATAGGAACTTCAGTTTTCCCT
CATTTTTTAATGAAAAATCCTCCAGATGATTCCAGCCCCCAGCTGTTTGA
TTTTTCCCAAAGGAAGCTTCAGACACTGTGGAGCAAATAGAAGTCATTCC
TACCATGCCCTGAATTCCTGACCCATAGAATCCACGAGAATAATAAAATG
GTGGTTGTATTACATCCTAAGTCCTGGGATGGTTTGTTACACAGCAGTCA
AATAGCTGGAATTCTAGTCCTGGGATGTACCGAAGACAACCAGGCCAGCC
TGCCTCTCACAGTCCCTCTCCTGACTGTGTCTTGGATGACCTCACTGAAC
TTATACCTACCTTCATTCTAGTATTTCCTCAATCTGACTGGGCTGCTTTG
GAGGTGCTGCAGGTGGCATAACCTACCTGCACTCCTGCTCAGAACTCCTC
TCAGCAAGCTAGTCAGAGAGCAGTTGACCTGAATACTACTAGAGAAAATA
TACCAACACTGAGAATTGGCCTGGCTACAAGTCCATAATCTTCAACCTCT
GCTGGGCCCTTGGCATGTTTTAGCAATTGATTTACATGCAAACTGTCAGC
TCCTTCTCCTATCCACCATAATGGCCATTCTAAACCTTCTCTGGTATCCT
TAAGCCCCTACTATCACCTTTACCCCCTCTTTCAACAGATGACTGACTCC
TTCTCCAAGAAAATCAAGGCTATCAGATACAAACTACTTCAGCTTTCTAA
CTCAATATCTGAAAATAAATTTGCATATGCAAATTTCTCACCCCCCTAAT
TTTAGCTCAGACGGAGCAGTGTTCCATATCCAGTTCAGAACCAGGGCTCC
ACCTGTATCTACTCTTTTGTCTTCAGGATCCACACTTTCTTCCTCAGTTA
TCCCCTCCTTGTCATGCATCCACCTTCTCCCACTCTACTGACTTCTTCCC
AAAACCTGTAAACAAGTATAAGTCTTTCACTTAAAACAAAATCTCCCTTT
CTTCCCTTGATCTCCTAGAGTTAATTGTCTTCTGATTCTCTTTTCTTTCT
GTATCACTGAAGGCCCTTGACTTTAAGCAACTGTATCAGAATCTGGATAT
ATTTAGGTTTTTTTAAAGGAATTTATTGGAAAGAAACTGGAGGTGGGTC
AGCTTATCCCACTGTTGTGGTGACTGGAACCCAACCACCTTCCTCCCTTA
TAAGTGATTAAGTGTTACTTATTTAGGATTCAATATCCCAGAAAAGACAA
AGGCCAAGCCTAGACCACATGCCTGCTCTTTGGCTTTCTATATTTGTCAG
TTCAGGTTGCCATAACAAAGTACCACAGACTGGGGAAGTTTAAACCACAG
AAATTTATTTTCTCACTTTTCTAGGGGCTAGAAGTCAGAGATCAAAATGT
TAACAGTTAGTTTTGTTTTGTTTTGTTTTCCCACGGCCTCTCTCTTTGGC
TTGTAGATGGATATCTTCTCCTTGTGTCTTCATATGGTCTTCCCTCTGGG
CATGTCTTTGTCTTAATGCCCCTTTCTTATAGGTATACCAGTAATGTTGG
ATTAGGGTCCACCTTTTTTAAAAATTTTTTTCATTACTCAATAAATTTAT
TACACTTACAGTTGTACAATGATCATCATAGCATTTCCATCCCAAACCCC
CAGCATATCCCCCCCACCTCCCAACCTGTCTCCTTCGGAAACCATAAGTT
TTTCAAAGTCTGTGAGTCAGTATCTGTTCTGCAAAGAAGTTTCATTGGGT
CCTTTTTTCAAGCTCCACATGTCAGTGATAGCATTTGATGTTGGTGTCTC
ACTCTCTGACTGACTTCACTTAGCATGATAATTTCTAGGTCCATCCATGT
TGCTAAAAATGCTGTTATTTCATTCCTTTTGGTGGCTGAGTAATATTCCA
TTGTGTATATGTACCACATCTTCTTGATCCACTCCTCTGGGATTAGGGTC
CACCTTAATGGCCGCAAGTTAATGACTACTTTAAAGGCCCTGTTTCAAAT
ATGGTTACATTCTGAAGTACTAGGGGGTTAGGACTTCAACATATAATTTT
AGAGGAGATATAATTTAACCCATAACACTAGCCCAAGGTGGGGAAAAGGG
GGATCTGACTCCTTTGGTTTCTGTAGTAGAAAGCTATCACCACTTTCTAC
TCAGAATACTCACAGTTAAGTTCCTGTCGTGACTCAGTGGTTAATGAAAA
TGACTAGTATCCTCGAGGATGCGGGTTAGATCTCTGGCCTTGCTCAGTGG
GTTAAGGATCCAGTGTTGCCATGAACTGTAGTGTAGGTCACAGACAAGGC
TCATTTCTGGCATGGCTGTGCCTATGGCATGGGCCTGCAGCTTCAGGAAT
CAACCCCTGGCACCTCCATATGCCATAGGTCATTAAGGAACGAGTGTTGT
CACTGCTGTGGGGAACTTTCATATGCTGTGAGTGAGACCAAAAAAAGAAA
AAAAAAAAAAGAACAAAGCAAACAATGGAAAAGCAGGAGTATTACTCAGC
CATAAAAAAGAACAAAATAATGCCATTTGCAGCAACATGGATGGAACTAG
AGATTCTCATACTAAGTGAAGTCAGAAAGAGAAAGACAAATATCATATGA
TATCACTTATATCTAGAATCTAATGTATGGCACAAATGAACATTTCCACA
GAAAAGAAACTCATGGACTTGGAGAGGAGACTTGTGGTTGCCAATGGGGA
GGGGGAGGGAGTAGGATGGACTGGGAATCTGGAGTTAATAGATGCAAATT
ATCGCCTTTGGAATGGATAAGCAATGAGATCCTGCTGTATAGCACTGGAA

ACTATGATGGAGGATAGTGTTAGAAAAAGAATGTATATATGTATGTGTGA
CTGGATCACTTTGCTGTATAGTAGAAAATTGACAGAACACTGTAAACAAG
CTATAATGGAAAATATAAAAATCATTAAAAAAAAAAAAAAAAAAAAAAAA
AGCAGAGGGAGGGAGTGGATCCTGAATAGCCAAGACAGAGTAATGCTTTC
ACAACAGAAATTCTTAAAAGGAGTATTAACTTACTAAAACACTTTTCATT
TCTACTCAATCTTCAACCAAAATTATCAACATTCTGTTCCCTGACTTCCG
CCCCTGGAATCGTTCAGGCAAAGGTCATGAACAGCTCCCAGACTGCCACA
TTGGAGTAATTTTCTCGAAATGTTCTACAGCTTTTTTTTTTTTTGCTTTT
TAGGGCCACACCCATGGCACCCACAGTTCACACCAATGGCCAGGCATCAA
ACATGCATCCTTCTCATGGATACTGGTCAGATTCATTTCTGCTGTGCAAC
AGCAGGAACTCCTCTGTAGCATTGTATACTGCTGGCTTCTTGCTCCTTGA
AGATCCTTCCTCCCTGGGTTCTCCTGTTCTCTCTCCTTAAGCCCCTCCTA
CCCAATCTCCTTTGTCAGCTACTTTTCCTTCACATACTCTGTCAAAATTG
CTGAATGCTAAGTTTTGTCATTAGTCTTCCCCTCCTTCCTCCCCCTCCCC
TTCCTTTCCCTCACTGTTCCCCTTTTTTCTTACTCAATACTCTTATATCA
GAAAGACTGTATTTGTACTTCATCTTAATATAGGATCTCTGAATGGGCTT
CATATGGAGCTTCTTAAATTATAAGAAAAATGATGTGTATACATGCATTC
CCCCCCCACCCCCGGGATAGCATTTAAAGCTTTTATCAGAAGTTTAAAAC
ATCAGTCATGTTGGTGTCCCCTATTTAGTTTAGATCCTTCTGnnTTTCTT
TCTTTCTTTTTTTTTTTTTTTCCTTTGCTTTTTAGGACCACATTCATGG
CATATGGAGGTTCCCAGGCTAGGGGTCAGAGCTATAGCCACCAGCCTATG
TCAGAGCCACAGCAAGGAGAAATCCAAGCCATGTCTGCAACCTACACCAC
ATCTCACCGCAACACGGGATCCTTGACCTGCTGAGCGAGGCTAGGGATAG
AACCTGAAATCTCATAGTTCCTAGTGGGATTCGTTTCCGCTGAGCCACAT
CGGGAACTCCAGATCCTTCTCAGTTTCAAAGCCATACGTCCAACTGTCCA
GTTGACATCTTCACCAAAAATCACCACCAACCTCCAACTCAATAATGGCA
AAAGCTGAAATCATTATTTCCCCCTCCCAGAAACTGCTCCTCTTCCTTTA
TATCTTATGTCCATGAAATGTGTCACCATCCTCCTCATTACCCAAATCAG
AAAGTCCAGCTTCACCTTGACCCTTCTCCTCCATCACTCCTCGTAGCCAA
TCACTCATCCAAATCCTATCCATTCTACCTTTTTAACATCTCTCCCAGAT
GCCTCCATTGAATTTTCACTTATTCAACTGAGATCTCCTAACTCTCAGCT
AAACTGTTAAAATAACTTCCAAAAATTTCCCTCTACATTCAGCATCTCTC
TCTTCTCCAATCTATTTCTTGGATTATGTCATTCCCTAGCTTAAAACTTT
AATGATCAGATTAACATTTTGnAnnnnnTnTnCnnnnnTTTTTTTTTTTT
nTTTATCTTTTCTAGGGCTGCTCCCGCGGCATATGGAGGGTCCCAGGCTA
GGGGTCGAATCGGAGCTGTAGCTGCCAGCCTACACCACAGCCACAGCAAC
GCAGGATCCAAGCCACATCTGCGACCTACACCACAGCTCATGGGCAACGC
CAGATCCTTAACCCACTGAGCAAGGCCAGGGATCCAACTCGCAACCTCAT
GGTTCCTAGTCAGATTCATTAACCACTGAGCCATGATGGAAACTCCTGTA
AAAATTATTCTGATAGTAGTTCAAGAAAATACAAACTCTTCACCATGACA
TACAAAGTCCTTTATGATCACCCAAAAAATTTTAATTGAGGGTGTATTCC
AGGCACTGTGAAATATACTGGAGATACAGTGATGAACAAGAGGAACAGAT
TTCCTGCCTCTAAGATTTACAGTCCACAAATGTGATGAGTACTGCAAAGG
AGAAAAACGAGTGCCTAAAGGAGTAAGAGGATGGGAGTGTCATAACTTAA
GTCTGAAAACAACGGTAGACCTCAACTTCCCGTGTTGCACTTCTTTTTTT
TTTAATGGCCACACCCACAGCATGTGGAAGTTCACAGGCCAGGGATAGAA
CCCATGCCTCCACAGCnnCCnnnnCCAnTnnnnnCAnnnnCnTTnnnTTT
TTTTTTTTTTTGGTCTTTTTTGTCCTTTTAGGGCCGCACCCACCGTGTAT
GGAAGTTCCCTGGCTAGGGGTCGAATCAGAGCTGTAGCTGCCAGCCTACA
CCACAGCCACAGCAACACCAGATCCAAGCCATGTCTGTGACCTACACCAC
AGCTCACAGCAACGCAGGATCCTAAACCCACTGAGTGAGGCTGGGGATCC
AACCAGCAACCTTATTGTTCCTAGTCGGATTCGTTTCTGCTGTGCCATGA
CGGGAACTCCACAAGTCAGATTCTTAATCAACTGTCCACAGCAGGAACTC
CTGCATTGCACTTCTAATAGTCATCAACAGATATGGTTCCAGATACACAA
CCAACAACTTTTGGGTTAGTGCCATTCTTCTTATCCCTATTTTATAGATA
TGGAAACTGAGGTCCTAGACAGGTTAAGCAACTGGTAGACTTGGAACTCC
ATCCCAGGCTCATCTGAAACCCAGTCCCCTTAACCCTTTAGGAGCTTGTG
CTTCTCCTTATACTCAGGAATCCTGTGGAGCTATACCAATCTCTGTAGAC
ATGCCAGCCGACTTCACTCCAAGTCTTTATTCATGCTGCTCTTTTTGCCT
GAAATnnnnnnTnCnnTnAnnnnnnnTCnnACGCAATTAAAGTGTTCTCT
CTTCCACATGGACTCTTCCTCCGGGGAAGAATTAGCCACTACTGCCTTCT

TTGTGCATACACAGAGCTTCACAAACACTTGTAGAGTCCCTATAGCACTT
CAGATACTTCTCTTGTAGTCTCCCTCTGAGAAACTATAAATCCCCAGGGA
TGATAACTGATCAACCTCTGTAATCTCAGAATCTGTGCTGTAGCTGTCAT
TGCAAAGTTAGGGAAGAGAAGAAGGGAGGGAGCAAAGAAAAGGATACGGC
AGAAAAGAATAAGCAAGCATGAAGACAAACACTCCAATATAAGATATGCC
AAGACCATCTGTCCTTCTTTCAAAAAGGTGCATTTTACCTCTCACAGTAC
AATTAACCCATACCACCTCCACAACGTTCAATTTGAGGATTGTTCGATAA
GTGTTTCATGAAACCCAGGAGAATGTTTCCCACTCTTTTGCAGTCCTCCT
GCGCTCTCCCTCCTCTTTCCTGTCTATCCCATCCACTCTGCAGCTGACAG
TTCCTTCCTCCATGTGAAGGTCACTGACAGAAAATGGGTCAAGAAGAAAA
TACAACCTTGAAGAAGGAAAAATAAAGTGAACGCCTTGTACCATATCGGG
TGGGAGAAAGAAACTTTGGTAAAAATGAACAACAGAACATGCTGAGTGAT
AGCCACCTTTCACCTCAGAAACCACAGAAAGCCCGGAAGGCACGCTTTCT
GCTTTAGGTGTCCCAGACAAGCACCTGTAGGGCTGTAGCATGGAGCCTGA
AGTTTTCAGATGCTACGCTGGATTTTGCAAGATGAAAATAAATCACTTGC
GAAGTAGCACTAGAATGGCTGCTTTGTTGGTTTCTATTGTCCATCCTAAC
CAGACATCTCATCCAGGAACCTGTGGAGGAGGGGCTGCTGCAGGAACATA
TGGGGCAGGGGAGGATGGGAATGGGAGAGGAGCAATTAACTCTACTCAGG
AGCAAAATAAAACAGAAATTAAAACTGTTAAGTGGTTTTTATATGACTCT
GGAGTGGAAAGGGGTGTGGGTCCACTTGTGCCAGCCCCTGCCTCCACTCT
ACCTGCCCACTTCTGTCTGCTGCTCACTTCCTGGGCTTTTTCAAGGCTTT
TCCAAATGAATCCTGTCTCTGATGTCCAGCAACCTGCTGAGGGCGGGATA
TGGCCAAGCTGAGCCATTTGACTTTCTGTCTGAGACCTAGACTGATGTGA
GAATAAACAAAGTGGGGGTAGGGGTGGGGAGTGTATGGGTATGTATGTTT
CAGCAGGGGGGGATGTAGAGGAAGGAAATTAGCAGTGTCACCACTTCCTAA
CCATTGCACCTCCAACTTTTCTGGAAAGGAGCCCTGGACCCTGGGCTGCT
AGGCTCACAGACCAACCCAGGTCCAGTGAGTCCCTGAGGGGCCCAAGCTA
ACATGAGCTACTGCAGCCCAGCATAGCTCCCATAACACTGGGGTGAAGGT
AAAGTGTTGGTGCTCAACTCACCCTGACCCTTTCCCTCCTCTCCAACTTT
TCTGCATTCCTTCCCACCTTTCAGTGGTATCTCCTCCTCCCAGAGCCCGT
TTCTCTCCCACTGCCTGTGTTTCTTTAACAGACGATGCAAATCACATGAT
TGAATTCATACTAAGAAGTTAGTAGCTCCTGCGGATTACAGATCCCAGGG
GCATATGCATTTTGAATTTTGAAAAGAAACATAGATGGAGATAATTTTGG
GATGAGGGACACCTCTAATGATCTGAAGATTGAAATGACTACTTTCTGTA
GGCAGACCTGGCTGGATGGAGAAGGGTCTGGGCCAAGCAGGAGAGTGAGC
AGTTCTGAAGCAGAACAAGAGATTCCTTGAGGAAGGTTCTCCCGCTTGAG
TCTCATTCCCAGGGTCACATTGTCTTGTTCATGGCACTAGGTGGATCCAA
ACCCAAGAGGTGGGGGCTCATTTATCAGAGTTCAGATTCAGGAATCAGAT
GATGTTTCCCGAGAAGGAAACTGGTGAGTAATAGGCTGGGGGTTGGGGGA
AAGGCTGTTTACTTCCTCTGTTCAAGTCAGTGAGGTCCCTTTAGAGCTCT
GGATAGCTGTGGTGACGGGTTCACGCTAATGCACAGCATGTGGCAGCTGC
TGTCACCAACGGCTCTGCTGCTTCTGG**GT**AAGTCAGAATTACCCTGGCTG
GGAGAGGAGCTCAAGATGCCCTGGGTTCAGCAAAGATCCCTTTTCAGGCC
AGGAATGAGACCCCTACCAAGGAATAGAAGCCCTGACCGTGTCTATAGAC
CTAAATTTAGCTCCTAGGACAAAGCTTTGAGGGTTAGAGATGCAGAGGAT
GCTGGCTCTGATATGGGCATAATCAGGAGGTGAAGGGAGATAGGAGGTCC
TTGTTACATCACTCCTCTGGGTCTCAGTTTTCTTATCTGTACATTGCAGA
TTATGGACTACATGGTTCCTACGGTCTGTCTCTTCCGTTCTCACATTCCA
GTGTTCCCCTCTAAGGCTTTTAATTTCTACCCTTTCCAGGGGACTTGGGT
TGAGGCTGTTTCTATGGCTTGATTCAGGGATAGTCGATTTGTACCCTTAA
TTGGAAAGTTTAAAACAGGACTGAGGAGTAAGGGAATGGTGTTGAAAACA
GAGGTTTGGGTTATAAAACAGAGGATGGAGTAGTAAGCACAGACATCAGA
GACCAAAGGGCAGACAAAGGGAAAGATGTCCACTATATTTATCTTGATTC
TTGGGAACACTGGCCTCCCAGGGCAGAGGAGGGCAGAGGAGTTGAAACTC
ACTATACGTTCCAGCCACCCCAATTTCTATCGCTGCTCAATATTTGTTTC
ACTCTTTTTTTCTTAC**AG**TTTCAGCTGGCACACAAGCTG**GT**AAGTTTGCT
TCATGGTCTTGGATTGATCAAATGAGTATGTAAATATATAATCGCAACAG
GGTGTTGAGAAATGTTTTTTGCGCGGGAAACTGCTGATGCAGTTATTAGC
GAGAGCAGTTCCCCCAAAACAGTGGGGCCTGGTCCTATGCTTCCCTAACC
AATTTTCCACAAGATAAAGAGAGGCCAAGTGTCTCCAGCCAAGATGCTCA
GACTGAGTCCCATCCTAAGGCTGAGTTGTCAATTCTGGGTTTGTGATGGG

CTTTGATCCTCCCCAGGAATAAAAAAAAAGGAATGCCAATGCAGGGCCTG
CTCATTTCCCTGCCCTTACCTATATTCTCTTGACTCCTC**AG**AAGATCCCC
CGAAGTCTGTGGTGATCCTAGACCCTCCATGGGACCGGTTGCTTGAGAAG
GATTCTGTGACTCTGAAGTGCCAGGGGGCCTACCCTCCTGGAGACGATTC
CACAGAGTGGAGGTGGAATGGGACCCTCATCTCAAACAAGGCCTCCAGCT
ACTCAATCACAGATGCTACAGTTGGGAACAGTGGAGAATACACGTGTAAG
ACAGGCCTCTCTGCACAGAGTGACCCATTGCGACTAGAAGTCTACAAGG**G**
TGAGTAGGTGAAGGGGAAGGGGGAAATTACCAATAAAGGGTGAAACAAGA
GGCCCTGAAAGATTTGGGAAGAGCCAGAAGATGTTCTTGGGGGTAAAAAT
TAAGATGAGATGATCTGTAGCTCCAATGAATGATATCAGAATTGCAGTCC
TAGCTACAAGTAGGCCTGAGGTAAAATGTCAAAACAGGGTACATACTAGA
TACAAGGCTGTTGCACCATACAATTCCAGGAAGCAACATTCACAGGGAAT
ACATATGGCATGAATGGTGTCATCTGAAGGTTAAGGCATGACATAGATGC
TTGATAACAGTTATTGAATAAATTAATAAATCCACTTTGAGGACCTATCT
CCTCACTCTCATACACTCAATGCCCTAAGCAACCCCCCAAGCCATTCTTG
CGTTTTGTTTTGTTTGTTTTGTTTTGTTTTGTTTCTTTGGGCCGCATATG
GAGGTTCCCATGCTAGGGATCAAATCAGAGCTGTAGCTGCCAGCCTACGC
CAGAGCCACAGCAATGCAGGATCCGAGCGACATCTGAGACCTACACCACA
GCTCACCACAACAACGGATCCTTAACCCACTGAACAAGGCCAGGGATTGA
ACCTGCATCCTCATGGATGCTAGTCAGATTTGTTAACCACTGAGCCACAA
TGGGAACTCCCCCTCACCCCCATCCCCCAAGTCACTCTTAATCTAGCATC
ATATCTATCTCTTTCCGTCAATGCCATATTCACAGGTACACTTCAGCATA
AATTTGGCAATATGATATCTCTCCTAATAGAGATATATACATGTTCAGCC
TGCACATTCATTGTAAAATGAAAATCTTTGTGTATTAATAGAATCCAGGT
ATTAGATGCAAGTACAGGAAGTATTTTAGAGACAGACACTCTCCAGGAGT
CCCTGTAATCACACCACATAGCCTGGTATGTGCAGTGCCATCTTGAAAGC
CTGGTTCTACTTCCATGGCTGCTCTATCTGTACAGTGTATGTGGGTGGAA
CAAGCCTGGAGGGGAAGGAAAGCCTAATGAGGGTGGACCTACAACACAAC
TCCATAGAATTGGGCCTCTGAAACTGGTTTTCTTGGCCTCCTTTGCCTGA
CTGTGTCACTTAGCCATGGTTCTCACTCTACAGAGCTACAAAGTGGAACG
TCTATCATTTAACTAGGTGAGAGATAGTCACCTCTGACCAAATAAAATCA
GGAATCATCAATAGGAAGCAATAGAAAAATCGAGTCTAGAAGTTATTAGT
TTTTCTTAAACTTGGCTCCAGAAACTGACTTTAGAAGTCTAACCAGTGAG
AACTGTACTAAACTGTGACCAAGGCCATTACTTTCACCAGGCAAACTCCT
GATCATCCCATGGGACCCATTGCACTTGTTTCCTTTTCTGAGAAGCCATC
CTTCCTTTTTTTGGGCAGAATTTCTTTGCTGTATTCTCTTTGGTCCTGCA
CGCTTTTGTTCTTTCCCTGTTGTAGCACATACTACATTGTATTGTAATTA
TTTCTAAGTATGTGTGTTTCTATGTCTAGGCCACAGATTCTTTGATGTTA
TGGATCATGTGTCTTAGTCATCTGGGTATCCCCAGTCTGCACAACACAGT
GATGGCTCAATAAAATATCTGTTGAATGAATGTACATATAGATATTCATT
GAGTTCATGACAGCCAGAATGGAGTAAGACTGTTCCCCCCAAAGCCACCA
GAAAATTGTACATATTACATTATATAATATGTAATTATATATAATATATA
CATGTAACTCCTGTTAATATTTAAATACATGTTCCATGTTTAGTTTTTTT
TCTTTTGGGTTTTTTTTTTTTTTTTnGAGnATATATACTTTTTGAATTCT
GTCTGGTTTTTTTTGGCAACCTGCAACTATAGAGTTCCCAAGCCAAAGAT
CTGAGCCACCGTTGCAGCCTATGCTACAGCTGCTGCAACTTAGAATCCTT
AATCCACTGGGCAGGGCCGGCCAGGCTGGGGATCAAACCTATGTCCCAGG
GCTCCAAAGCTGTTGCCAATCCCGTTGCACTACAGCAGAAACTCCTGTAT
TCTGTCTTTTTAATTTCACATTGTCCCATGAATGTTTTCTTGTGTCATTT
TAAAATTTCTCTGTGTCTGGAGTCTGGTAACTTTGCAAGGGAATAGTGAA
AACAAGTATCTTGGTGATATAAAATTTTGTCTGTTAAAGGCTATGCTCGG
TAATGAAGATGGCCTGGTGGAGATGCTGTGGCCCTCAGAAACCACCAATT
ATGACCTGCTTTTCTTTCCCCTCCTTTATTTATTTATTTATTCTTTTTTT
TnnTTTTTTTTTTTnnnnnnTTTGCCATTTTCTGGGGCCGCTCCCGCGGC
ATATGGAGGTTCCCAGGCTAGGAGTCGAATCGGAGCTATAGCCGTTGGCC
TATGCTACAGCCACAGTGGGATCCGAGCCTCGTCTGCGACCTACACCACA
GCTCATGGCAATGCCGGAGCCTTAACCCATTGAGCAAGGCCAGGGATCGA
ACCTGCAACCTCATGGTTCCTAGTTAGATTCATTAACCACTGAGCCACGA
CAGGAACTCCCCTCCCTGACTTTTATATGTTCTCCTTCAGGATTTCTGCC
ACCACTCCTGTGGCCACTCAGTGACCTTCTTCCTGACTGGCTATGACAGC
ATTCCAACATTCCTACAAGTGTCTCTTCTGAAGTCTGACTCTCCTTTAAT

TGGGAAAATCATCATTTTTTTTAATTGAAGTATAGTTGATTTACAATGTT
GTATTAATTTCTACCATAAAGCAAAGTGATTCAGTTGTATATATATAATT
GAATATATATGTATGTATATATATGTATATATACATATATTTTTTAATAT
TCTTTTCCATTATGGTTTATCATAGGATATTGGATACAGTGCTGTACAGT
AGGACCTTGTTCATCTATTTAATGTATAATAGCTTATATCTGCTAACCCC
AACCTTTCATTCCATCCCTCCCTCAACCCCTCACCCTTAGCAACAACAAA
GTCTATTCTCCTTGTCCTTGAGTCTGTTTGGTGTCTCATAGATAGGTTCA
TTTGTGTCATATTTTATTTTTTATTTATGGCTGCACCTGTGGCACGTGGG
AGTTCACGGGCCAGGGATCAAACCCATGCCGCAGTAGCAACCCCAGCCAC
CACAGTGACAAGGCTGGATCCTTAACCTGATGCTCCACAAGGGAACTCCT
GTGTCATATTTTAGGTTTCACATATAAGTGATATCATATGGTATTTGTCT
TTTTCTTCCTTACTTCATTTAGCATGATAATCTCTAATTGCATCCATGTT
GCTGCAAACATTCATTGTTTTTTATGGCTGAATAATATTCCATTGTATAG
ATGTACCACATCTTCTTTATCCATTCAACTGTTGATGGACCTTCAAGTTG
TTTCTATGTCTTGGCTATTGTGACTAGTGCTGCCATGAACATAGGGGTGT
ATGTATCTTTTTGAGCAAATTATCATTTTTCTAATATAACTTTCACTCTT
CGATCTAGAACTTATAGTGACTCCTTATTACCTGCCATAGCAAATCCAAG
TGTAGCACTTAGGGATCCTGTAGATTGCTTCTCAACTGCCTCATCACTTA
ATCCCTCTGCTCTAACCTTCTTTTGTTCATGTGTTCGTCAGTCCCTCTCA
CTCAACAAAAGTTCTTGATGTGACTTGAATTTTCTTTCAGGGCCCAGTTG
CCTTTTTCCCAAAGAAAGCTATGTAAGGAAAAGTCCCAGTCTGGCTTTTC
ATTATGCCCCAGGATCTAGGTAATAATCATCCCCTTTACAAGAGCTTCAC
ACTGAATGTTGAGGCCTCCTGGACCTGTGGTCTCGTTGTGTCTTTC**AG**GC
TGGCTGTTGCTCCAGGCTCCTCGGTGGGTGGTCCAGGAGGGGGAGTCCAT
TAGGCTGAGGTGCCACACCTGGAAGAACATTACAATACAAAAGGTCCAAT
ATTTCCAGAATGGCATGGGGAAGAAGTTTTCTCATCAGAATTTTGAATAC
CACATTCCAAATGCAACACTCAAAGATGGTGGCTCCTACTTCTGCAGAGG
AATTATCAAGAATTACAATTTGTCTTCGGAGGCTGTGAAAGTCACTGTTC
AAG**GT**AAGAGGGTGCACCTAGAGTCCTCAGAGGCCTGTGGCGGGAGGGGG
AAGTTTCATCCACACTGGGAAGATGAGTTGGGAGGACATTGTTGACCATT
CCACAGACTTCTTTACATGGGCCATTCACTCTGCAGCGTGATAGCAGGTG
CAGAAGCCACATTCCTCCCCCAAAAATATGTTTGTCCTTACCTCATTCAA
ACCATATCCACAATGTTATGTGTCTCTATTATACTGGCTCATCTTTACCT
GCCCTCTTTTCCCCTCCAAGCCTCAAGTCTTAGCCAAGAGCCAGGCTnAC
AATAGCTCTTCACTTTCTCACAAAAAGAGCCTGGAACCTGAATTCTGGCT
CTGCTGTTAATTACCTGCATGACCGTGGCCACTTAGTGTCTCTGAACCTC
AATTTCCACTTCTGTAACAAAGACAAGCAGACATTTATTGGGTAGCTTTC
AGGATTCTGGACACTGCTAGGTGCTTCTTTACATGTAACTAGTTTGATGA
TCATAGTAACCCCTCACAACAACATTAAGAGATAGGTAATATTGATTCTA
CTGATGAGTTAACTGAGGTGGAAAAAAGGGGGATAATTAATTTGTCACCA
AGGTCATACTCAAGTTCAAAGCCTGTATATTTAATCAATAGATAAAATGA
AAnGCTTGGACAGGCGCCAATGTCCTGCGACAGAAATGAATCCCACTAAG
TCACTTTAAAAACATTGCCAGTTTCTGTAGTGCCATCTTTCAGGGTCAGA
GATCCCCTACTACAGTGCTCCTGGACTTCCCAATTTCATAAAACCTCCAC
CTGCAATCAGACTAAGATGTACATTGTAAGCAATTTACATGTAGTAAGAY
AGCAAGATAGACACACATCTTTTGAGACTTTAACAAAATAATGCCATAAT
TTGTATAAAAAATAACTTCATGACAATTTAAATTGAAAGTAAATACTCTC
AAATAGGGAAAGAGAGAGCAAATAATAAAGCAAAGGGGGATACAATGCCA
ACAGTTAGATAATCTAGGTAACGGGGTTCCCATCGTGGCTCAGTGGTTAA
CGAATCCGACTAGGAACCATGAGGTTGCGGGTTCGGTCCCTGCCCTTGCT
CAGTGGGTTAACGATCTGGCGTTGCCGTGAGCTGTGGTGTAGGTTGCAGA
CATGGCTCAGATCCCGCATTGCTGCGACTGTGGTGTAGGCTGGCAACTAC
AGCTCCGATTTGACCCCTAGCCTGGGACCATATGCCACGGGTGAAGCCCT
AAAAAGCAAAAAGACCAAAAAAAAAAAAAAAAANNNNNNNNNNNNNNNNN
NNNNNNNNNNNNNNNNNNNNNNNNNNNNNNNNNNNNNNNNNNNNNNNNNN
NNNNNNNNNNNNNNNNNNNNNNNNNNNNNNNNNNNTCCATTCTAATGTTACC
AGTTTATAGTCAGCAACATGGAATGATTTTAAAATAACAATATTAAGGGC
TAAGAGGTTGAGAACAAACAAATTCTACCTCTTTCCATTTGGGGTTGTTA
GTTATTGAGCCTCGATAAAGAAATATTATCAATTTTCTGCCAATAAGTAG
CAAGTTTCAATGGTTCACTTTTTTTTAGATTTCCTTTAATGGGGTGGAAA
CAATGAGTACAATTTCGAAACACAAGGGACAGTTACTGAATTAAGAAAGT

GTTTTCACACCAACTTAnACTTCGACAACTTTCATGATTTCACACAAAAC
CCAGCTACAACTTAACTTGTTCAACACTCTCAGTCACATACCTCAATCAA
ACATGAAAACACTTTTGCTCAGGTAGCACTGAGATCCAGACTCAGTGGAG
GAAACAATGGCTTTCCATCAGACTTCCCAACTGTTCCTTAGACCCTGTCT
CTTAGCAGTTGTCAAAAAGACTTTCCACTGATGACCTCACTGTTTATTGC
AAATTTCTGCCTTAACAGATGTCATTTTATTCTCATCTCTCTGCTTCTCC
TATT**AG**GTTCAAAAAGTCCATCACCTATCCCATCATTCTTTCTACCTTGG
CACCAAATCATTTTCTGCCTGGTGATGGGATTCCTGTTTGCAGTGGACAC
GGGGCTGTATTTTTCTGTGCGGAAAGTACTTCGAAGCTCAAAGGAGGACT
GGAGGAATGGCAAAGTCACATGGAGCCGGGACCCGCAGGACAAAGGAGGA
TAACAAAAGCAGCAGCATCACCACCACCACTGCTTTTATTATCCCATGGA
GTAATAAAAGCAGCATCTCTGAACCTGTTTGTTGTTTTTTGTTTGTTGGT
TTTGTTTCTTTAGGGCCATACCTGGGGCATATGGAGGTTTCCTGGCGAGG
GGTCAAATCAGAGCTACAGTTGCTGGCCCACGCCACAGCCACAGCCACAG
CCATACGGGATCTTAGTCACATCTTCGGACACACAGCTCATGGCAATGCC
AGATCCTTAATGCACTGAGCGAGGTGAGGGATCGAACCTCCATCCTCATG
GATACCAGTCAGATTCATTTTCGGTGAGCCACGACAGGAATTCCTGAACC
TCTTTCTAAAATCACTACCCCATCATCCTCAACAGGCATCTCTGGGAGCA
GCAGGAAAATTATACCTCTGTGTCTAGGCTGAGGGTCCTTTACCCAGCTC
TGTTTTCTCTTGGTCTCCTATGGAAAAGAAAGGACTGTGATCTTCAAGTG
CTAATACTGAAGCCCCAGTAAATAGCTGCATTACCAGGAATAGAAGTGTC
AGATAAGTAAATAAGAGAAATTATTGTTGTGAGCCAGCTGGATAGTGGTC
TGCACCTGTTGCTGAAAGGCTGGCTGCCTTCAAGCCACCCTAAAAGTCCT
TCTAGGCAGACCATGGGTATTCCTGGAGGTCTAAGGGAGACCACAGAATC
AATGAAGGAGCTGGCTAAGAAAGAGCAGGGAAGTCCAGGATGGACCAGAG
GAGACCTTCACCTGGGCTGTCTTCCTAGAACATCAGCCTAGAACATGATA
GAGGTAAAAGAAAATCACAGAGGTGGTATAGAATTGACTTTTTCAAGGCA
ACCCCTGTCAGAATTGGGATGGTCTGCAAGTATATGTTAATGAGTATAAA
ATGGTCCAGGAAAGGGATGGGATTGGAGGATTTAGATAAAAAAGCACAAA
ATAAACCTACTGGAAAGAGAGAAACATTGCTGTCCCAGACTAAGCTCAGT
GAAAAAAGATACCTCAGCGTCCAAACTGAGAAAGCATGACAAGAAAAAAT
TCAACCGCAACTGCACAGGAAGGAATGGGCAGGAGCCACAATAGTGAGAA
TTAGAGCGAGAAGGGGCAGGGACTATAAAACCAGCTTCTCTGTTTCAGTA
CCTTATTCAGCACAGCTTTCTTCACATCATCCATTAAACAAATGTTGGAT
AACCAATATTAAATGTACTAAATTCTACTGCGTTAAATTATGAAACTTTG
AAATCCCTCAATATATCAGTTATAATGGAGTGGAAGTGGAGAAGGTGATC
ACTGCTACTGCTTTTGGAGGAAAGCTTTAGCTGTCTCTCTGTTTTAGAAG
CTTTAAACAGCTTTCAACAACATTTCTTGATTCCAATACGGCATTTTACA
AGATCTTGCATGCTACTGTTAGACAGAAGATAGAAAAACCATGGTGATAA
AACTAGAATGATAAAAATCTTTTTCCTCCCATTGTCCAAAATTCTATCAG
AGATTAGGTTGATGAGAAACCCCCTCTCAGCAACCATCTCACCACAACTC
ATATGAATTAGTCAGCTTGCTAATTCACCAAATCTTGCTGGGACAACTAG
GCAAAACAGAACTTTCACAAGTAGTATGGGATAAGAAGGTGAATGGGAAT
TAGAAGCAGTCTTAGAGCATGCGGCAGGTATGAGAAGGCACAGCTGGAAA
GAGGCAGGGTCTGGGCAGAGGTTTAGGGTCTACTTAATTGTGTCAGTTAT
GAGCTTCATGCCAACAAGAGTCAAGCTTGGTGTAGATTGGTATGAGATTC
AGACATGAGACAAGGGCATTTTGGAAACATTGAACATTCAACTCACATTT
ATTTAGTATTTACAATGTGCTATGCTTGAGTTTAGGTGCTGAAGATTTCA
AGGTGAACAGCACAGGGTCATTTCCTGGAGGAACTTTCATTCTAGTGAGG
GGGAAAAAAAAAACAACAACACATAAATAAAAAGAGAATGGGAATTCTCT
TCATGGCTCAGTGGTTAATGAACCCAAGTAGGATCCATGAGGGAGTGGGT
TCCATCCCTGGCCTTGCTCAGTGGGTTAAGGATCCTGCGTTGCTGTGAGC
TGTGGTGTAGGTCAAAAATGCAGATCAGATCCCACGACGCTGTGGCGTAG
GCCTGCAGCTGTAGCTCTGAATCGACCCCTAGCCTGGGAACTTCCATATG
CCGCACCTAAAAAGACAAAAAATAAAAAAGATAATGAAGAAAGAGCTGCA
GAAAAAAGTCTTAATTTTTGGTGTTCTGGTAATGGATGAGTAGGAGTTGT
GAGGCAAAAAAGGAAAAATGATGAACATCCATGGGCAAAGATTCAAAAGT
CAGAGAAGAGAGGTAAAGTTGTTTGCCAGATCATGAAAGGCCTTCAAAGC
TAAGGAGATTGTAGTTTACCCTGCTAGGGCTTGAGGAATTTTAAGCAGAA
CCTGTCTTGAAAGCTTCTCTGGCAGCTACTTGGAGGCTGGAAGAGAGAAG
ACAGTGAAAGACAGGAAACTGCCCAGAGGGCTAGATGGTTATTGCAGTAA

CTTAAGGGCTAAGTAATGAGGAAGTAAATGTCAGCAGGCTGAAGAGGAAG
ATGATTGTTCAAATATTTAGAAGGTTGGCTCTCCAAAAACCAGCAAGACA
TAACATGACCAAGGGCCCAAATTCTGGAACCGCCCGTCTCACAAGTTAGG
AAGTACAAGTACAAAGGCCAGGCAGGTTGAGCAAAAAATTCCTTTTTGGA
TTATGAGTACTTTCTTTTATCGGTAAATACTTCCAAGGCATCCGTTTTCA
TGATGCGGTTTTTGTAGATCCTAAAGAAGAAGTTGTGTCACCAATTGCAG
CCCTAACCAGGCAGATCAATTTTTTAAATGCGGAATGGACAATTGAGAAC
TTCACGGTGTTGGCCGGGTTTAAACAAAAACGGTTGTTTTTCCCGTCCCT
GGGTGGGCTCGAACCACCAACCTTTCGGTTAACAGCCGAACGCGCTAACC
GATTGCGCCACAGAGACAGGCGGCGTATGTATCTCCTGTTTAGTCAGTTC
ACTGAAGATTAAACTCCTAGTGTTCTCATTTCCAAAAAACTGGTTGAGGC
TGGCATGAGAACCGGTGGAGGCAACACCTTCCAACGCATTAGAGAAAGGT
TAGTAGCCCCGAGGAAAACGGGTCTTTTTCCCGTCCTAGAACCTGCTTAG
ATCTAGGACAGGACTCGGTTTCTACCCGGAAGACGTGAAAACGGAAGCAG
CAGAGGATGAATGAACTGAGGTACTTTGGGAAACCCAAAATGAATGCTGT
GGGTTCAGGAGTTCACTTGGTATTGCTCAAAGAAGTACATCGATACACTT
TTTTGCTGAATTAGACAATTTATGCCTTCAGGTGTATAAGTGCGAGTATT
AACTTATAATAAGCCTATTACTGTGAAAATTTTAAAATGCCACATCACAT
GTCCATTTGCCACATGTCTAGCAAAATGTTATAGCTAAGTAAGGGAGCCT
AACGACAAAATTAATCTTTACCCCTAGCTGTTTTGAAAGGTTTCTGCTTG
CATGTGAGGCATCAAAACAGAACAGCGTTTATAAATGCCTGTAATTTAAG
ATCTTCATGTTGACATAATCCAGATCCAAATATCCAAAGGAGGGCCCTTA
TCCAACATAATCGACCTAGAGATCCTGGTCAGGGCTGGGCCAAATAAAGT
TCTCTTCTCTCCAGTTTGGAGAGAAGCTCTTTATTCCTGGGCCATTTAAT
AGCCACAGGCTTCCTGAGTCACATCACCTCCGCTCCCGGCATACCCAACC
GTCTAACCAGGCTGGTTTTTCGCCCCACACAAAAACACATAAGCAGTCAC
TTTCTCTGTTGCTCAATCCCAGGGCGCCACGGAAGCCTCCCCATAGAACC
TCCATTTTCCTTCTGCCCTGCCATCCTTTAATAAGTGGGCTCAAGTTTAG
GTGCAGGTCAAAATTTTCAATGATTTCCAGTGAAATGAGAATACACACAA
AAATAGATATGCATATGCATAAATTATGTGTATGTGTATCTCAGATAAGC
TTGACAAATGACCTCTAAGCTGACATTTTTTTTACACTTTAAATTTTTGT
TTTTAAATGTCCTTTCTTTTATAAAATAATGATGAGGCTGTGATTTTTCT
ATTAATTTCCTAACTTGGCAAAATAAAAACTGACAGCCTTATATTGGTCA
CCAATATTTGGGGGAATTTTTACTTGTCTACAAAGACCAAGTTTGGGAAC
CACTACTCTCACCTTCACCTTCTTTACTGGATTATTAAAAATAATTTGCA
AGAGTTGCTGCTGTGGCACAAGGGGATTGGTAGCATCTCTGCAGCTCCAG
GACAGTTGATAGATCCCTGGCACAGTGGGTTAAAGGATCCAGCTTTGCCG
CAGAGGCAGCATAGATCACAACTGATCCCTGGCCCTGGGAACTCCGTAAG
CCACAGGGTAGCCAAAAGTAACAATAATATTAATAATAATAACTTGCAAG
TTATTCTAAGTACACATACATTTTCCATGTTTCTCATGTATACATGTCAG
CAATCCAAAAGAAAGGAGGAAAAAATAATAAAAGGAAAACTGAGGCGAAA
GAAAGTAAAAAAATAGGGTGGTGAAATAAATCCAAATGTATCAGTAATCA
CAATAAATATAACTGGAGAAAATGTGCCAGTTTTTTAAAGTACATTTTAT
TTTGTTTAGAAGAATGACATTAAAACATAAACATGGAGTTCCCTTGCATC
AGGTTAAGGATCCAGCCTTGTCACTGCACTGGGTTGCTGCTGTAGTGCGA
GTTGGAACCCTGGCCTAGGAACTTCCACATGCCACAAGCAGGGCCAACAA
AGAAACAACAAAAAACAGAAAAGAGAAGAAAAGATATTTGGTATGTAGCT
ATATAATAACGAGCAAAATAAAGTTTATGAAATAATGTGTCACTAGAGAT
GAATATGGTCACCATATGTGATAAAAGATTAAACTTATGATGAACATATA
ATTCTGAACACAAGTATCTATTAAAATACAAAAACAGGAGTTCCTGTCGT
GGCTCAGTGGTTAATGAAATCTGACTAGGAACCATGAGGCTGAAGGTTCG
ATCCCTGGCCTTGCTCAGCGGATTAAGGATCCGGCGTTGCCGTGAGCTGT
GGTGTAGATCGCAGACACAGCTCGGATCTCGAGTTGCTGTGGCTCTGGTG
TAGGCCAGCAGCTACAGCTCCAATTGGACCCCTAGCCTGGGAACCTCCAT
ATGCCGCGGAAGCGGCGCAAGAAATGGCAAAAAGACAAACAAAACAAAAC
AAAAAAAAATAGGTCTCTGCACACCTGGCATATTTACCTCAAAACAGACA
GTTAAAATATATCTTGATAACATCTTTATTTTTATATTATGTTAAAACCC
AGTTAATCCAAGAAATAAAAATAATGATAATGCTTTATATTTATGTGACA
CTTCAGAAATCCAGCCCCATAGCACTGATGGTCAACTCTAAGAAACTCAA
CTCTATGTGTCAGTTCCAAGGAGGCCATTCAGTGGCTCCCAACCTGCAGG
TCTAATTTAAGCCAGCCTACTGCATGTCCTGGATAAGGGTCCCTGGCCCT

GTATGTCTCCCAGGACAGGCCTACCTGAACCATGGTCCAATTAACAAGTT
TCAATATGCACTGCCCCCCAAGACAGTGGCTACCCACCACTACTCTGTAC
CACTCTGTAGTCACTCTTGCTTGTGGGAACTGCAACCTCCACCCAATCAT
GGCAACAAGAGAGTTAGGAAATAACCAAGAGAGGAGACAGGCAGCAGCAG
ATCACCATAAAGTCATCAGCCAAGTGTCATAGCCTTTGCTGGCACTAGCA
CTGGAGCATAAGAAAAACTGAGGGCTAAAAAAAGATGACTGAGGTGCAGT
TGTACTCTTTTCTCATCCATGTTGTATTTCACCTTCATTTCCTGGGACAA
GTTGTTGTTGTTGTTGTTGTTGCTGTTGTTGTTTTTAAACCCCAAATATC
CTCCCACCATGGGCATGGCTTTAGTAATAAAAGAAATAATTAACACTTGT
GCATTGCCAGGCACCGTTTTAAACATTTAACTTATTTGGGAATTACAGTT
ATCATCATTATGTATTTTACAGATGAGGACATTGAGCAGAGAACCAAGAT
TCAAATACAGAAAGCACAACAGAACCTGTGTGCTTAACCACCAGATTATG
TTGCCTCATGCAGCCTGAATAGTTGAAGTCAACCTAATTACACACGCCAT
TCCAATAGATGATTTTCATATAATAAATATAACAGATATATAAAGCCCTC
AGACAATATTTCAAAGGCTAGGGGAAAATGTTAATACCGTTTGCTGTCAG
TTCCATAGAAGATGAAAAGGGATTCAGTATCATTAACACCATCCTTTCCT
AATTTTAGGAAAAGTTTCTCTACTTACTGAGCCCAGTCACCCAATGACTA
TCAGTTTATTGGAAAGGAATTGGAGAAATCTGATGCATTTCTCCCACATT
AAACCAGGACTCAGGTAGGTAAATTATTTAGCTAGTGACAGTTGTATCAA
ACAAAACCAACAGTTTATTTTTGTTTTGTTGCCATTTTTTGGGCTGCTCC
TGTGGCATATGGAGGTTCCTGGGCTGTGGGTCCAATCAGAGCTGCAGCCG
CAGGGCTACACCAGAGCCACAGCAATGCCAGATCCGAGCAGCAACTGCGA
CCTACACACCACAGCTCTTGGCAACGCCAGATCCTTAATCCACTGAGCAA
GGCCAGGGATAGAACTCGCAACCTCATGGTTCCTAATCGGACTCGTTAAC
CACTGAGCCACGACGGGAACTCCAAAACCAACAATTATTAAAAATCAACT
AATTTGGAAACAACTAGAAATATGTGACACAGTATTAGCACTATAATGTG
CAAGACCTGGAAATCAGTTTTATAAGATACATAAACATGCTTATCTACAT
ATATAATTAACAAATAATTGAGAATATTTGAAATATATATATTCTTGTAG
GTGTTTTAAATCTTCAGAATACCATTCCTTGCTATGTGTTTCAACATAAA
TGAATCCCATAAAAATAATATTTTGTGGAAAAATGGTAGTTGTAAAAGTA
TACCAGGTATATGAACTTTAAAAGTATGAAAATCTAAATAATATATTATT
TAATAACTCATGTACCTGTAGTGAAACTTTTAAAAGCTAAAGGAATAATA
AATATAAAATCCAAAATAATACTTCTTATTGGGAAGGAGAGGGATGCATC
AAAGAAAGGAACATAGGGGTTTTCTTCTGTTTCATCCTTAAACAGGGTAT
TGAAATACAGGGATGCTCACTTTATTATTTTCACATTTCACATTTATGAT
GTGCTTATTTGTGTAAATTGCTTTCTTAAAATTTAATCTTTATTAATGCT
TAAGATTTGGGAGGAAAATAATAGCATTTAAGTGGACAATGAGAGAGATG
CAAAAAGAATCCATGCTCATATTTATGGACCCACACCAGGTCCAGATGGA
TTACTTAGATTACTTCTCATTCATAGATGAACACGCAGAGTTCTTAACTG
CAAACAAGTTAATCTATTCTAGCTTATTTAAATACAAATGATGAAGGGCA
CAGATCATTAATAAGACACTGGGAAAACTGGAAAAAGCAAAAATCAGGCA
CTACTTTGAAGGACTCTAGGGCTGCAAAGCGTCTTAAAAGCTGTACTTTT
GCTCCTCCGCTTTACAGATGCAGAGACAGAGAAGTAAAATGACTTGCACA
AAAGCAAACGGTTCTTTAGTGTTAAGAAACAAGCAAGCAAACAAAAAATA
GGTCTTCTGATTGTCAGACCAGTAGCTCATTTCTCTGTATCTGGTTTAGA
CATAGCACCTTTGTTTATTCCCCATCACCCGCTGTAGAGGAGGTTGTTTC
ACGAGTTTGCACACCTTCGTGTAGTGTGGCAGTGGCAAGAGTGTCGGCTT
GAGAATTTAAGCAGGCTCCTGAGCAATATACGCGGGCGGAGGAAAGAAGG
AGCCGCTAATGAATAATCGATGTGGGAGATAACAAGAAGGAAAGGAATCT
GGAGTACAGACAGAGCCTAAATCCTTGAGATGCTCTTCCAGTCGGCAGCG
GGAATTACGGGAATGAGAGAAGATAAACTCAGTGGCAGCTAATTCAACAC
AGCAAATCAAGCAGAAACTTGCTTGCAAGAATTAAAGATCCAACGCAATT
AACGCAGAACCAGGGTCAAAATTAGCGCACCTGCTAGCACTATTACCCAC
CGTACAAGAAAATCATAAGCGAGAGAAATACTCTGGGGAGAAGCTAAAGT
TGTTTAAGATTAGTTTTTGTTTCAGCCTTAAGAGAGAGCAGTGAACATAG
TCAAAATGCAGCCTTAAGAGAGAGCCATGAACACAGCCAAAATGCAGCCT
TAAGAGAGAGAGATGAACATAGCCAAAATGGAGCCTTCACAGAAAGCCTT
CATGGCCAAAATGGTGTATAACCTCCCCGTCGGGGAATCGAACCCCGGTC
TCCCGCGTGACAGGCGGGGATACTCACCACTATACTAACGAGGAAAGCAA
CAATGAGTAAGTCAAAATCAGAGCTTAGGAAAGCACTTCTAAGCCGCCAT
ACTTTTTTTTTTTTTTTTTTTTTGAAAGCTTGTTCGTTGTTGGCCAACTT

GTATATTTACTGCATCTAAACTGACACATTGCAATCGAAACATAGTAGAA
AACCTATGCGTAATTTAAATTTTCCAACAGCACATTAAAAAGTAACAATA
AACAGTGAATCATTTTAATAATATATCTGATTTAACTCAATATTCAAAAA
CGTTATTTCAACATGTAACAACACACAAATATTAATTTTTTTTGTATTTA
ATCTTCAATCCAGTATTTTACACTTATAGACAACACACTTCATTGGAAAA
AATTAGTATTGAGAGTTCATGAAATTAACAATTGAAAAAGTAGGTTCATA
TAGTATGTGTTTAAATTTTTCGTTGATTTATTTTCAATTCGTTAATTAAC
TTCAATTTATTAATGTATTAATTTCAATTAAATAGGCTTAAAAACGCAGT
TCCTCAGACGGTATTAAGTTCCCAATAGCTTATAGCTACCGTATGAATCT
TGCAGGTATAAAACCATAGCCCACTTTCGAGGCTGACAAAACAGAATCCA
GAATATCCCAAAGATTTCCACGAGTTGAAAGTTTAAAAAGTAAGGCAGGA
GGAAAGGTAAGAGAGGAAAGAATACCTTTAGAACCTAACCCCTCCGAAAT
GTGGCTGAGCAGAGAAAGAAGCCCGGGGGGGTATGAAGGAGGCGGTGGAA
GTAGGTGAGGTGCTGCTGCAGTTTCTAGTTTCAGTTTCTATTTTCACTCA
AGCTTTAAAGTTGTTTTGCACACGATATTAATTTCTCGCTGTCTGCAGAA
CTTGCTTCAGCGGAGGGCGACAAAAACGTAGGCTCTGCGTTGGCCGGGAA
TCGAACCCGGGTCAACTGCTTGGAAGGCAGCTATGCTCACCACTATACCA
CCAACGCTGTCGCTTAACAAGGTTTGAATTCTCTGTATAAAATTATCAGA
GGCCACGCATGTACCGATATTTTTTAGTACCATTCTGCATCTAACAGCCC
AAGCTATACGGAGCATTTTCTCGATGAGAATAAAACCCTTCCTGACTACT
CCTCTTAATCGTCTTAGCCAACGAACGACACGAGGCCAGGGACGCAGAGA
CCACCGGGAGCCAGAGCTGGAACAGCGCGGAGCCCGGCGGGAAATCCAAC
GCCTAACTCGACCCGGGCATTTATTTACCCTTCTGAAGACTTCTGCTTTC
CGCGGTCCCAGCAAAGCTGCTGGACGCGCTCAGCACTGGATCCACCGGAT
ACTGGATTCGGTAATGGTGATTTGGGGCTAAATATCCGAGGGAGATCGGA
CGGCAAGGGGCTCCGCCCCGGTGTGGCGCCCCCGAGAGAAAGAGCGAGAG
GAGTGCTCGAAGGGGCGTTGAAGAAAAGGATCTTTACAGTTTAGGGTAGT
GAAGAGGTAAGAAGTAGGACTTGGAGACAGTTTTACTTTTCTTGTTTTGC
GAAGAAGTCCAAGGAAAGTTCTAGTTTTATTTAAAACGATGAATAAACAG
TACAGCCAAAACGATGTCGCACTACAGCACCGTATGATAATCTTAAAGGT
AAAAAAGAAAAAGTTTTTTGCGTCGTGTCAGGATGGCCGAGCGGTCTAAG
GCGCTGCGTTCAGGTCGCAGTCTCCCCTGGAGGCGTGGGTTCGAATCCCA
CTTCTGACAAGCTCTTTTTTTCCCCCAACCATGAGTAGGATTGCATTTGT
GGAGAAAAGAATATTAATCAACACCTTTCTGTGTGGTATATCCACATATG
GAGATTTTGCTTCGCTTTGCATATGAAACAATATATTTAGATTTATCCCT
AACAACAAAATTTTCTTCTTCCTTTAGACTCCAGTTTCTCTGTTTCTATC
TATTCCGTTATTTGAAAATATATTGAGCCCATTAGGGTAGGTAGATACAA
AAGACTCCTCTGGACAAGGTGAAACCCTCCACCCAGATCCCTTAATTTGT
TCACGTTCTAGAGTCACATTTTGGCGAGACTCGGATTCCCCACCATCTTG
CCGGACCCGGTCCCACCACCAATTCTAATTCAATAGGTTTGCTGTGTCAC
CAAAAACCAGAAAAAGAAATCAATACGGCATATGAATGGAAACTAGTAGA
AAGGAAAGATAAATTCATGGGGTTGTTTCAAGAACTTTTTCCTAAAAACC
GCAAAGAAACCGCTTTCTGATTTGGGAGCAGCTGGGTATAGAAAAACCTT
GTCCTTTCCTGACGATCTGAATTGTGAGCGGTCCATAGGGATGAGGATGG
AAGAATCTGGTTAATGAAAGCTTGCCAAACTTCATTCTTTGTTATTTACT
AGACTATGACCTTGGGAAATGTATTTGACTTTTCTTAGTTTGCTTGTAAT
AAAAATGGAGATAGAATTGTTGTGAAGATTAAACTAGACAGCCTAGGAGC
TTGTTAAAAATACAGATCTCAGGCTACACCTCAAACCTACTGAATCCACA
TTTTATAAGATTCCCAGATAATTCATACACACATTAAAGTTTGAGTTGCA
CTGGCCTATCTAATTTAGATGTCTTCCCATAGGTCTGTCTTTAACTTGGA
ATCTCCTTTCTTTTGCCACATTTTGTTCTATATCATTTGAGCACTAACAA
TACACCAGGCACTGTTGGAAGGGAGGATAAAAAGTTAAGGTAAATACTGA
GTCTAAACTAAAAAATAATGATAGTGCATCCATAAGAAAGAAATGTAAAA
AAAAAAAAATAACAACGCTGAACCTTAATCATATGATCAAGGTTAACATT
ATGAGCAATTAGTCACATTGATATCACATACCTCCTGATATAATGCAATA
AGATGGGCACTTCACTCTGTGGTATTCTTTCCCAAACAACACTTCAATCA
CTAAAAAAAAAAGCCACAATGAAGGACAATCTAGCAAATACCTGACGAGT
ATTCCTCAAAATGTCAAGGTCTTAAAAAAAAATCATAAGATTAAGAAGCT
GTCACAGATTGTAGGGAACACATGATAATTAAATGCAACATTTTATCCTG
GATTGGCTTCTGAAACAGCAAAATAACATTAGTGAGAAGATATGAAATCT
GAACAAAGTTTACAATTTTGTTAATTGTATTGTACCAATATCAATTTCCC

AGGTTTAATAAATGTACCGTGTTTATGTAAGACGTTAACATTAGGGGAAG
CTGGATGTAGGATATATGGTGACTTTCTATAGTATCTCTACAATTCTTCT
GTCAGTCTAAAATTATTGCAAAATAAATAGGCATTTTTAAAAAACTTGTA
AGTACTTGCCTATTAGGAGTAGGAACTGGGAGTTGGACAAAAAACTGCTG
TTTTTTCACTAGAAGTGCTGAAGCAAAATTTGATTTTTGAATAACGGGCA
TGTATTTTGATTTAATTTGCTCTAATATTTTGAGAAATAAATATTTAATT
TGTTTTATTTATTTTTGGCAGCACTCCCAGCATGCAGAATTTCCAGGGCC
AGGAATCGAACTTGCGGCACAGCAGTGAAAATAAAGGTTAACTGCCAGGT
CACCAGGGACCTCCTGTTCTAATGTTTTTATTATAAACACACAAAGCTGT
GACTACAAAAATCAAACTAAGACTATATACAATATAATGATATTTAGATG
CCTATTAACAAGAAATAGTCTCCAAAGAATCTAAACCATCAACAGCAGTC
ATTTAAAAGTAGATGAAGTGGATTGTTTGTCTAAATTATCTGTAGAAAAA
CAGATACAACTAAACACAATGAAATTCTCCCTCTTATAATCTGTGTGTAT
TTTGAGGTAGGGAAGGAGAGAGGGAGGCTGTTGAAATGCTAGGAAACTAT
CTAAACCCTATAAGTACCATCATTCCAACTGAGAAGTTACAAATAAGTTG
GGATGGTCAATGTTGGAAGTAAGAAGGAAATTCAGGAAAGGAAAAGTGTA
ATATAGAATGTGTATATGACATCACCTCTTTTTCCAGGTTCCTTCAATGT
GAAGTCAGATTGTACAGGTTTTAGGCCAGGTGCGTGAAAAAGCAGTTAAA
AATGTGAAATGACAACCAGGGTACAAATAGGTAGTTACTACAACCTCCTC
TTCACCTGTAAGAGATCACCCTTAGCAAAATATAATGTAGAATAATGTAT
TTATTAAATTTTGTATATTTTAATACATTATTATATTTATAATGTCAATA
TAATATAGCACTCTAGCCAAGACCGCTTTCAATTCTGAGATAAAAATAAA
TCTCCCTTCCCACACAGGAGAGATGTAGAATGGGTTGTGTTGACTGGGAA
CTAACTTACACAAATTTATCCTTGCTCTTCATTTAATTGGCAGTAAAGTT
GAGCCCAAAGATACTTCCCAGTTTCAACCACTTGCTACAAAAAATACATT
TTCACCTAATGAATAATGTAAATATACAATAAAATATATTTTTCCACTCA
GGGCTCATTATCCAACTGTGTTCTAAAGGAATGATATGTCCAATTTCACA
ATATCTGGGGGGAGGTCTGGTAATAAGTGGCGTTATGGACTGTTTGTAAG
GTGGCAATGCACAGGCTCTAGAATTAGGCCCGATAGGGTTCTTAATCCCA
AATCTCCCCACCACCACCCTTTCCCAATTTAATACTTATGGATCTTGAGT
AAATTGTTTAACCTTTTTTAGCCTCAGAATCCTCACCTTCAAAAAGAGAT
TATACACTGTGATGTTGCTGTGAGGATTAAATTAGATGGTATATGTCAAG
TCTTGGTACACAGCTGACAATTAATAATAATTAGTTATAGCTAATATTTG
TTGCTCAAATACTATATGCCTATCATTCTTCTTCACTTCTCACATATTTA
GTTAGTTGTACCCTCTTAATAATTCCGAGATACTATTCCATCATCACCCC
CATTATACAAAAGGGGAAACTGAAACTCAGAGTTTAACTAGTATGATAGA
TAAAGCTGCAGTGCTGAAAACACAAGCCATCTGGTTCCAATATAGCCATT
TTCCTACACAGTTGCCAGCAGCAGCAGCAGCAGCAGCAGCATCCTCATCC
TAAGGCTCATCAGCAGAAACCAGATGGGACCATGCCATTTCTCTCAAATA
GATAAGATCAATAATAACTTTAATTATACCACCAGACCTAATCTCTTAGT
CTTACACATACTTGGGATCGATTATCTAAAAGAACAACGAGTAAAGTCTC
ATTATTAGAGACAGATAATTAGCATATAAACTCTTTGTCAGTCTATTAGG
ATGCATAGACTGACACACTAAACTAGGCTCCCAATACAGAATTTTTATTT
TAGGGTTTTGCTAGGACTTAAGTTTCTTACCATTCTCTGTTTTCCAACTC
CACCTCCCTCCCTCTTTCAACATAATGTCACAATTCTCTCTTATCTGATC
TCACAATTCAATACTGTCTCAATGTGCCAGCCTCAAACTCTTCCTCTTAC
CTTGTTTTCCCCTATAGAAATCACACACATAGTCTTGTGAATTTTCTTCC
CTTCCTTCCTTCAGCACCCACCCCAACATTTTCTGCTTCTGAAAGCCTTG
CCCCTTCATCTACTTAATTGGATAAAATGAAAACTAAGAAGTTGTTTTAA
AAGTTTATTTAGCTCTATTATTTCACATAACAAAATCCATACATGCAAGT
AACAAAATACATTAAATGTTAGATGAAAGCAACGTTAAAGTGCCACACAA
ATGTTCATAAATGGTTACTTCTGCTTGTTTGAAGTGGGAGAAGATTTTTT
TTTTAAGTCAAATGACAGATACCTCAGGTATAAAAGATACTGACAAAAAA
ACCCCTATTTAGGGGTAAGATTCCCCTTACACTCCCGCACCTGAAGTTAC
AGGCAGGATCATATAGACAGTCTAGAAAGTCCACAAGCTCAGACCAAGTG
GGAGAAGATTTTTTTTTAAGTCAAATGACAGATACCTCAGGTATAAGAT
ACTGACAAAAAAAAACCCTCTCTATTTAGGGGTAAGATTCCCCTTACACT
CCCACACCTGAAGTTACAGAAAGTCCACAAGCTCAGACCTTACAGTCACA
GCTGACTTGTCAGGAGGGGCCATTTAATCAACCTCCTCAATGACAGGGCC
AGTACTGGGGGCTCCCTGTCGAGCTTGAGCACCACAACTGCTACCCCCAG
GGATACCAGAGCCCCCATAGAGCCTGGAGAAGATGGGACGACAAATCTGC

TCCAGTTCCCTCTTCTGATGCTCATACTCCTCTTTCTCCGCCAACTGGTT
GTGCTCCAGCCAAGTAAGGACTTCCTGACACTTGTCTTGTACTTTGCACC
TGTCCTCTTCAGGAATCTTGTCTCTAAGGCTTTCCTCGTGCAAGGAGCCC
TTCACATGGAAAACATAGGCCTCCAGGGAGTTTTTGGCAGCCACCCGGTC
CCTCTGGGCCTCATCCTCAACCTTGTACTGCTCCGCCTCACGAACCATCC
TCTCCACCTCTTCCTTGCTCAGCCGGCCCTTGTCATTGGTGATAGTGATC
TTGTTAGCTCTGCCTGTGCTCCTGTCAGTGGCTGTCACACTCAGGATGCC
ATTGGCATCAATGTCGAAGGTCACCTCAATCTGGGGCACTCCACGTGGGG
CAGGAGGTATGCCACTGAGCTCAAAGCGCCCCAGAAGGTTGTTGTCCCTG
GTCATGGCCCTCTCACCCTCATACACCTGGATCAGGACTCCAGGTTGGTT
GTCTGAATAAGTAGTGAAAGTCTGGGTCTGCTTTGTAGGGATGGTGGCAT
TCCTCTGGATTAGCGTGGTCATCACCCCCCCAGCTGTCTCCAGCCCCAGG
GACAGGGGAGCCACATCCAGCAGCAGGAGATCCTGCACCTTCTCACACTT
GTCCCCCATCAACACTGCTGCCTGCACAGCAGCACCATAGGCCACAGCCT
CATCTGGGTTGATGCTCTTGTTCAGTTCCCGGCCATTGAAGAAGTCCTGC
AGAAGCTTCTGTATCTTGGGGATGCGGGTAGAGCCACCCACTAGGACAAT
ATCATGGATCTGAGCCTTGTCCAGCTTGGCATCCCGCAGGGCTTTCTCCA
CTGGCTCCAGGGTGCTGCGGAAGAGGTCTGAGCACAGTTCTTCAAAGCGG
GCTCGAGTGATAGAAGTGTAGAAGTCTACACCCTCAAAGAGGGAGTCGAT
TTCCAGCGTGGCCTGGGTGCTGGAAGACAGGGTGCGTTTGGCGCGCTCAC
AGGCGGTGCGGAGCCTGCGCAGGGCCCGTTTGTTCCTGCTTAAGTCCTTC
CTGTGCTTCCGTCGAAACTCCTCCATGAAGTGGTTCACCAAACGGTTGTC
GAAGTCCTCTCCACCCAGGTGAGTGTCTCCAGCCGTGGCCTTCACCTCAA
AGACACCTGCGTCAATAGTAAGGACCGACACGTCGAAGGTTCCTCCACCC
AAGTCGAAAATGAGCACGTTCCGCTCACCAGCGCCCCTCCGGTCCAGGCC
ATAGGCGATGGCAGCAGCTGTGGGCTCATTAATGATCCTCAGCACGTTGA
GCCCTGCAATGGCACCTGCGTCCTTGGTGGCCTGACGCTGAGAGTCGTTG
AAGTAGGCAGGCACGGTGATCACAGCGTGCCTCACGGGCTGGCCCAGGTA
CGCCTCCGCAGTCTCCTTCATCTTGCTGAGCACCATGGACGAAATCTCCT
CCGGGTAGAACGCCTTGTCCTCCCCACGGTAGGAAACGCGCACCTTGGGC
TTCCCACCCTCGCTCACCACCTGGAAAGGCCAGTGCTTCAAGTCTGACTG
CACGGTGGGGTCTGCAAACTTGCGCCCAATTAGCCGCTTGGCGTCAAACA
CGGTATTCTGCGGATTCAGGGCCGCCTGGCTCTTGGCTGCATCGCCTACC
AGCCGCTCGGTGTCGGTGAAGGCCACGTAGCTGGGGGTGGTGCGGTTGCC
CTGGTCATTTGCTAGGATCTCCACCCGACCGTGCTGGAACACACCCACGC
AGGAGTATGTGGTACCCAAGTCGATGCCTATGGCCACTTCTCTTGCAGCG
GACATGGCTGTGGCTTCTGTCCACGAATGCAATATGAAGGCAGATGCACC
AGCTTTCGGAATTTAGTGCTTCTCAACGTTGGAGGTACGCTCAGCACTGC
CACTCTTGCGTCCACCCAGCTCGGATCTGCTCTCCCTCCCCTTCCAGGAG
CTTTTTATCCAGTTGCGGAAGACCCGCCTCCTCCCGCCCCCGCAACCGTG
AACCTTCCAGCGCCTTCCGGGCGAGCCTAGAGCCGCCCAGCCCGCCCGCT
CTGGCTGACTCGGGAAGAAGGTTCGGGCGGATCGTCTGTTCAAGGAAGGG
TGGCCTCACCTGCGTCTGACTCAGAGCCACAGAGAAAGTTCCAGCTTCTC
CCAGCTGGTTCTAAAGGCCATCGGTGGGGCAGAAGTCAGGTAGGGCAGTA
ACTTCTCAAAAGAACACCCTCCTGGGTTGAAGAAGGCAGGTCTGAGTCCG
GCACCTCCAGAAAAGCCCAAAGAAAATGTCACCATGGGAAGAGAGCCATG
GCCTCAAGGCCAAAGATTTAAATTTCTTAATCCTTAATATTTGCAGGGGA
GGGGGTTCTTGAAACATAAAGGCAGTATTATCAAAATTATGACTGAGAAG
AACAGCTAGGAAGGGACCTAGGGCTAGGGATGATGTTAAGAGATCATGCT
GCAGGCAGCTCTTCTACAAGATGCATTGGTGGTTCAGTGGTAGAATTCTC
GCCTGCCACGCGGGAGGCCCGGGTTCGATTCCCGGCCAATGCACAAGGTT
GTCTTTTCTTTCAGCAGCTTCAGTGATTTTAAAAAAGCTTTGTTTTGTTT
TTCCCCTACTAGGATTTGTGACAATGACATAAATAAAACCAGTTAAAGGC
TGGGTTCTTCTGGTTTTCTCTCCTTTATCAAGTGGCAGCCCAGAAAGGAG
CCAGAACCTGGAGCTGTGGCTTGGGAACAGCCTAGGAGGAAGGAAAATGC
AGGTGAACTGTGGTGAGAATGTCCCTTCTCTTATCCAGCCCCTGCGGGCT
CATATCTCCTGGGAGCATAGTACCAAGGGATGAGTTGGAAAGGCATAGTG
TGTGTTTTCTTTCTGAGGCCTGGACGGGTTCCCAGCTCAAACCCAGAACT
CAGGTGTGCAATAAATGCCTGGAGTACAAGGCATCCTGTGCCCCAAAGGC
CAAGGGCCAAGATGATTTCACTGGTCAATAAAGCCACATTTGATCCCATC
AGATCTATGGCATTGCAAACTGAGTCTTTGCCTTGTCTCACTTTCTTTAA

TCCAGAAGATCTCATTTAAATATTTTGGTGTTTCTCTTTGGCATGGACTT
CTAAGATACCGAAAGGAGAAGACTTTTTGGAGACACGAGGGTGGAAATGG
AGGAAAGAGAAGCCAGTGTTGTCTGTAATCTCCTTCTGGGAAATGGAACT
CTGGTTTCCCACATGACAGACAAGTACGGTCACTACTAGACTAAGAGGAT
AGTACCCAGGAAAACATGATACATTCTAATGTTAAACTCTAGTAGCTTCA
ACTTTTATGTACTATATTATTATTCATCTGATTCAATGAAACTACTAGTT
TTTAAAAAGCAAAACTTAAAAACCTTTACTCTGAGTAAAGTGGCTCCTTT
CTCCTCAGGTGGCTTAGAACTCTTTGGAACACTTCAGGGCCTGAAGAGGG
AGAAGGAATCAGGATGCAGGTATCAATCCCACTTCTGGACTGGGTTTCTG
GGCCAGCCCTTTCCCAGTGTATTGATGAGAAAGAGCTGACAAAGTTTGCT
GCTAGTGAGCTCAGCTATACTAAGAAGGTGATCAGTCCATGAAATAAGGG
AATGTTTGAAGAACTGTGCAATTAATTACTTCCACAGTGGAAAAGCAAAT
CATCCTCAAAAAGGAGCCTATTTTATAACTCCAGGAGGGAGAAAAGCTGT
CAGCTTTAAATTCATTCACTCTCAGAGTTTAGGCTGGCTAGGCCTACATG
TCAAAGTCAGGACATTTTAGGCAAAGGCCTGCAAGAAAAAGATCCTTATT
TCAGAAAGGAAAACATGGCCTGCCCTGAGGTCTGTTCAGGAACTGCCTTG
TCCAAACTCCTCTAACCTTCTCTCCCAGCCCTCAGCAGCCGCAAAACCCC
ACCTGCACACCTACCCCCAAGAGTTTCAGGTATAGCCCTAAAGCAAGCAC
CTCCATACCTGGACAGAGCCTCACACACACATTTCCAGTGTCACTCACTT
TATTTTTTTAACTCAAAAGAGTTCAAATATAGGGAAGCTTATTATGCAAT
GCAAACTTTATAATACACTTAAGCATATCCACTTATGTGAAACATATAAA
TTTGAGTGTCTTTCAAAGTAGAACAGTGAGAGTTCCCAATGTGGCTCAGT
GGTAAGGAACCCAACTATCTATGAGGATGCGGGTTAGATCCCTGACCTCG
CTCAGTGGGGTTAAGGATCTGGCATTGCCAAGAGCTGCGGTGTAGGTCGC
GGGCAAGGCTGGGATCCCATGTTGCTATGGCTGTGGTGGCTGGCAGCTAC
AGCTCCGATTCGACCCCTAGCCTGGGAATTTCCATATGCAGTGGGTGTGG
CCCTAAAAAGACAAATTAATAAAGAAGTGTAGAACAATGATGACTTAAAT
AGAGATTGTGGAGAGATGCTCTTGACCTAAAACTGAGTACAAAAAGCAAG
TTGAACAAAATGTATAGTATGATCCAAAACAATATGTAAAGGTCTATGAT
ACCAATTACGTGTACATCTTTGAACATCCTTAAAACCATCTGGGCCACAG
CTTTCTCAATTTTATTTTATTTTATTTATTTTCAACCATGCCCACAGCAT
GAGGAAATTCCTGGGCCAGGGGTGAAACCTAAAACACAGAAGTGACCCAA
GCCACAGCAGTGGACACCACATCCTTAACCTGCTGAGCCACTGGGGAACT
CCTGAGCTTTCTCAGTTTTAAAATGAGGAGATTGAATTTCACCATTTTTA
AGTCTTTGTAGCGTCAAAAGTTTCTGATTTCTCATGAGCAAAAGAGGATT
CCTGTGTCTCCTAAACTATGTACCTATGCTATACCTGAATGAACCCTGCC
CCTTTTTTTTTTTTTTTATTGGCAACACCAACAGCATACGAGAGTTCTGGG
GCCAGGGATTGAATCTGGGCCACAGCTGCAGACCTGCACCAGGTCACATC
CTTTAACCCACTATGCTGGGCAGGGGATTGAACCCATGCCTCTGAAGCAA
CCCAAACTGCTGCAGGCAGATCTTTATTTTTTTTTTTTTTTCCCCTCTGT
CGTTTTGGTTTTTTTAGGGCTGCACCTGCGGCATATGGAGGTTCCCAGGC
TAGGGGTCTAATCAGAGCTGCAGCCTCCAGCCTGCACCACAGCCACAGCA
ATGCCAGATCCGAGCTGTGTCTGCAGTCTACACCACAGCTCACGACAACC
CCTGATCCTTAACCCACAGAGGGAGGCAAGGGATTGAGCCCGCAACCTCA
TGGTTCCTGGTCGGATTCATTTCTGCTGCACCACTACGGGAAATCCTGCA
GTCTGATTCTTAACCCACTGCACCATAGCAGGAACTCCTAAATTCTGCTT
CTGTTAAGCATAAGAGAAGTGATATGCTGTGTTTGTCGGAAAGTGCCATA
TTTATTTTATTCATTTTTTGCTCCTCACACTGGTACAATAAGGAATGTTA
GTGTGATAGACATTGATGATGCTAACCAATATGTATGATTCTCCTCCTTC
TAGCCTCAGAAAGGGTTTGCAATTCCCTGCCTCTTGAGATCAGGTCATAT
GACTTGTTTGAGCAATGAAATTTAAATATGTTACTTCTGGACAGAGGGCA
TTTAATTACTATCTTAATTGCTACTGTTGTCTTTGACTTTCTTGGCAATG
TATGGTAGCTAGTACTGAAATGGACATATAAACCTGAGCCAGCCCATGAA
AGACAGCTTCCTTGGGGACTCTTCAGATATACAGCAGAAAATGAGAAATA
AACTTCTGATGTTTTATGCCACCAAGATTTTTTTTTTTTTTTTTTTTTTT
TTTTTTTTTGCTTTTTAGGGCAGCACCTGCAGCACATGGAGGTTCCCAGG
CTAGGGGTCAAATCAGAGCTACAGCTGCCGGCCACAGCCACAGCCACAGC
AATGCTGGATCCAAGCTGCATCTGAGACCTACACCACAGCTCATGGCAAA
CCCACAACCTCATGGTTCCGAGTCAGATTCGTTTCTGCTGCACTACACCA
GGAACTCCAAGATTGTATTTTTATGATCTGTGAGTTTAATATGCAGGTTA
GAGCTGGTCAAGTAGGATTATCACCCATTTTTTTCCCAATGTGACTAGCC

ACAAAAATGTAATTCACTCAGTTCTACCTTCTAGTCCATCCCTACTCCAA
CTTACACCTCCCTACCTTTTTTTCAGTTACTATAGTAAAAACAGTCTTAT
CTGTTTAAGGGGAAAGTGCCAGCCAAATAGTGTGCTCCAAAGACTACAGT
TTCCATAAACATTTATTAAATACCTATTATGTTCCAATGACATTATGTTT
CCATGTATGTTGTTAAACTTTAACCTCAACAAGATATCCCTGGGGAGAGA
GAAAGATATTACCATTTCACAGAAGAAGAATATAATTCACAGAGTAGTTA
AATTCTTCAAGACCAATCAGCTAGCATATGGTAGAAATGAAACACAAATT
CAGGTTGTTTGGCTCTATTTTCAGTATACTTTTCACAATGCTACTCTATA
GGCTTAGAAAGTTAATTAAATTAAAGGGAAGCAAGTCCAACTGATATACT
ACTTTAAAAGATGTGATGGGTGAAAGTAAATTATATTAAAGAGTTTAGGC
AAATACATTCAAAGCTGAAATGCATGTACCAGTGGTATATCTTGGATGGT
ATCTAACAGGGTGTTCAGTAATATTGAATTGGATTTCTGCTTCTGGGAAG
ATGGAGTAGATGTGCCTTTTTAAAAGTATTTGATTTCAAATTATAAGGA
ATTTACTCACACCGCAAATAGGTATTGTATGCTTTGTCATGTACCAGATG
CTGACCTTGACTCTAACTGTATGGTAGTGAACAAAACGGAAAAAAAGCAC
TGGATCCTGGAGCTCATATTCTAATGGGAGAGGGGATAGTCAGTTGGTAA
TAAGTACAATGGGTAAAAATACATCAATAAACAATTAAACAATTGGGCAT
ATGGTACTTCAGCTGGTGATGAGTACAATGGGGGAAAAAATCAAGCAAGA
GAGGGAGATTAGGAGATAGTGGAGGAGTGGTGTTGTATTGAATGGTCAAG
GAGACCTCACTGATAAGGTGATATTTGAGTAAAGACCATGCTGAAGCACA
GTGTGGATATCTGTAAGAAAGAACATTTCAGGAGTTCCCGTCGTGGCGCA
GTGGTTAACGAATCTGACTAGGAACCATGAGGTTGCGGGTTCGGTCCCTG
CCCTTGCTCAGTGGGTTAACGATCCGGCGTTGCCGTGAGCTGTGGTGTAG
GTTGCAGACGCGGCTCGGATCCAGCATTGCTGTGGCTCTGGCGTAGGCCG
GTGGCTACAGCTCTGATTAGACCTCTAGCCTGGGAACCTCCATATGCCGC
GGGGGCGGCCCAAGAGATAGCAACAACATCAACAACAAAAGACAAAAAAA
AAAAAAAAAGAACATTTCAGAAACAGTAAATAATATAAACCTGGAGCAGA
AGAATAATTGAAGTATTCAAGGAAGCAAAGAAGAGCAGTAGATGAGGTCA
GAGAGGCAGTGAGGAAGAAGATCATGTAGTGCCTTGTAGCAAAGGCTTTA
GCTCTTACTCTAAGTACAAGAGGGAAGCTATTGTATAGCTTTGAGCAAAG
AAGTGACAGGTGCAATGCACATTTTTAAAAGATCACTTTAGCTGTTTTGT
TGAGACTAGACTGTCAGGTGGCATGAGTGAATGCAGGGAGACCAGTCAGG
AGGCTGTGGAAGTGAGAGATGATGGTAGCTTGGACCAAGGTGCCAGTAGT
AGAGTACTGAGAAGTTGTCAGATTGTTGGATTTATTTTGGAAGTAGAGCA
GGGTTTACTGAGGTTTCATATATTTGAGGAGCCTCTGTCAAACATCTATG
AAAACACATTTGCTGTGAGTCTTTGAAGAGCTGGGTGGGTTGTGACTGTA
CCTTGGCCTTCAGGAAATCAGTAAAGGTAAGACCTGTTAGGATTTAGCTC
TGAAGATCACAGGCCCTTTCCTTCCTTGGAGGGTCCTTGGCCCAAGTTGC
TGTTAAGTCGGGGCTGGGGGAAGAGTTACAGACTGAAGAGATGCTGCTAC
CACAGCTGTTACACCATGGAATGAGCGATCATTTGTCCTGAGGGCCTTGA
CTCCATCTGACTTTGCTGTTCTTCCACTCTCCCTTTGAGCATTGAAGGTC
TCTCTGGACAGAAAAATACAGCCCTGTATCCACTGCAAAAAGGAGCCCCA
TCGCCAGGTAGAAAGTTATTTGATACCAAGGTGGAAAGATGAATAGAATT
GCCGGAC**CT**AATAGGAGAGGCAGAAGGATGGGAAAATGACACTTGTTAAG
GCAGAAATTTAGAATAAACAATCAGGTCCCTAGTGAGAAGCTTTTGAGAA
GGCTGAGGCCAAGGCCAAGACAGACAGGTAGCCCAGAAAGGATTCAGAGG
ATCTTTTCCTCTCCCTGCCTTCCCAAATAATGCACTGGGTCAGCATTTCC
CAAAGGATAACCTCAGGAGCACATCTGTTCTTTAGGACATTAACAGAAGT
TATAGAGAAAAAAGATTTTATAGCTACCAGGTTTAGCAAATGCCTTGTGA
CACTTCATTTAAGAGTTTTCTTTAAGACAGGGCTTCTTGAGGACTTTAAT
GTACATTATGAATATAAAAGGAAAGGAACAGAGTGCATAGCATGTTCTGC
ATTGATTTGACCACAGAACCCCTCATTCTGAAACATCTCTCTGGGAGTTA
TGTGATTGGGGAACAGCCTTTGGAAAACGTTGAACTAGAGAATCCATAGA
CCACATCAACAGATTTTCACTCACCACCTCCTTTTAGCCTGAGACCAGGT
ATTCACTAACCCACTCTAGTCTGGCATAAATGCTTGTGATGCCATCTGAA
AGGGCATGATTTGGAATTCACAAGGCACAACTTCTCAAGAAAAGGCAGAG
GCCTAGTTATTTGTATGTGCAGAGGGAGTCTCATGCCAACATGGCTCCTT
TGGCTAAGATTTGAGCCCAGCCTGAGCAGTTCACTAGGGAGACACAAGCA
AGCATTAGACTCCTAGAGGGACATATGATAAAGTACATGGAATGAGGTGG
GAAAGAGGGAGAATGGTGGATTTCCCTACTGGCTCAGTGGATTAAGGATC
CAACATTGTCACTGCTGTGGCTCTAATAACTGCTGTGACATGAGTTTGAT

CCCTGGCCAAGGAACTAAGCCCCAGCCCTCCCCCTGAAAAAGAGGGAGAA
TAACAATATCTGGAAGATGGATATGGAGGATAAAAAGGTGGTTTCTGTCC
CTTAAGAGTTAAAACTCCAAGTGAATGACACCCATCCCTATAATCCCTGA
GAAATTATCCAGGACAGAGCTGTGTATTCACACAGCAGTTGGGTCAAACT
AAATATCCTGAATTCCAAAAAAGATTAATAATCCCCTCACTACCCATCTT
TCCAAGGTGTAAAGCTCCATGAAAAGACTTAGTTCCC**AC**CTTGGACAGTG
ATGTTCACAGGCTGTGATGAGTATGACGTCTTCCCAATTGATCCTGTGCA
GTGGTACTCACCACTATGACTGTGGTTTGCATGTGGGATGGAGAGGCTGG
ACTCCACATAGGAAAATTTCTTAGATTTTCCATTCTGGAAAAATACGACC
TTATGCAGAGGCTTGTTTCTCCAGCTATGGCACCTCAGCACAATGGCTC
CCCTTCCTGGAACACCAGGCTAGGGGTCTGGAGCAGCAGCCAGT**CT**GAAA
GACACAAAGGGACCACAGGACCCAGGAGGCCTGACTCAAATCTTAGACCA
AATCCTTACTGGTGGTACATTAGATGTGGAGAGGAGCCTACGTTTGGAAT
CAGACATTCCTGATCTGAATCTTGGCAAGTAATTTAATTCTTCTGGACCT
CGGTTTCCTCATGTGAAAATGGTCACAGTCATACCATTACCAATCTAGAG
TAGTTCTACCACCTACTATGGCATATAGTAGGTATTCAGGACATGCTTGT
CTTCTAATTCCTGTCCTGGAATAAGCCAGCTGCAGTTCTGTCCTGTCCAA
CAAACTTCCACAGGAAGGGCATAAGAGACCCTGGTACCATCCCTTCCCTA
AAGGAGGAATCAACTCTGACACAGGAGACAACAGGTGAGGGGGACACAGC
AGAGACATACATCTTTGGGGAAATGTGTGAACATCCAACAAATATTAGCT
TATTTGGTGCATATTAGTAAACGTGGAATTTCATTTTAGCAAGGACTTTA
TCCAAAGGCAGCAGTGATATGGGTGGACTGCTAGCCTGCTGTGTATATTT
CCCTCCCATTTTACATCCTTTTACAGAGGAAGAAAAAATATGTAATTTTT
TGTTGTAAAAGGAATAGAAGATGAGGTGGGTACTTGTAATCCTGACCTCA
GCCCACACACTCCTTTTTCCTAAAGCTGTAGGATTGAGCAGAGTTGGAAT
TGTAAAGAAAGGGTAGAAAACAACAGATACATTGGAAGGGAGTCCCTCCA
GATGAGGAAAAATAGAAACAATAACCACATGTATGCCATCAAACACACTT
AGTCCCTGCACGAGATCAGAATGATGCCAACAGAAGTCTCCACACAGATG
AGGGAATAAGTGACAACAAAGAGAAAGCTCTGGGCCACAGCTACAGTGGG
ACAATCCAGAGGAAAGCTGGGGGACACCAGATCTTGCTACTTGTGTCACA
GGAGCTAGAAAACCCCACTTCTCCCACTGGGAAGGAACCCCTCTGTCCCA
GCCATTTCCTTCCCTATCATCCTGTGTATTCTTTCTCTTGGAAAACTCAG
ACCCTTGCAGGACATGCCACACCAGGATTAGGTGAGCAGTCCTGGTTCTC
TAGAATGCCCCTCTTATAAAGCTTGAGTTAGGGTAGAGGTAGGGAAGGCT
AAGAATTGTAAAAAAAAAAAAAAAATTTAATGAAGTGGAACCCAGAGAAAA
TGTCAAAGCTGCTTGCATGTAGCACCAGCAACCAATTCTTGCACTTCCCC
TCCCTGCTTGGCTAAAATCCCCTCTACAGAAGGTGCACCTCATTAGAACA
GCATGTTCTCCCTAGCAGTCTCAACTGGTATGAGCTTATAACCTGGACAG
TGGGGAGTGTCTGCCTAAGGCTGGTTTGTTCAAGAGGGCCAACTTACGCC
TCCTTGAGGACAATTTCTTGGTGCATGTCTTGAGGGCAGGGCCCAGTTTC
ATGTCCATGGTACTCATGAGATGTTCTTTAAGAAATAAAAGGGCTTTTGC
ATCAATCTCTTGGGAAGAGAACTCCTCTGCAATCTTCTGGAAGTCTTGAA
GAGAGGCGATAAACTCATACAATCTTCTACACTCCACTGGCTAAGTTTAC
TGGACAAGAACACAGGGTTGATGCTGTGAATCCTGGGGTAGGCAGAACTG
TGTCAAAGTTCCCTGGGTCACACTCCCCCTGACCAGCTCTCACTTATAAA
GACCCAGGGACATTGGAGAGAGTGCTTCATCCTAAGTGGAATTCTCTGAA
CCCTAGCTAGAGTCCTCTTACCTTCGGAGCCACTTGCCCTGTATCTTAGC
ATGGGTGATGTCAGAGGAGCTGCAGTAGGGTCCACACTGGTGAACACAAG
CATTATGGACTTCTCAAGACACTTCCATGTGTTTTCCTCTTCAGGCAGAA
CTGAGGCTACAGCTCACACTATACTTTTTTTTTTTTTTTTTTTTTCATT
TTTTTCCACCCCATGGCATATGGAGTTCCCGGGCCAGGGATCAGATCCGA
GTCTCATTTGTGACCCAAGCCGCAGCTCTGGCAATGCTGGATCCTAAACC
CCCTGTGCTGGGCCGGGGATCAACCTGCTTCCCAGGGCTCCAAAGACAGG
GCCAGTCAAGCTCCTATACTCTACCTCTTAGTGCAACTCATGGAGTAGAA
ACTCTTGGAGCCACAGAACTGTTCTGCAGGAGTGCACGTGCCACAAGGCT
GGAACTTCAGGAGATTTGTCCTCTTGACTACTCAGCAGATGGGCTGTCCC
CTCCCCAGGGGCCACCTGACTGATTCTCATTCAGCCTTGTCGGGAGGCCA
GTCTACATAGAGCAGCTTCTCAGACTTTTGAGTAACAAACAACCCACTGG
GAAAGGTTCTGCTCCATCCTGGATTCCAAAGCCCTCGATCATGTGGCTGA
GCATGTGGGGCTTCACCATGGAGGTTTGAGTCACCCATTTGTCTGGACAC
CATGGCTAGTGTAGGAGGTGGTGCTGTGGGCAGGGGCCATGGCTGCCAGT

TCCCCCTTTGGTGAATTAGTATTCCCTCTGGTCACTGGTTCAGCTTCTTC
TCCAAACTGCTCTCCTTCTCCTCGGTGGCCTTGGAGCTCACTGCTAGTGC
AGAACCCTTGGCAGGAACCCTGAACCCAAGGTGAAGACATCATCTCCTTC
CTCCTTGCATTTGACAGCCAATGTCTGGGTTTGCACAGCAGGTACACAGA
CTGCATGTAGAAGACAACAGGGACCCAGACCTGTTGGCTGAGGGGCAGTG
AGAGTCGTGGCTGCTGCTGCTGCTACTGCTGCACCCACTGGAGGTGCTGT
GGGGAGAAGTACCGGGATTGACAGTGCTGGAGCTGTCACCAGAGGTCGAT
AGCAATCTGCTGCCTGGTCATCACCTGTTTCTCCAAGAGCTGGATCCGTT
GCTATTGCTAAATTGGTGAATGGGGCTGGATCTGTGTGTGGGTGACTGAG
CTAGTAAGGGTCTGCCTGGGGGCAGGTGTAGCTCTCTATGTCAGGTTCAT
GCCCACATCCTGCTGACCACTCCCATCTGCTTCTGTCTTCTTGGCCACTG
CACTTTCTGCCTCTGTCCAGCCACCCTGGATCACCATCAGTGTTTGAAAT
GCAGGCAAGGGCTCCACCACATCCGCCTTTCCTGGGACAGCTCCCACCAC
CACCCATTCTGGAGGAGGAACTGACTCAATGGCCCATTTCTACCTGGATC
CATGGACCTCAGAATGCTATTCCCACTGCCTCCACCAAATGAACTAATGC
TAAAGATTGGCCTGATCCCTATAGAGGAAACCTGAGCCACAGCCAGAGGC
CTGGGGGAGGCTAGAGAGAGGAGAATTGGGTTGAGGCCTGTTGGTTCCTC
ACTGCCCAGTGCTGCTCCTGATCTGCATCTACACGGACTCCAGGAGCCTG
AGCAGCTGGCACCTCCAGCCAGACTACAGTCACTGTCCTAATAGGCATCA
GGATGAGTTGAGAAGCTAAAGGCACATTCCAGCCCATGGTCTGGTTTACT
TGGAAAAAGTTCCCCTGCTGTAGTCTTAGGTACATCTGGGCCTGGAACAG
GTTGAGGGGAGGAGGAGGTGGTGTTTCCCAGGAGCAGAGCTTGGGTCAAA
GTGGTAACATAGGGAGAGTTCACACTCGTGGATTGGCTGGTCAGTTGGGT
GGCTGATGTTGTGGCCAGATTGACTGAGGCCTGGGTGTCGGTCAGCTGCT
GTGTAGTGCTGGTGTTTGGGGAGCTGGCCTGCTGACTAGCAGCACTTGTG
ATCAGCTGCACAGAGGCCAAGCTATGCAGCTGGGTCCGCTGAGCAGCAGG
TTCACATGAACTGGACTGGTAGAAAGACGGGGGCCCATAGAGCTATCACA
GCAATGCCTGCAGAGCCTGTTCTGCTTGCTGTTCATACAGTGGCTTTGAC
TATCGGGGGCCGAGGGCTACCACCTGAGTTGGGACTCTCATTGGAGCAAC
TGGCATTCTGTTGGCTCTAGTCTCCACGATAATGAGCCAGGGTCCCCAAG
GCTGATGCTCTGGCTCAAGACGTCATAGCAAGCTCTGCACCCTGATCCTC
CAGGGCAGTATCCAACTCATACTCAATGCCAAGGTCCCCTGCTGTCAGGC
AGTGTGGCTGGAAGTGGGAGGTGCTCATACAGACATGAGGGGTGACTCAT
CTCCACCCCATTCCTGGAGGGTCGGGGCACTGAGGTCAGGGCTCTGCCAG
GCCTCTGGGGATTCCCTCCAGGACCATCTTTCCCTGCTAAGCGCAGAGAG
GAAAGATGAAAGGTCTGGGGCCGAGGCTCACTTGGCCTATTAGTTATCTT
TGAGGAAAATTTAGGAGCCAGGTAAGAGAAGGGGACAAAGTGAAGTGTAA
CAATGACAATATTCATCATAATTGCATTACGCCTTTTCTTTATGGAGAAC
CTACTTCTTGCCAAGATCTTTGGAGAATCTTTTTTTTTTTTTTTTGTCTT
TTTGCCATTTCTTGGGCCGCTTTCCTGnGGCATATGGGGATTCCCAGGCT
AGGGGTTGAATTGGAGTTGTAGnCGCCGGCCTACACCACAGCCACAGCAA
TGCGGGATCCGAGCCGTGTCTGCAACCTACACCAACGCCGGATCCTTAAC
CCACTGAGCAAGGnCAGGGATCGAACCTGCAACCTCATGGTTCCTAnTCA
GATTCGTTAACCACTGAGCCACGATGGGAACTCCCAAGGGCTTTGGAGAA
TCATTGATCTGTTCAGCAAACAGTCATTGAGCCCTGCCCTGTGCCTGAGG
CTGGAAATACAAATAAGATTAAAACACCACTTTCACCTCAAGTTGCACAG
AGAGCCAGGGTTGGGGGTGGGGCAGGGAAATGCTGATTCTCACCAAGACC
TGATAGAGGAGGCAGTAGTCTCTCCATTGAGCAGATGAAGAAACTGTGGC
TCAAAGCTAAATGGAATCATCATGTTTACATGGCTAGTAAGTTCCAGGGC
TAGGATTCAGTTGTGGTCTGTCTGTGCATTTGACTCTGCTGTAAAAATGG
AAGCGGTCCTATGAGTTAGGGATTTCCTTCTGCCTCTCTCACACTCAGCT
GCCTGTTTATGGCCTTCTTACCTTCTTGTTCAAGTCAGGACCGAGGCAAA
ATTGCCCAAATACTGAGAAATCTCTTAGGCAGTTCCACCCTACCGCACAT
ACTAAACCAAGGTGGAGGGTGGGAAGGGAAATGAGTGGGCAAAAATGACT
TCAGATTCATAGCCATGCTTCTCAGTAAACAGGCCACCCTCTTCCCTAAA
AACCTCTGCCCTGTAAGCTGATTTCATCTCTCTTTGTCCTCCCAGACTCT
CCCAGGCCTTCTTCCACTA**AC**CAGAAATCACATCCAGATGCACAGGGTCG
CTGAGACTGGAGTAGGCAGTCTGGCACCTGTAGTATCCGCTGCTAGCTCT
CCTGGCCTTAAAGCTGAAGCTGGGCTGGTTCTCGGTCCAGGTGAAGTTCC
CATTATGGAACCACTGGGTGGTGGTGTTCCCAGGGTCATGGTCGCCCTGG
CACGTCAGTGTCACAGAATCCTCCTGGAGCACATTGATCCATGCAGGCTG

AAGCTTCACCTCAGCCTTTGGGAGAACTG**CT**AAGGGGCAGAGAGAAGAGG
CAGCATGAGGAAAGGGGTCCCCTAGGGCCCAAGTAGACCCCAAATGTTGG
ATAATGGGAAACTGTGGCCCTGCTGGAGACATCAGGAGAGGAACCCAACC
AGAACTGTTGTGCAACTGAAGGCAATTCCTCCAACTCATATAGCCCACCC
AGGGGCCCCAAGAAGCCAAGTTCTCCCAGGGAGCTTGTCTGCTCCTCAGG
CAGATTAAGAAGGAAGGGAATAGGAGAGGGGGCAAGGCAGACAAATACAA
AATACAAGTACACCTGACCAAAGGATGTCTAACCGGTTCCTTCTTTCTCT
CCATCTCTCTGTCTCATCTCTCTCTCTCTGCTTTCTCTCTTCTCTCTCAC
ACACATGCACTATACATACATCTTTTTGGGGAATCAATTTCTCTCCTGTA
AATTGAGGTATTTGGACGTTAGTAGATAAACGCTCTCCACAGTTGGATGA
AGACAAGTGGGGACATCTGGTGGAGATGCCAGGCTGAGAGTCAGATGTCA
TGTTTCTCTTCTGACCGATTGTCATTCTGAAACTACACTCCTCCTCGGAT
GGGGCTCAAACCCATCTGTACCTTAGATTGGGACTTGAATCCACTGTCGT
CTAATTAAAACTGTTCACCTGGTTTCATGACTTACTGAAGCTCAAGTTGT
TTTGTCTTGGTGCACAAATAATTTAGCATGAGGCGAAGTTGCAGGCAAAG
CATAAATTTCTTAGCATAGGACCCTTGTGACAGGTACTAACAGGCCAGCA
AGGGAGCTCAGCCCCTGAGAACAAAGAGGGCTCCATTTGGAGTTCTCCTA
TGGCACAGTGGGTTAAGGATCTGGCATTGTCACCGTAGCAGCTTGGGTCA
CTGCTGTGGCATGGGTTTAATCCCTGGCCTGGGAATTTTTGCATGCTGCA
GGCCTGGCAAAAAAAAAAAAAAGTCTACATTTTTATAATCACAAAGTGGAG
ACGGGAGAAAAGCACCTTCTTCCTCATTTTTGCATAGATATAACTACATC
ATTAGTTCTTCCTTTGACCCTATATGGTCTAACCAAGACTGTCTTAGCAC
TATTTATATCATATATATATTAGCAGAAAGGTGGTAACATATACTAAAAT
ATGGTAATTCATCTCAGGTTTCAATATAATGTCCCCTTTCACCTAGTTTC
TTTCTCCTTTCATTTATATTCCCATCTTGGCTCCATGCAGAAATGCTGCT
CTTCAAGAACCATTAACTTTCTAACTTCATGTAACAAGATTCAGACCCCA
TACCTATTGTCTTGTGTTTTAACTATCAGGACTTGTGGCTTGAGTATGCC
TGGCTCTTGAATGCACCTGGTCTCCTTCAAGATAGAGTAGATGGTTGATA
GGTTACTGGATTTTCTTGAGTGGTCATTAGCTTACAACTGTCTCCCAGAC
TCCCTTAAATTTCTCTTTCTGTCTTTATTTCCCCTAGTGGGATTTCTAAA
CTAATTATCCTACTTTATCCCTATCAATTCTTTACCCCTTAGAATAAATC
TATCCTGACACCATAAAAATATTAAAAAATAAATTAAAAACCAAAAAAAA
AAAAAGCAAAAAAAGGGAGTTCCTGTCGTGGCACAGTGGAAACGAATCTG
AATCCAACTAGGAACCCTGAGGTTGTAGGTTCAATTCCTGGCCTTGCTCA
GTGGATTAAGGATCCAGCACTGCTGTGAGCTGTGGTGTAGGTCACAGACA
CAGCTCAGATCTGGCATTGCTGTGGCTGTGGCCTAGGCTGACAGCTATAG
CTCCGATTAGACCTTTAGCCTGGGAACCTCCATATGCTGCAGGTGAGGCC
CTAAAAAAAAAAAAAAAAAAAAAAAAGAATAAATCTATCCTGACACCAAAG
CACAAAGGAGATCCTGTGGCCTCAGGAAAGATAAGGAAAGGGGGAGAAAG
GCTGGAGGACCAGTCTAGGGAAATCCTGGCTTCAGTGCCCCAGGAAGACC
CCCATCTCCCTACCAGTCCTAGGAGGTTGAGAGGTTAGTCATGCTGAGAA
AGAAGTGCCCAGCAGTGTTCACCTCACCAAGTTGCAACCATTGGTATCTC
CTCCTATTATTGTCCGGGGGAACAGGCTCTGAAGCCCTCGGATGTATGAA
AAGTAGAGGATTTATCAACACAGTGGGATGGTCTAAGCAGATCATGTGAT
TTTGCATGTGGCTGAAACAAAAATTGCCTGGGCTCGTGATTTAGTTTGGA
AAGAGGAAGACAGTTGAAAGAAAGGACATTTCTTTTCTGTGACACTATGT
ATGAGGTCTCATGGTTAACAGAACCAGAAGGGCTGAAGTACCTGTGCATG
GTGCTGCCCATCCCCCGCTTCCCTGGGAGGAAGAGAAGGAGGAAGAAGAA
AGGGACAAGGCATCTTGAGGGAGCAATTATTCTTCCCTGGCTCTGCTTAA
GTCCTGGGTTGCTGCTGCATTCAGACAGAGCATCTCCAGACAGAGTGCTG
GGAGCAGAGCGAGGAGGTAGCTGTTGTGGGGAGAGGATGTGGGTGGAGCA
CTT**AC**CACGTGTCCCAGGAACAGGAG**CT**GTAAGAGAGAAAGCAAAGATCA
GCCTGGTGTTTGGATTCATTGATTCATCATTATAAATAATCACCTCCTTG
CTGATCCCCTGAACTCTCTTGTCCTTTTCTGGCTGCAATTCATTCACCTG
TGTTGTCTGTGCTTTTACTTCTTCAGTTATGTGTTGATTAAATAACCACA
CAGGAGGAGTTCCCGCTGTGGCTCAGCGGAAACGAATCTGACTAGAAACC
ATGAGGTCATGGGTTCCATCCCTGGCTTCACTCAGTGGGTTAAGGATCCA
ACATTGCTGTGGCTGTGGCTGCTGTGTCTGTGCTGTGGCTGTGGCGTAGT
CCAGCAGCTGTAGCTCTGATTGGACCCATAGCCTGGGAATCCCCACGGCC
CTAAAAAGCAAAAAATAAAAATAAAAATAAAACCCACACAGGAGTTGGAT
TGGTATATGTAAATTTACTGTCTCCATATTCAAAAGGACACTGAACTGCC

ATTCTGACACTTTTCTCTGTTAAATTTGCTCTTCTGTGCTTGATAGTGAC
ACTCATCTTTTCCTGCTTAATGGCCGTGCAGTGCAAGGAAGCATGCATGC
TCCTCTGGTCCCACAGTTGATGATTCTAGAATTGCCTGGTGTTTACATTC
TCTGAGCCCCATTCCATGGATAACCGAGACCCTCTTATGCTTTTTCCCAG
GGGAAATTCCTTACACCTAAAGAGAGTAGTTGCCTGGAGTGGTGTCAGGG
GCACAGAGAATGGCCACTGCACCCCACCACAGTGTATCTTCCCTCCACCT
GCTATTTGCATAAGACAGCATTAGCCTTCCCACCTAAGATGTGGTCCCAG
TGAAACACACAAAAAAAGGAAGGTAAACAnnnnGGCCnCTCATnAGnCTT
TCAAGTCTATGACACTAGACTTTCCTCAGAGCTGGCTCTGCTGTGCAGGG
TGGCAGAGCACCAAGGACATGTGAACCCAGTGAGATTGGGCAAAAGACTT
AAAAAGAGATTGGCnAAAAAAAACTTCCTGATTCTGCCAATTTCACAATT
TGCCAAAGCCAGCAGGGTGGTCAGAATTTGATTTCCATCAGCCTGCCCTC
ACCCTCCTGAnTTnCCTGACGTGCTTCCCATGCCCCCCAACTCCTCAACT
TTGCCCAAGCTCAGCCCCAACCGAGGTGGCAGCAGCTGAGTTAGAAAAGC
ACACCTGCCTCTGCTCCTGCTCCACCGTCCCTCCTGGGACTCTCACTC**A**
CCCAGGAATAGCAGAGCTGTCCACAGGAGCATGTGGCCCAAAGGATGGCA
GGGCATGCAATCAGCCCAGTCACTCCTGGCAGnGGGGAGGGCTAGGAACG
AGGGGATCCCCATCACCTCCTGGAGTACGCAGGTTGCCTCTGGCAGCGCC
AGCGCAAGACAGGCTTCTCTCAAGGCTCGACAGATTCCACCCCAGAGGAC
AGACAAATTCTGGAGAGCAAAAGAGGAAGTGAAAAGAAATGTTTTCTCAT
TAGCTCCTCCCCTCCACCCTCTTTCATCTCAACTGTCTCCTCCCAGAAGA
TTCAGGGCTTCCCAGAATGGATGTTCCTCAGTCCCACTCCAAATTCACAG
AAAATGTAGAAATTGGGAAACAACTTCTCTTCCTCTGGGGCCTCAGCTGG
GCTCAGCCTCTGGGAGAAAGAGGGAGGGGACTAGTTACTCAAACTCAGCT
CACAACCTCCTGTCTGTCTCTGTCTTTTTTTTTTTTTTnnnnnnTTTTTG
TCTTTTTGCCATTTCTTGGACCACTCCTGTGGCATATGGAGGTTCCCAGG
CnAGGGGTCGAATTGGAGCTGCAGCCACCAGCCTACACCAGAGCCACAGC
AATGCCAGATCCGAGCCGTGTCTGTGACCTACGCCACAGCTCGTGGCAAA
GCCAGATCTTTAACCCACTGnGCAAGGCCAGGGATCTAAnCCACAACCTC
ATAGTTCCTAGTCGGATTCGTTAACCACTnCGCCACGACAGGAACTTTCC
CTCTGTCTCTTTAGAACAGGCGATGGAGGAGAGTGACCCCCCAGTCTTTC
CATACTTCTAGATCCATTCAGAGGCCTAGATGTTAGnGGCTACATGGGGG
CTCATTGTTAAAATGGCTCATTATGCTGACCCAAGAGACTGACCACCATG
TTCAGATGACATCTGGTTTTCACCACTGGTGCCTCCTTTCCCAAGACTAC
AAGACCCCCCACTGTGGGACACTACACACCTCCAGCTTTCTCAAGATTAT
GAAACCACCCTACCGTGAGACACCTCTGACTTTCTCATGACTATGTGACC
ACCAGAGTCCAGCTGCAGGAGAAAGATAAACTAATCTCCCCCGGCTTCAG
GGAATACAATTTCCCATCCACAGAGTATAAGAAGGCCCCTGGAGAAGGGC
TGGGGAATGGCTCTTCTCAAGAGTCAGTCACCCTCTCCTTTTCCCTCTAA
TAAATTTTCTTTTATTTGCCTGAATACCTGGCTCGTTCTCGTTTTCTCCA
CACTCACCTTACACTAGACACCTGCATCAGATTCCTCTGGAGGCTGAGCT
TGGCGGCTTGAGACCAACCACCATCATCATTTGATTTTCTTTATTATCAT
AATCCTGACTCTGTTTTGGATTGTGGAACAAGGACAAAGAATATGTCTGG
GTGGCAGTGGGATGATCCCACTCACCTTCTTATTCCCCTTTCATCCATTC
TTTCACTCATTCCTCTGACAGATATCTTTTGAATAATAATTTGTGCTCTG
GTTCTTTATCTTTCCCACCCTCATTCCCATCACTGTTCTTATCTCTATGG
CATGATGAAAGAGCAGAACTTTGGAACTGTATATGCCTAATTTTGAATGA
CAGGCATCTTCTCAGAACTACAGATTCCTAATCTGTATAAATGAAGATTA
TCATACCCACTGCATTGCAAATCAACTCTACTTCAATTTTTTAAAAGACC
AAAAAAAGTGAAGAATATTATGCCTATCCTTCAAGCAGGATTTCGCAGCC
TCAGTACTCTTGATATTTTGGGCCAATTAATTCTTGTGAGGACCTGTTTT
GTACCTGTAGGATGTTTTGCAGCATCTCTGACCTCTACACACCAGACACC
AGGGACACTTCCTAGTCATGAAAATAAAAACGCCTCTAGACATTGGCAAA
TGTACTCTTGGGGCAACATTGCCCCTGGTTGAAATCCACAGCACTCAAGA
TTTTTAAGGAGTTTCTGCTGTAGCTCAGTGAGTTAAGGATCTGACTTCGC
CACAGCTGTGGCATAGGTCACAGCTGCAGCCTGTATTTGATCCCCAGCCT
GGGATCCCTATGCCGTATGTGCAGCCGGAAAAAAAAAAAAAAAAAAAAGA
TTTTTGAAAATTAAAAGAGAATTTGTGTGCTAGCAAATTGCCTGGTGCCT
TGTGGGTATCCAGTCATGCTAATTTCCCTTTTTTGCTTTCTGTCACCATC
TTCACTATCATTTGTCCACCTTCAACATCCCTAGCACATGACATAGTCTG
GGATCTCTCTTGGGGCCTTACCTCAGACCTCTTCCTCAAATGTCTTCTTA

GGAATCAAACTGTAATACTTAAGGAAATCGTGTCGTAAAACCAATTCTCA
CACTCTGTGGTCTTCAGAAACCTCAACAGCCCCACCCTCTTGTCCAAGAA
TTTTACTACTGGCTCCATAGTATCCACCCCTGCCCCCAATCTACCTGCAG
CCCTTACCTGATTTTTAGATGAAAACATTTCTTTATCAGTGACAAGACTC
CAAAGAATTGACATCAACACTTTAAAATAGCATTAGAAATTGATAAAGTT
AATTTACATATCAAGACCTGTAAAGCAGGGGCTGGCCCAGCCAAGTTTAG
ATAAATTACAAACATAAACTTAAGTAACATGCACCAATCATAATCATCCA
GCTCAGCTTTAGCCAGCTTACTTAACCCTGGAAACTACAACCTGCTAGCC
TTTTAAGGAAACTCCTAACTTCCTGGCCAATCATGTCCTGTTTCTGAATT
GCCTCTTCTGAATCTCCATAAAACTCACTCTATTCCAAATCCCTGGGGAA
GGGCTCTACCCTGCTTGTGAGGAACTGTTCATACCCAATCCATGGCCTGT
TTTCCCTTGAATAAAGGATCTCTAACTTGGAGTTCTTGCTGTGGGTTAAA
GATCTGGCTTGTCTCTTTGGTGGTACCAGTTTGATCCCTGGCCCTACACA
GTGGATTAAGGATCCCACAGATTAAGGACCCCGTGTTGCTGCAGTTGTGG
TGTAGGTTGCAGCTCAGGCTGGGATTTGATCTCTGGCCCTAGGAACTTCC
AATCCATATGCnGnnnGnAnnGnnAAAAAAnnAAAAAAnAAAAAAAAAAA
AAAAAAAAAAAAAAAAAAAnCCACACTCGAGGCCTACGAAAGTTCCCAGG
CTAAATGCTGAATTGGAGCTGTAGCTGCTGTCCTACACCGCAGCCACTCC
AGATCAACACCTGTGACCTATGCCACAGCTCAGGGCACAGGGGATCCTTA
ACCCACTGAGCAAGGCCAGGGATCAAATCCACATCCTCATAGATACTCAG
GTATGTTACTACTGAGTCACGATAAAACTCCACATCTGACTTGTTGCTAA
ATTATTTAAGTTTGGGCATTTGCTAGATCAGGCATTTCTCAATCGATTAT
GTAGGAGAAAATGACCCAATAAAAAGGTAGAAAGGAGACACCCTGGCCAT
AATGACTAAACATTATAAATCCAGACAACTACCTCTAAGCACCCCTACAC
ACGCATCTGCTTCTATAAACTTGCTTCTGCATCTACTACCTTGGCTGTCC
CCATTCCAGTTCCTCCAGCCAAGCATGGACCCAGAAGAACTAGGCCATAA
AACCTTGTGAAACCTTCCTCGGGTCTCAGACTCCAGAGAGCGATTTCCTC
TGAGCCCACTGGCTTAATnAACCTGAGTTCTCCAACTCTAAAAGAGCTGC
TGGAGCTGATACAnTGnGGCCACAGGGnnnCTGnnnnnnnnnnnnnnnnn
nnnnnnnnTTGCTGGAGCTGATACACTACAGCCACAGAGCTGCTGCTGGA
GATGATACACTGTGGCCTCAGGACTGCTGCTGGAGCTGATACATTGAGGC
CACAGGGTTGCTGGAGCTGATACACTGCTGCCACAGGGCTGCTGCAACCG
CAGCTGCAACCGCAGTGGCCGTATCATTGCACTCCACAAATTTTTTTTTT
TTTTAAAGAAATTGAAGCTTGTGGCAACTCCACGTGAAGAATCCTACCAG
CACCATTTTTCAAACTGCATTTGCTTGCTTTATGTCTGTGTGTCAAGTTT
TGGTTATTGTCACAATATTTCCAATTGTTGGTTTTTTTTTTTCCCCTGTG
TGAAAGGCCGATTTTTTTTTTTTTTTTTTTGGTCTTTTTGCCATTTCTTG
GGCCACTCCCACGGCATATGGAGGTTCCCAGGCTAGGGGGTCTCATCGGA
GCTGTAGCTGCCAGCCTACACCAGAGCCACAGCAATGCCAGATCCGAGCC
ACGTCTGTGACCTACACCACAGCTCACGGCAACGCCGGATCCTCCTATAC
CACAGCTCACGGCAACACCGGATCCTTAACCCACTGAGCAAGGGCAGGGA
TCAAACCCGCAACCTCATGGTTCCTAGTCAGATTCGTTAACCACTGCGCC
ATGTCGGGAACTCCATGGCCGATGTTTTAATTGCCATGACATTGTGAGGA
AGTGTCAAGTCCAAAAAAATAAGCCCAAGGAAAAGCACATTTGACAAATA
CCTAACAGAAGCTGGTTACTGCACCTGCAATGGTGTTTTTTGTTTTTTGG
TTTTTTTAATTATTTATTTATTTTTTATTACCCAAATGAATTTATCACAT
CTGTAGTTATATGATGAnnATnnCAATnnCCAATTTCACTGGATTTCCAT
CCCATAACCCAAGCACATCCCCCCACCCCCTCCAATTTTTTCTTTATTAT
AATGTTTGTTAAGGTGATCTGTGATCAGTGATCTTTGATATTATTGTAAT
TGTTTCAGTTTTTTTTTTTTTTTTTTTAGTGGTAGAGATTCCCAAATAAC
TTTATTGACGGATTCAGGGCAAAAAAATGCCACTACCAGCACTGCAGGGC
AAAGGGGAATCCTGGAAGAGGAATGACGTGGTATAACAAAACACACAAGA
CTCTTAAACGTTTAGAAAGTGGGGGAACTGGAGGAACTCTGTTTCGTAAA
ACAAAGGTGCTGGGCTTTAGCCCACCTGACTTTTATTAAGGAAGGTGCCA
TATGTAGATTAGTGAGCAGGAACAGGCATAGGCAATGACAAAACTACCTC
TTAGTGAATAACAAAGGAAACGGTTAACACTGGTGCATACCTCTAGGGCA
TAGTGCAGCTGCTTATAGAATAGCATGATTTATGCATAACTTCCTTGTAT
TGTCTTTGAAGGAGACCTTGTACTTTAGAACTCTGCCTCAGCAGATATTT
GCCTTCTGCAGAGTTCAGCAGTTCAGTGGTCTCCAACAGCAAAAGGGTAA
TGAAAAATTAAAGACACATGAGGTACATAAAACAAAAGACTGTAAGACTG
AAATGGCACCTACTACTCCCTTGGCTCCTCCCTTACTAGTTCCCACAGTC

TCTATCTGAATTGCCTTTCCACTCTGAAGAGATTGTAAGACTGCAAACAA
AACTCTACCAGTTAGTGGCCTTACAGACATCTCATATCCACCCTCCAAGG
AAGGCTACCAAATGAGACCCTCTGATAAACTGCTACCAGTTATTCCCCTA
AACAGCCAAAGGCAAACTAAAATTACTGGAAAACCTTACCAAAGTCTGAC
AGATACCATAACTACACTCTAGCCCTCTCTTGCTCCAGAGTCTCCGGAAG
GAATTCTGGATAAATTGGCAGAAGCTGCTGAAGGGTGAGAATTCTTACCA
AAGTCAGATTTTCCAGCTCTCTGAAGTGCTTAGTCAAGAGAAGAATATAA
AATTTTTTGTTTTTCTTTCCTTTCCAAATTCAGACTTTTGTGAATTTGGT
TTTGGGGTAGCCATTGGTTGTCAATCCTTTCTCTTCCAGGGAAAGATACT
GCCTTTTTGTTTGTGTTGAAAACTTGACTTGGCCAATATCAGGCTGTGTT
AAGCAGATAAGTTAACCCAGGAGAAAGAGGACCAGGAATAGCTTTCTTGA
CAAAAGAAAAGCCATTTTGGCCTAAGCCATTTTGTGATCTAAGCCTGACT
ACAATGCTTGCCCTTTCATAAGTCAAAGTAATTAATGACCTCAAGGGAAA
TAAGAGAATGCAGGAACAAAGGAAAGGCAGTCAAACAATAGTGCAGTGAT
AAAGCCAAATCCTAACAATAATATGTCTTTGAGTTCTGCAGAAACTAAGG
CCCCCACTCAGGTGGAGGGGGGAAAGATGACATTTTCTCTTAGGACTTCT
ATCAACTAAAGCTTGGACTCAGTTGACATTTGCCCCAATTCTATCTGCTC
AAGCCCCTTCATGAATATGCATGTCTCCTTAGCTTAAAACTTCCCAACTT
TGTTGTTCTAGGAGGCATGAAGATTATCATGCCTATTCTTCAAGGATTTC
TCAACCTCAGTACTATTGACATTTTGGGCCAATTAATGCTTGTGAGGATC
TGTTCTGTACATTGTAGGATCTTTTACAGTATCTCTGACCTCTGTACTAG
ATGCCGGGGGCACCTCCTAGTTGTAGAAAATAAAAATGCCTCTAGACATC
AGCAAATGTTCCCTTGGGAGCAACGTTGCCCCTGGTTAGAACCACCGTAC
TTAAGATTTTTGAGCAGATCCCATTGTGGCGTAGTGGGTTAAGCACCTCA
CATTGCTGCAGCTGTGGTATAGATCACAGCTGAGGTTTGGATTTGATCCC
TGGCCCTGGAACTTCCCTATGCCATGGTTGCAGCTGAAAATATATATATA
TATATATnnTTTTTTTTTTTTAATTAAAAGAGAATTTGTATGTTAGCAAA
ATGCCTGGTGCATGGGGAAATATCACTGATGTTCTCCGTACTTGTAGCAA
GTAAAAATAAATTCTTTCTTCTCCCTTGCTTTGATTTGGTTGTGTCTTTT
TGATGGGGACGTAATAGAATAGTTAGACAGGTAGTTGTGGAAGTCCCAGT
AAGGGACCATAGATAGGGAAACCTAGGGAACTTTGAGACATCACTGTTAT
AAGAAAGCCATCAGAATGTGTCAGGATGGAGTGAGGCCTGCATTGAGGTT
CAGACCAAGGGCAGGAGTTTAAGGACCACCCTTCTGCTGATTTTAATAAG
TGCCATGGCAGCCCTAGTTTGACCTTATACCTTATTACCTGATATCTAGG
ATGAGCTACTATGAGAAAGAGGAAGAGGTGGTCTTTTCTCAGCCCCACTC
CTCTTGGATTATAAAAACTGTACCCCACCTAATCCTCAGGGCAGAGCCTC
CTTGCCTGCTCATTTGCCTCTCTCACAAGCATCCTACCTTAATAAATCTG
TTTCTTGCCTATCACTTTTTCTCTTACTGAATTCCTTCTGTGCTGAAACA
CAAAGAACCTGAACATCAGTAAGTCCAGACACCAGGTGAATTATTCTAAT
TCAAAATCCATGGATTCAAGCTCCAATCTGGGTTTTGGCTGGATTCAAGT
CCCAATCTGGGTTCTGGCTAGGTTCAAGTCATTCATGCCATCAGTTTCGT
TTTGGCTTGACACTCACCAGGAAGTGAACCCAATTTCCGAGTAACAGTTG
GGGGCCAAAAAATGTGGCCAGGCAGAAATGTGTTTGTAGTGTAAGCTCTG
ACAGTATTTTCAATTTTGGAATATAAGTGCCTGACTTAAGCTTTTGATTG
CCAAGACATCCATTCAAAAGGCAGCCCCTTCAAAGAAGGCTATCTCAGTT
AGACACAGTACCAACCACACGAGTAGATAAAGAGCCAGCCCACGTTCTAT
GGCCTCCTCACCCACTGAGGATTCTTAGTTTTAGAGATCTTCATGGATTT
CAATAAATGACCCATTGGGATACATGGGGTTTTTGTTGTTGGTTTTTTCT
CATCCTGTGTTTTATATTCCTCTTATGTTTTAAATTCACCCCAAAGAGTG
AGCTCCCACCCAAAACCCCAGGCACCCACCCTCAACCCTAGTAAAAGCAG
AACCCATGCCCATGTGCACTCTCTGACTCCCTATGACCTTGAAACAGGAG
GGAAGCAGGCAGGGCCTTTTTTTTTTTTTTTTTTTTTTTTTTTTTTTTTT
TTnTnnnnnnnnTCnnTnnnnnTnTnnAGnnnnnnnnnnnAGnnnTnnnn
TnGnnnnnnnnnTTnnnnGnCTnCACCnGnGnnAnAnnnAnnnnnnnnCC
AAGACGAGTCTGCAGCCTACACCACAGCTCACGGTAAAGCCAGATCCTTA
ACCCACTGAGCAAGGCCAGGGATAGAACTCGCAACCTCATGGTTCCTAGT
CAGATTTGTTAACCACTGAGCCACAACAGGAACTCCAGGGCATAACTTTT
TAAAAATTACATAGCCATTGAGTGTGGAGAGACATCTTCCTGATGTATTT
TCTAAGCCCTAAGGAAACTATAAGTTGGAAAAGTGCTTATTCATGCTGAT
GTGGAATTTAGCTATAAGTAACTATAAATAAGGGGCTAGAGGGAACTTGG
CTTGGCTCAGCATTAGCTGAGAGGACAAGGTGTTGACCTTGGAAAATTGG

GTCAGCTCAAGTCAGAAAACTGCTCAAGCAAGCACTGGTTGTTCCTCCAG
ATGATATGCATGGATAGATAGCTGACTCCAGCCTTATGGATGCCCTTTAC
ATATAGAGTGTAAGAGTAAAGGTCAGAGTTAAAATACAAAGTGACTGGAG
GCAGAGGGCCTTGGGGTTTCTATTTTCTCACATTTTTTACATTCCTGAGA
GCACAATAAAGACTATGAGGCATCAACATGACATTTGAGGGTCTTGATCC
ATGAGGTCTTGAGTTCCCTGGTCCCATCTTTAAACTTTACTCTGTCTTTC
ATTGTTGCCATTTAACTTTTTTTTTTTTTTTTTTTTTTGGTCTTTTTGCC
ATTCCTTGGGCTGCTCCTGTGGCATGTGGATTGAATCGGAGCCGTAGCCG
CCGGCCTAGCCAGAGCTACAGCAACGCAGGATCCGAGCCACGTCTGCAAC
TACAGCTCACGGCAATGCCGGATCCTTAACCCACTGAGCAAGGGCAGGGA
TCGAAACCGCAACCTCATGGTTCCCAGTCGGATTTGCTAACCACTGAGCC
ACCACAGGAACTCTCCGTTTAACATTTCTTAAGCTTTTCGGGGCCGCCCT
TCGGCTCGCTCGAGCTGGTnnCnnCnnnnnnnnnnAnAnCnAnAnCnnnn
nnGnAnCCnnnnnnCnnnnGnnnnnnnAnnnnnnGnCnnnnnnnGnnCCT
TnAnCCnCnnnnnAnGnnCAnnGnnnGnnAnnnnnAnCTnAnnGnnnnnn
nnnnnnnTnnCnnnnCAnTnnnnnnnCnnAnnAnnnnnnnGnnnAAnnTn
TnnnnnGnnTnnnnnnnnnGnnnTnnnnnnnnnnCnAnnnnnnnnnnnnA
AnnnAnnAnAnAAnAAAnnnnGnTnCnnnCnnnnnnnnnnnnnnAnCnnn
nnnGnnTnGnnnnCnnGnGGnnnnnGnnnCnnnnnCTnnnnTnnnTnnnn
GGnnTnAGnAnCnnnnnnnnnnGnnnnCnGnnnnnnnGnnnGnnnnnnnG
nCTnnnATnnnnnGnnnnnnnGnnTnnGGTGnnnnnnnnnnnnnAnAnCT
nnnAnnnAnnnnnnAnnnnnnnAAnnnnnAnAnnnnAnnnnnnnnnnnnA
AnAAAnAnnAAnAAnnAnAnCnnnAnnnCnnnTCTGAATAGTTGGAATAA
TCCTCCCACCTGTTAGCATATGAAATTACCCAGCCTATAAAACTAAACCA
CACTACATTTCCAGGCTGCTCTCTCACATTTTGGGATGGATGGACTATGT
TCTGTCTATGGAATGTATATCTTCCAGGCTGCTCTCGCCTTCTGAGATGG
CCCACACTCTTATCAATAAATCTACTTTTACTTATCCCTTCCCTCTCACT
AAAAAGGCCTTCTGCACTTAGATGTAAAGAACTTGAACTTCAGTAAGTCC
TGAGACTAAGTGTATAATTTTAATTAAAAGACAATGAGTTCAAGTCCCAG
TGTGAGCTTTGTCTAGGTTCTGGTTCCAGCCCCTAGCTTTAAGTCCCAAT
CTGAGTCGTGGGGTTTCAGTCTCACTGTGTTACCCAAAGTGTGCTATGCA
ATTTCCAGGTCTTGGCAAGTAATACGTCTCTATTTTTTTCAGTTTCCTAG
TAGTTATTGCTCAAGGGCACCTTGCAATGATAAGAAGTAAAAGAACTGGT
CCAACAACCACACTGGTTATTGATAGAAAGACAAGCACAAAACAAGGTTG
CACCCCATTTATAGCTGGGGTCCTACTGACTTGACAGCTCTTATAGGAAT
TATCTTTCTCCTGGCAACTTTAGTGAGTGGCTCTGGATCTTGGGAGTGCT
AGTATGTTTTGCTCTCTAGTTTTTAAGGCCTTCTCCCTAAGCTAAATGGC
TCTAGGTACCCAGAGGACAGAAAAACGTTCCCAAAACAAACAGCGATAAA
TATAGAACATAAGATTTTATTTATTTATTTATTTATTGGCCCCACTCGCA
GCATGCATCCAAACTATGCCACAGCAGAGACCTGAGCCCCAGCAGCAACA
ATACTGGGCCCTTAACCCACTATGCCACCAGGTAATTCCCATGGATTTTT
TTTTAAGTTGAGTAAATCCGTATTTTACTTATTGGTTTATTTTTATTGAA
ATACAGTGATTTATAGGATTCTTTAGGATTCTTTTTTTTTTTTTTTCTTT
CTGGCCATACCTGTGCCATGTGGAAGTTCTCAGGCCAGGGATCCAAACCA
CACCATAGCAGTGACAACACCAGGTCCTTAACCCTCTGAGCCACAGAGTA
ACTCTTACAGGATTATTTTATTTTATTTTATTTTTATTTTTTTGGTCTTT
TTAGGGCTACACTCTTGGCATATGGAAGTTCCCAGGCTAGGGAATCAGAG
CTGTAGCTGCTGGGCTATGCCACAGCAACACAAGATCTGAGCCACATCTG
TGACCTACACCACAGCTCACAGAAAGGCTGGATCCTTAACCCACTGnGCA
AGACCAGGGATCGAACCTGTGTCCACATGGATGATAGTCAGATTCGTTAC
TACTGAGCCAAGACAGGAACTCCTCTTTTAATTTCTAACTCAATTGAAGA
GTTTCTCTTGACATAAAGAAGCAAATCGAAGACAATGGAGTTCCCATTGT
GGCTCAGCAGTAATGAACCCGATTAGTATCCCAGGCTGGCGGTCCAATCG
GAGCTATGGCTGTCGGCCTACACCACAACTACAGCAACGCCAGATCCAAG
CATGGATCCAAGACATCTAAGCCATGTCTGTGACCTGCACCAGAGCTCTC
AGTAGTGCTGGATCCTTAACCCGCTGAGCAAGGCCAGGGATCAAACCCAA
AACCTCACGGTTCCTAGTCGGATTCACTTCCCCTGCGCCATGATGGGAAC
TCCCATAAACTAGTGAGTTTTGCATTATCATAGTGTGACTAAAATTTTTT
AAGGGGATCTATAAGATTTCTGTGTCTCTACATATGTTTGTCTATATACA
CATGTTATAAATATGTGATTTGTTTCCAACTCTGGTTCATGTTGCCAAAA
TAAATTTGTAAAAGAGCTCTATTTAATTGGCTGAAAGAAAACTAAGCAAT

TATATAAGTCTAGTATACTCTCAGAAATAAGGAAATTAACTCAAATATTT
TTCAAGTTCACATGATCTAGGAGAACCTTTTGTAAATAAAAGCTAGTTTA
GTTTGTCAGTTTAATCAAAACAGCCATGTCTTTATAGTTATCAGCATTAA
ATATGATACTTTGTAACTGAACAGGACCCAATGGGGCCTTTTTGGGACAG
ACCCCTTCCTCATATCCTTTGCTTTAGCTCCTGTCTAAAATCCTTAGATA
ACAATATCTGATGCCCATTTCCCGATTTGTTTTGCAGATGCTAAAACCAC
CACCAAGTGGGAGAAATTAACTACATGATCCTGAGCAGTAGCTTCCAGAC
CTACTGGCACTTTAGGACTCATAGTGTTAGCCCTGCAACATTGCCATTAC
TTCACCACCTCCAATCAGAGAATTGTGCATGAGCTGTTCACATATCCTGG
GATACCCTGCCCTTACTTTGCCTTTAAAAATGCTTTGCTGAAACCCGTTG
AGGAGTTTGAGGTTTTTGAGCACTAGCTATCCTAAACTCCTTGCTTGGCA
CTGTGCAACAAATGATGAACTTTCCTTCACCACAGCCCAGTATCAGTAGA
TTGGCTTTGTTGAACACAAGCTAGTGGACCCAAGTTTTGGTCAGGAACAC
TTTTATTCTACCTGCATTTACTAAAAGTCAAATAAGTTCATGTTATCTCT
GTTACAGAATTTGTCAGCAAGAAAGATAACTTAAGATGAAGCTTAGCTGT
TTAATGTCTCATTAAATTTTTATTTATTTATTTATGTCTTTTTGCCTTTT
CTAGGGCCGCTCCCGAGGCATATGGAGGTTCCCAGGCTAGGGGTCTAATC
GGAGCTGTAGCCTTTGGCCTATGCCAGAGCCACAGCAACACAGGATCCGA
GCCACGTCTGCAACCTACACCACAGTTCACGGCAATGCCGGATCCTTAAC
CCACTAAGCAAGGCCAGGGATCAAACCCGAAACCTTGTGGTTCCTAGTCG
GATTCGTTAACCACTGCAACACGACGGGAACTCCTCATTAAATTTTTATA
AGCAACCTAAACATAATCGTTAAGAATAAATTAGATATGGTACAAGTTTA
TAAGTAAACTTTTTAAAAACAATTATGCTTTATGATACATCTACTTAAAA
ATAGTTTCTAGGAGTTCTCATTGTGGCTCAGTGGGTTAAGGACCCAATGT
TGTCTCTTTGAGGATGTGGGTTTGGGTTCAATCCCTGGCCTAGCTCAGTG
GGTTAAGGATCCCGCATTGCAAAGCAGCTCAGATCTAGTGTTGCCATGGC
TGTGGCATAGGCCCCAGCTGCAGCCCCCATTCGACCCCTGGCCTGGGAAC
TTCCATATGCCGCAGGTATGGCCCTAAAAAGAAAAAAAGTTTCCAAAGTT
TCCAAAATGTTTTTGGTAACTTAAAATCTTAAGTTTTGTGGGGTTTTCTT
TTGGGGGGGATTGTTTTGTTTGTTTGGTCTTTTTTCAGGGCTACGCCAGC
GGTATATGGAAATTCCCAGGCTAGGGGTTGAATCAGAGCTGTAGCTGCTA
GCCTACGCCACAGCCACAACAATGCCAGATCCAAGCCTCATCTGCTACCT
ACACCACAGCTCAGGGCAACACTGGATCCTTAACCCACTGAGCAAGGCCA
GGGATCAAACCCAAGTCTTCTTGGATACAAGTCGAGTTTGTTACTGCTGA
GCCACAATGGGAACTCCAAAACCTTAACGTCATACTAAAATAAATGATAG
ATATTCATTGAATATTTTTATCATTTGCAAGTAAGATAAAATACTGAAAC
ATTAATTGCTGAACATGGGTTTATCTGCTTTTGGCTTCCCTTTACAGAGG
AGCTAAAGATATTTCAGTCTCTTAGTAAACATGGTTTCTGCCACACTGAA
AAGTTTTACTATGAGGAGGAATATACTTCTAGAAATTCTAAAATGTATTT
ATATATTTGCCAGTCCACAAGATGCTAGTGCTACAGACAGTCCACAGTTT
CTCACTTTTTAATTTTCACCAGGAATTAAGGGATTCTGAGGGTAAAAGTT
ATAATAAATACATGTAATTAAAACTACTAGAAATAATATCAAAAATATCT
CTATGCAAGGTATGGAGATGTGTTTTTGTTAAGGAAAAAAGAGTAATTTT
GTCCTAAAGTAAAATGATAGTTCCAGAATTAGAAAGAGAAAAAAATTAGGG
AAAAACTTGAATGGAGGTAAAACAGTTGTAAAAAGTTTGTGGAAAAGTAT
TTTGGTGAAAGGAATTTTAAGTGCAGTTAAGCTGGAAGGTGTGGGTAAGG
TTGGAACAGATTTATTTAAGTTTTTTAAAATGTTAATATCAAAAATAGAC
CCATGCAAAATTAGACTTTGGTTTTCTGTTAAAAAAAAAAGAAGAAAGTC
TTATTGAACTGTCAGTTTGCTCTTGATAAGAGTGTGTGAACATTTCTTTT
TAATTTTTTTACTCTTTGGGCAGTCTACTTGGACAGCAAAGATTTTGAGT
TTTACTAAAATAATTTCTTGTGTTTTATGTTGTTATTTATCAGCTCTTTA
ATTACTTAAGAAAACCCAGTCTTCCCAATATTAAAAGAGCAAAGATTTAT
ACACAATATAGTCTTTTGCATCTGCCTTTAAAATCATTTATTGTCACTCT
AGTTAAATAGATAATTAAAGGAGTTCTCTTGTGGTGCAACAGGTTAAGAA
TACTGCAGTGGCATGGGTTTGATTCCCTGGCTCAGGAACTTCCACATGTA
TGAGTGCAGTCAGAAAGAAAGAAAGGAGGGAAGAGAGGGAGGGGGAGAGA
GAGAGAGCGAGGGAGAGGGAGAGGGAGAGAGACAGAGAGAAAAGAAGGAA
GGGAGGAAGGGAGGnAAAGnnnnnnnCCCATTGTGGTGCAGTGGAAACAA
ATCTGACTAGAAACCATGAGGTTGAAGGTTCAATCCCTGGCCCCCCTGCT
TGCTGTGGCTGTGGTGTAGGCTCCTATTAAACCACTATCCTGGGTATCTC
CATATGCTGCAGGTGTGGACCTAAAAAGAAATGAAAAAAGAGAAAGATAA

TCCAGTATTATTTCATAATGTTCTGTGATCCTATTTAGTCAAATGTTCAA
ACTTTTTGCCTTCTTCACAAATTCCCCAAATCAAATTCTAAATGGAGTCC
TTTCTTTTTTTTTTTTTTTTTTTTTTTTTnGCTGTACTCATGGCTTACAG
AAATTCTTAGGCCAAGGATTCAATCAGAGCAAATGCAGTCTACACCGCAG
TTGTGGCAACACTGGATCCTTTAACCTACTGCACCAGGGCAGGGATCAAA
CCTGCACCTCTGCAATAAATGAAATCTTTTTAACCTCAAAGGAACTTTGA
GATTTCCCAGATACCCCCTGGAATTCTCAAGGGATTTGTCTCTCGGCTTG
TACAATGAGAGATGTTGAGCCAATTAGGTCTATTTGGTATGTTAAATTAT
ATGTGTTAACATTGTCAAATAAACAGTGATGATAGGAGATCCCTAGTGGC
TGAGCTGGTTAAGGATCTGGCATTGCCATTGTTGTGGCTGTGATTGCTGC
CTATGGCACAGGTTCAATCCCTGCTGGCACAGGAAATTCCGAATGCCACA
GGCCTGACAAAAAAAAGAAAAGATGATTAAAAAAAAAGCCGTTGTAAATC
TTCTTAGGGTATGTATATATATATATA
