# Supplementary figures and images for: The genomic organization and expression pattern of the low-affinity Fc gamma receptors (FcγR) in the Göttingen minipig
Source: Immunogenetics. 2018 Dec 18;71(2):123–36. doi: 10.1007/s00251-018-01099-1 (PMC6327001; doi:10.1007/s00251-018-01099-1)

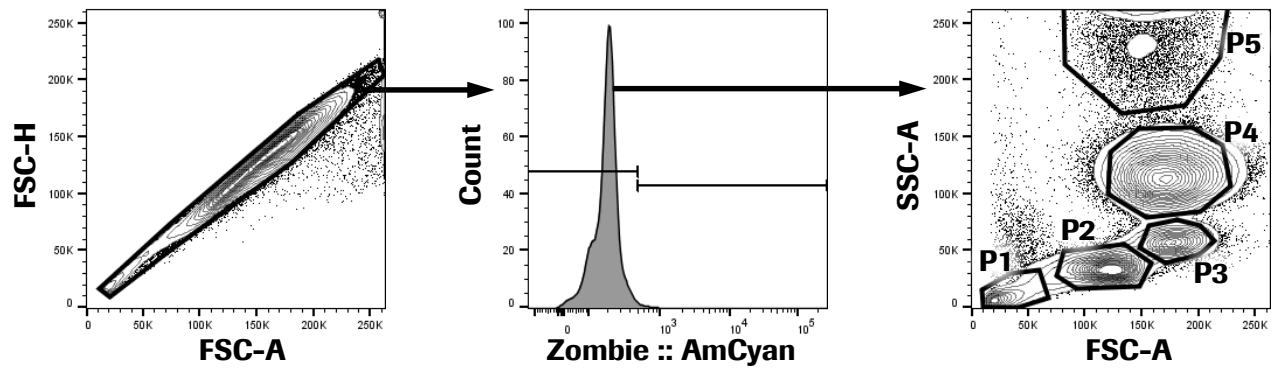

**P1 – Platelets**

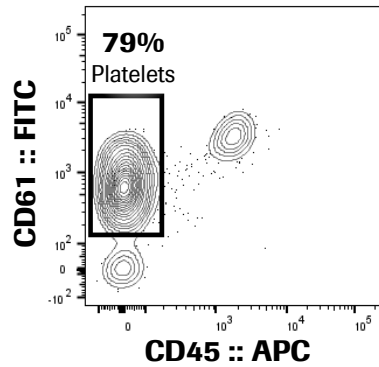

**P2 – Lymphocytes**

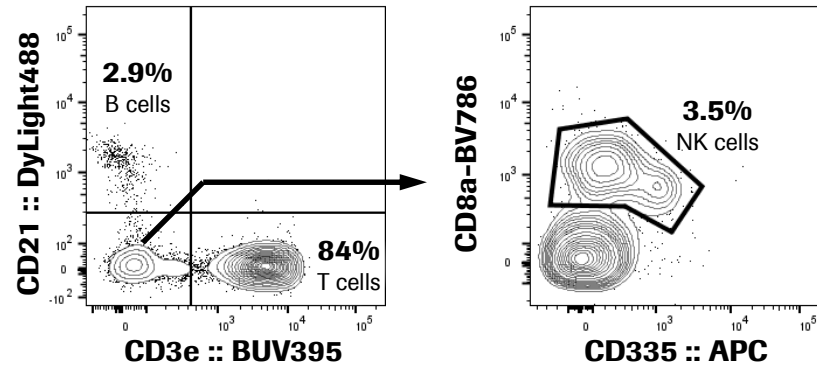

**P3 – Monocytes**

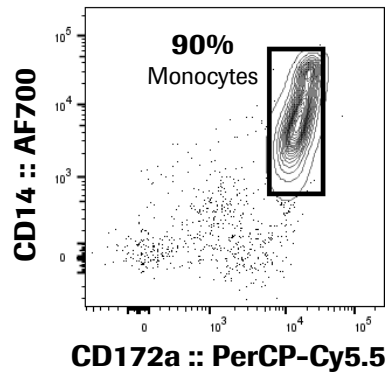

**P4 – Neutrophils**

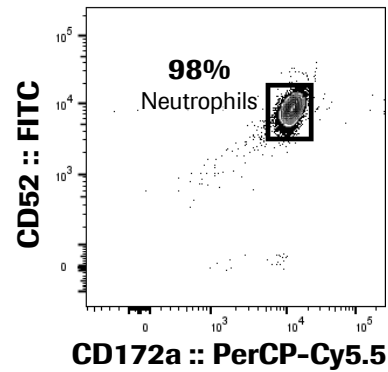

**P5 – Eosinophils**

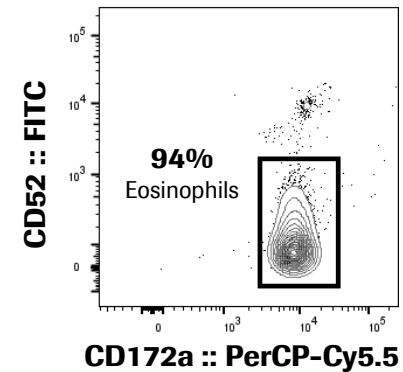

Supplement: Supplementary file 4 — Gating strategy for flow cytometry analysis of minipig blood. Whole blood from Göttingen minipigs was stained with the indicated fluorochrome-labeled antibodies. From single and live cells, gates P1-P5 were selected using forward (FSC) and side scatter (SSC), and cell types were identified using the following antibody clones: CD45 (K252.1E4), CD61 (JM2E5), CD3e (BB23-8E6-8C8), CD21 (BB6-11C9.6), CD335 (VIV-KM1), CD8a (76–2-11), CD172a (74–22-15A), CD14 (MIL2), and CD52 (11/305/44). Numbers indicate the percentage of cells within the respective population (P1-P5). (PDF 196 kb) [file 251_2018_1099_MOESM4_ESM.pdf]
